# Three-Component Reaction of Diamines with Triethyl Orthoformate and Diethyl Phosphite and Anti-Proliferative and Antiosteoporotic Activities of the Products

**DOI:** 10.3390/molecules25061424

**Published:** 2020-03-20

**Authors:** Patrycja Petruczynik, Paweł Kafarski, Mateusz Psurski, Joanna Wietrzyk, Zdzisław Kiełbowicz, Jan Kuryszko, Ewa Chmielewska

**Affiliations:** 1 Department of Bioorganic Chemistry, Faculty of Chemistry, Wrocław University of Science and Technology, Wybrzeże Wyspiańskiego 27, 50-370 Wrocław, Poland; patrycja.petruczynik@pwr.edu.pl (P.P.); pawel.kafarski@pwr.edu.pl (P.K.); 2 Laboratory of Experimental Anticancer Therapy, Department of Experimental Oncology, Ludwik Hirszfeld Institute of Immunology and Experimental Therapy Polish Academy of Sciences, Rudolfa Weigla 12, 53-114 Wrocław, Poland; mateusz.psurski@hirszfeld.pl (M.P.); joanna.wietrzyk@hirszfeld.pl (J.W.); 3 Department of Surgery, The Faculty of Veterinary Medicine, Wrocław University of Environmental and Life Sciences, Norwida 31, 50-375 Wrocław, Poland; zdzislaw.kielbowicz@upwr.edu.pl; 4 Division of Histology and Embryology, Department of Animal Physiology and Biostructure, The Faculty of Veterinary Medicine, Wrocław University of Environmental and Life Sciences, Norwida 31, 50-375 Wrocław, Poland; jan.kuryszko@upwr.edu.pl

**Keywords:** bisphosphonic acids, synthesis of C-P bond, multicomponent reactions, P-containing drugs, anti-proliferative activity, osteoclasts, in vivo activity

## Abstract

A three-component reaction between diamines (diaminobenzenes, diaminocyclohexanes, and piperazines), triethyl orthoformate, and diethyl phosphite was studied in some detail. In the case of 1,3- and 1,4-diamines and piperazines, products of the substitution of two amino moieties—the corresponding tetraphosphonic acids—were obtained. In the cases of 1,2-diaminobenzene, 1,2-diaminocyclohexanes and 1,2-diaminocyclohexenes, only one amino group reacted. This is most likely the result of the formation of hydrogen bonding between the phosphonate oxygen and a hydrogen of the adjacent amino group, which caused a decrease in the reactivity of the amino group. Most of the obtained compounds inhibited the proliferation of RAW 264.7 macrophages, PC-3 human prostate cancer cells, and MCF-7 human breast cancer cells, with 1, trans-7, and 16 showing broad nonspecific activity, which makes these compounds especially interesting in the context of anti-osteolytic treatment and the blocking of interactions and mutual activation of osteoclasts and tumor metastatic cells. These compounds exhibit similar activity to zoledronic acid and higher activity than incadronic acid, which were used as controls. However, studies of sheep with induced osteoporosis carried out with compound trans-7 did not support this assumption.

## 1. Introduction

Although the synthesis of bisphosphonic acids was described as early as the 19th century, the interest in these compounds is still growing, rooted in their promising and variable physiological activities [1]. Most commonly, their regulation of bone growth has been exploited for the design and preparation of antiosteoporotic drugs that can also be used to treat skeletal complications of various provenances [1,2,3,4]. These compounds also exert an inhibitory activity towards important medicinal enzymes, including farnesyl pyrophosphate synthase (thus inhibiting Ras farnesylation, as their structure makes them potential anti-cancer and/or anti-protozoan agents), glutamine synthetase (possible anti-tuberculosis agents), and undecaprenyl diphosphate synthase (antibacterials) [1,5,6,7,8,9].

Some patients who received intravenous and oral forms of bisphosphonate therapy for various bone-related conditions suffer osteonecrosis of the jaw, dependent on the dose and duration of the therapy [10]. The mechanism by which bisphosphonates might contribute to the development of osteonecrosis of the jaw is not well understood [11]. However, it may result, at least in part, from the high binding and long residence of bisphosphonates in the bone tissue [12], with two phosphonate groups and an adjacent hydroxyl moiety mediating the binding [13]. Thus, it has been suggested that their analogues with a lower affinity for bone minerals could be useful. The use of aminomethylenebisphosphonic acids lacking the hydroxyl moiety seems to be obvious solution to reduce the bone affinity and increase the bioavailability of bisphosphonates. We have synthesized hundreds of these compounds and screened their anti-proliferative activity towards mouse macrophage J774E cells [14]. Since these cells originate from the same precursors as osteoclasts, this test is considered a reasonable preliminary test for antiosteoporotic activity.

In this paper, we describe the application of a commonly used a three-component reaction of amines with triethyl orthoformate and diethyl phosphite [15,16,17] We present the reactions with diamines (diaminobenzenes, diaminocyclohexanes, diaminocyclohexenes and piperazines) being used as substrates. Since this reaction usually gives mixtures of products that are difficult to separate, the crude esters are commonly hydrolyzed with concentrated hydrochloric acid and the desired bisphosphonic acids were isolated in moderate to good yields. The anti-proliferative action of these compounds against mouse macrophage-like RAW 264.7 cells, originating from the same precursors as osteoclasts, was screened. As a result, *trans*-cyclohexane-1,4-di(aminomethylenebisphosphonic) acid (*trans*-**7**) was chosen for the study of its antiosteoporotic activity on sheep. Additionally, the anti-cancer potency of the synthesized compounds was studied using the MCF-7 cell line, which is a well-established model of breast cancer, and the PC-3 human prostate cancer cell line.

## 2. Results and Discussion

### 2.1. Chemistry

#### 2.1.1. Reactions of Diaminobenzenes

**Figure 1:**
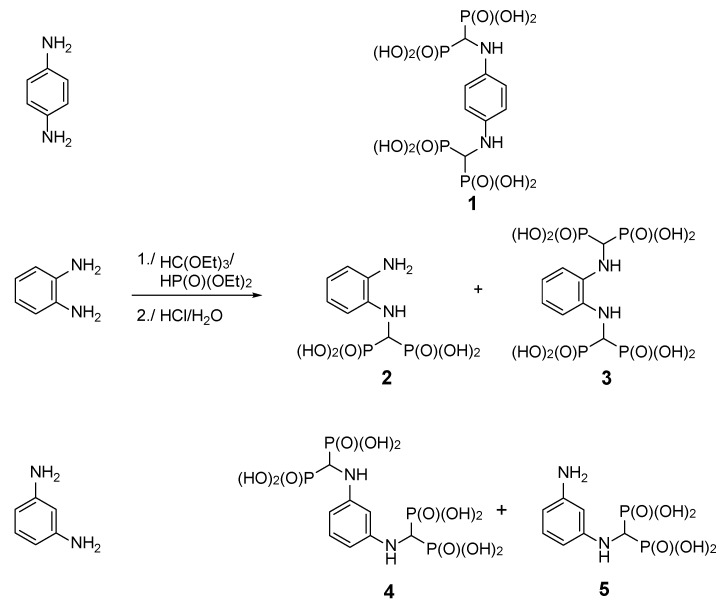
Three-component reaction of diaminobenzenes with triethyl orthoformate and diethyl phosphite.

**Figure 2:**
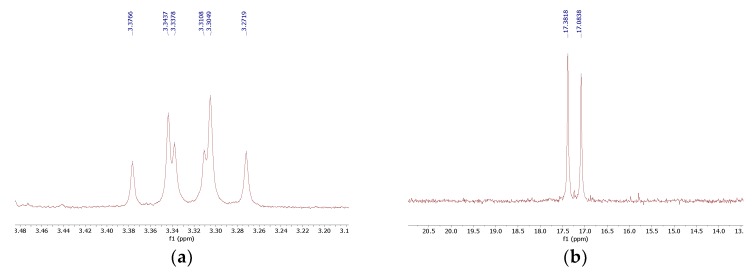
Fragments of ^1^H NMR (**a**) and ^31^P NMR (**b**) spectra that indicate the formation of the mono- and di-substituted products **2** and **3**.

The reaction of 1,4-diaminobenzene with triethyl orthoformate and diethyl phosphite (Figure 1) readily provided 1,4-di-substituted tetrabisphosphonic acid (compound **1**), which appeared to be relatively stable if stored at low temperature while protected against moisture. When 1,2-diaminobenzene was used, a mixture of products containing mono- and di-substituted compounds (compounds **2** and **3**, respectively) was obtained, with the mono-substituted produce the major one (as revealed from NMR and MS studies—Figure 2 and Supplementary Data), although we did not succeed in their isolation from the reaction mixture. 1,3-Diaminobenzene gave a complex mixture of inseparable products, most likely di- (predominating) and mono-substituted compounds (compounds **4** and **5**) accompanied by several side products. The formation of structurally variable side products is not surprising since it was frequently observed earlier in the three-component condensation of amines with trialkyl orthoformates and diethyl phosphites [15,16,17,18]. The efforts undertaken to separate and isolate the products of the reactions of 1,2- and 1,3-diaminobenzenes failed since the postreaction mixtures turned dark violet over time, and NMR studies revealed the intense decomposition of their components.

#### 2.1.2. Reactions of Diaminocyclohexanes

**Figure 3:**
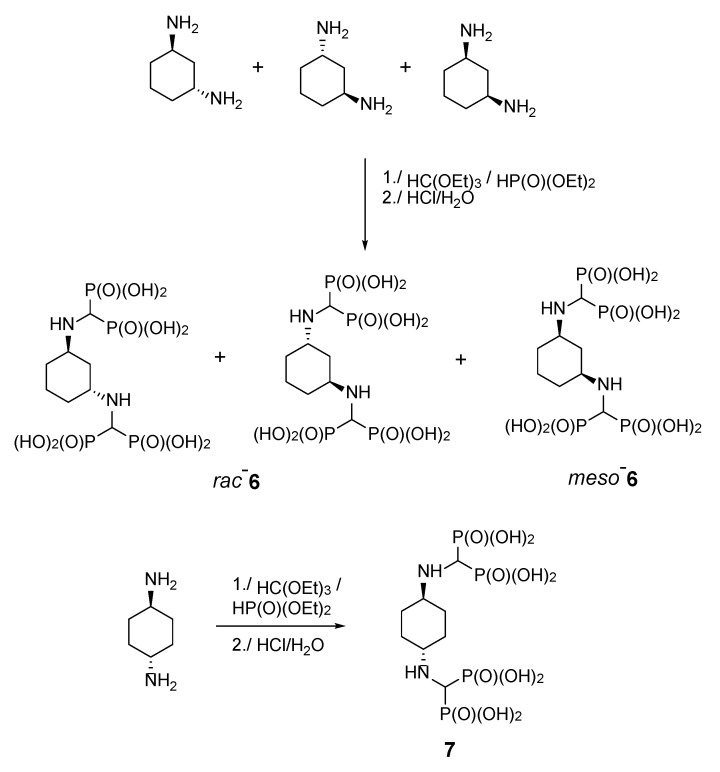
Products of the reactions of cyclohexane-1,3- and 1,4-diamines with triethyl orthoformate and diethyl phosphite.

Diaminocyclohexanes are valuable intermediates to produce dyestuffs, textile assistants, fungicides, pesticides, and pharmaceuticals and thus are commercially available in all stereoisomeric forms. We decided to undertake a more detailed study of the reactions in which the stereoisomers of diaminocyclohexanes were used as substrates. As expected, in the cases of cyclohexane-1,3-diamine and cyclohexane-1,4-diamine, two amino groups reacted (Figure 3). Thus, the reaction of a racemic mixture of *cis*,*trans*-cyclohexane-1,3-diamine provided compound **6**, seen as a doublet in the ^31^P NMR spectrum. This doublet is derived from the presence of a diastereomeric mixture composed of a *meso* isomer obtained from the *cis*-substrate and a mixture of enantiomers formed from the *trans*-substrate. The reaction of (±)-*trans*-cyclohexane-1,4-diamine gave *meso*-compound *trans*-**7**, as seen by the presence of a singlet in the ^31^P NMR spectrum.

**Figure 4:**
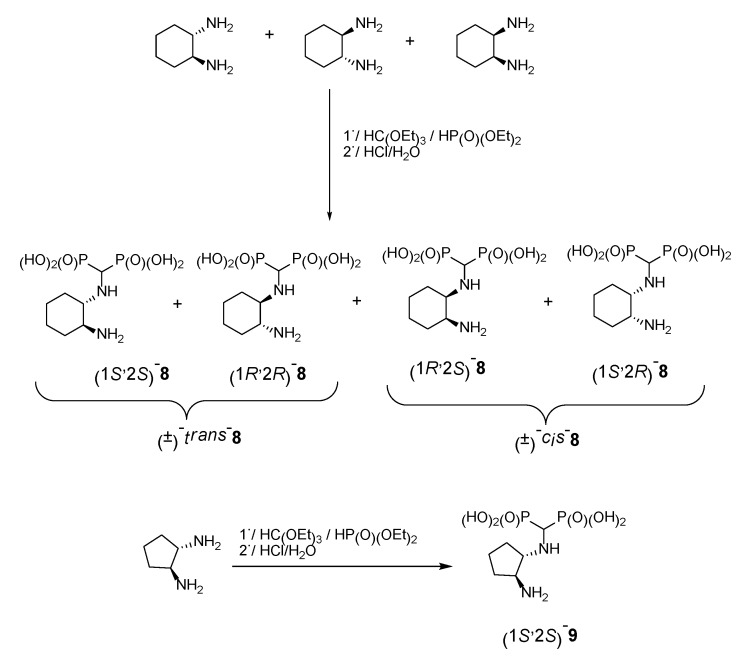
Products of reactions of various isomers of cyclohexane-1,2-diamines and (1*S*,2*S*)-cyclopentane-1,2-diamine.

In the case of cyclohexane-1,2-diamines, only one amino group reacted, yielding compound **8**, independent of the ratio of substrates being used. By using all the available stereoisomers of diaminocyclohexane, as well as their mixtures, we have proven that the absolute configuration is retained (Figure 4). Quite interestingly the differences in the ^1^H and ^13^C NMR spectra were small if not negligible (see Supplementary Data). In the ^31^P NMR, a slightly broadened doublet was observed for enantiomerically pure products—(1*S*,2*S*)- and (1*R*,2*R*)-cyclohexane-1-amino-2-aminomethylenebisphosphonic acids. This suggests that the phosphonic groups are magnetically nonequivalent. We speculate that this may result either from the conformation of compound **8** being frozen by the formation of intramolecular hydrogen bonding between the amino proton and phosphonic acid oxygen or from formation of strong hydrogen bonded dimers of this bisphosphonate. An identical result was obtained when using (1*S*,2*S*)-(+)-cyclopentane-1,2-diamine as a substrate (Figure 4).

**Figure 5:**
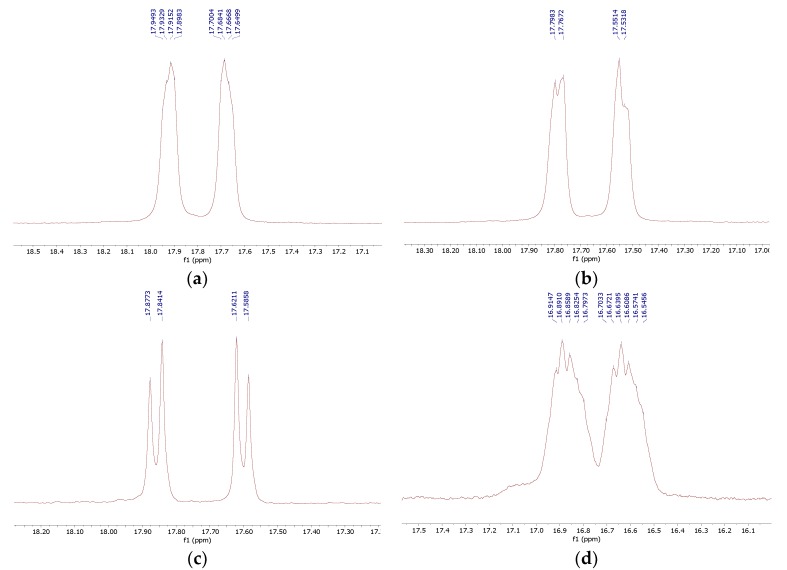
Phosphorus NMR spectra of isomers of (1*S*,2*S*)-**8** (**a**), (1*R*,2*R*)-**8** (**b**), (±)-*trans*-**8** (**c**), and *rac*-**8** (**d**).

The use of (±)-*trans*-1,2-diaminocyclohexane and an equimolar mixture of the *cis* and *trans* isomers as substrates resulted, as expected, in more complex ^31^P NMR spectra (Figure 5 and Supplementary Data), which reflects the growing number of stereoisomers.

#### 2.1.3. Reactions of Diaminocyclohexenes

**Figure 6:**
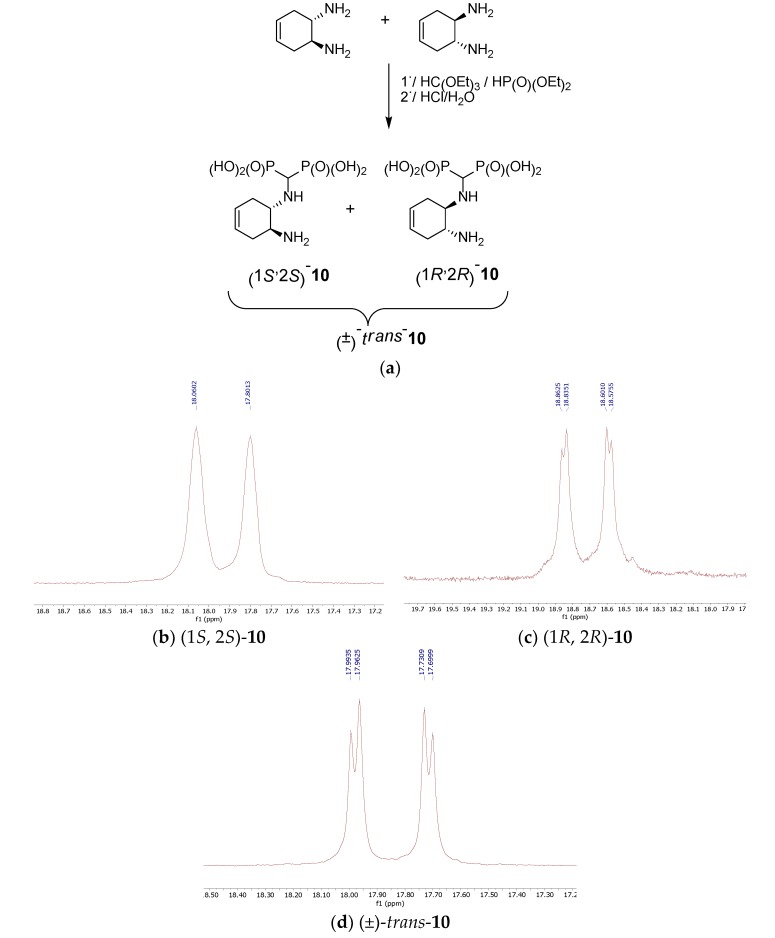
Isomers of *trans*-4-cyclohexene-1-amino-2-aminomethylenebisphosphonic acid (**a**) and representative ^31^P NMR spectra of these compounds (**b**–**d**).

The magnetic nonequivalence of the phosphonic groups is also pronounced when (1*S*,2*S*)- and (1*R*,2*R*)-4-cyclohexene-1,2-diamines and (±)-*trans*-4-cyclohexene-1,2-diamine were used as substrates (Figure 6). In this case, the ^31^P NMR spectrum of (±)-*trans*-4-cyclohexene-1-amino-2-amino-methylenebisphosphonic acid (compound *trans*-**10**) reveals two clearly separated doublets (Figure 6), whereas for the enantiomeric forms, broadened doublets are seen.

#### 2.1.4. Reactions of Piperazines

**Figure 7:**
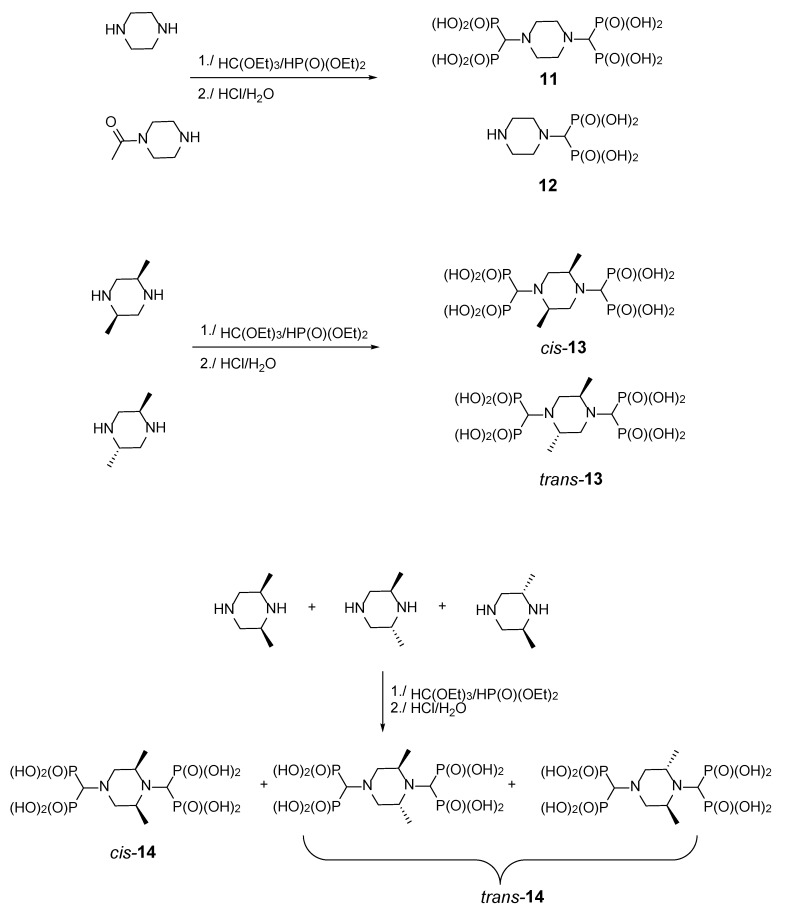
Products of the three-component reaction of piperazines, triethyl orthoformate, and diethyl phosphite.

The secondary amino groups of piperazine reacted smoothly, yielding piperaz-1,4-diylmethylenebisphosphonic acid **11**. The mono-substituted derivative **12** was obtained by using *N*-acetylpiperazine as a substrate (Figure 7). Stereoisomers of 2,5-dimethylpiperazine and 2,6-dimethylpiperazine were also used to provide bisphosphonates **13** and **14**, respectively (Figure 7).

**Figure 8:**
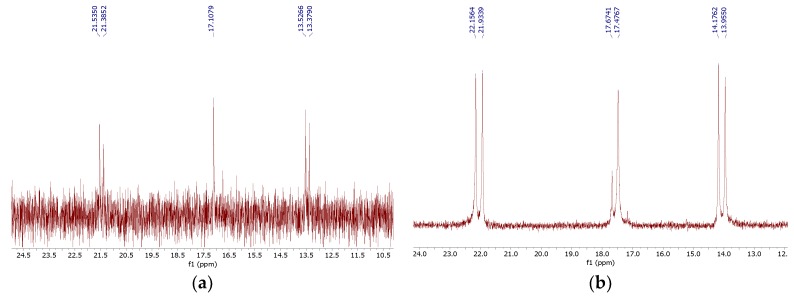
Phosphorus NMR spectra of 2,6-*cis*-dimethylpiperaz-1,4-diylmethylenebisphosphonic acid (*cis*-**14**) (**a**) and a mixture of the *cis* and *trans* isomers (*rac*-**14**) (**b**).

While all four phosphorus atoms in compound **11** are magnetically equivalent, which is demonstrated by the singlet in the ^31^P NMR spectrum, the spectra of compounds **13** and **14** are more complex. This is because the introduction of steric hindrance in proximity to the aminomethylenebisphosphonic substituent results in the nonequivalence of the phosphonic moieties, which is seen by the appearance of an AB spin system. Quite interestingly, the spectrum of compound *cis*-**14** is composed of both an AB system, derived from the substitution at position 1, and a singlet, resulting from the substitution at position 4 (Figure 8). In the spectrum of *rac*-**14**, only the substituents at position 4 differentiate the isomers to enable the determination that a non-equimolar mixture of the *cis* and *trans* isomers was obtained.

#### 2.1.5. Reactions of Aminoalkylidenediamines

**Figure 9:**
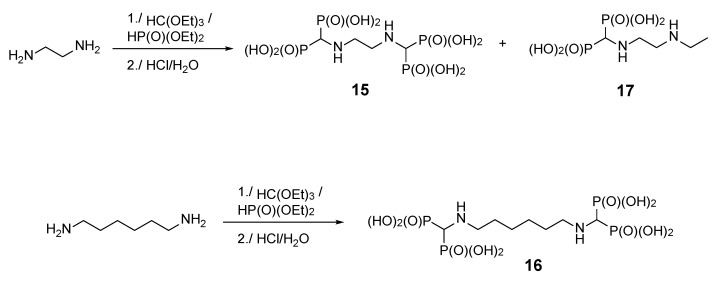
Products of the reactions of aminoalkylidenediamines.

To enlarge the set of compounds chosen for biological studies, it was additionally supplemented by compounds **15** and **16** (Figure 9). However, we were not able to obtain ethylene-di(aminomethylenebisphosphonic acid) **15** in a pure form since this reaction is accompanied by the ethylation of one amino group to yield compound **17**. Compounds **15** and **17** are difficult to separate, so we decided to perform the biological studies using the impure derivative.

### 2.2. In Vitro Evaluation

To screen for the potential antiosteoporotic activity of the bisphosphonates, their anti-proliferative activity towards in vitro cell cultures was determined. Because of the difficulty in isolating and culturing large numbers of osteoclasts, most studies devoted to the characterization of the pharmacological properties of bisphosphonates in vitro are performed on osteoclast surrogates, particularly macrophages [19,20]. Both RAW 264.7 macrophages and osteoclasts are derived from a hematopoietic lineage and are highly endocytic and capable of demineralizing bone particles [21,22]. They are well recognized for being sensitive to bisphosphonates, which most likely act by inducing apoptosis. The results summarized in Table 1 clearly show that nearly all the studied compounds effectively inhibit the proliferation of RAW 264.7 macrophages, exhibiting potency similar or slightly higher than incadronate and zoledronate, which are popular antiosteoporotic drugs. The only exceptions are compounds (1*S*,2*S*)-**9** and *rac*-**14**, which are an order of magnitude less active. The most active appeared to be compounds **1**, *rac*-**6**, and 11.

Since bisphosphonates accumulate in the bones, a strong anti-proliferative effect on bone metastatic and hematopoietic tumors can be expected, suggesting their possible clinical application as anti-cancer drugs. Therefore, we also tested the action of the synthesized compounds on the two classic cancer cell lines MCF-7 and PC-3. MCF-7 is a well-established model of breast cancer, which preferentially metastasizes to the bone, forming predominantly osteolytic lesions, whereas PC-3 is a human prostate cancer cell line used in prostate cancer research and drug development.

The in vitro anti-proliferative activities of the presented compounds against tumor cells vary. All the aliphatic aminomethylenebisphosphonic acids and bis[aminomethylene(bisphosphonic)] acids (**6**–**16**) showed good anti-proliferative activity towards the macrophage cell line RAW 264.7. The IC_50_ values ranged from 2 μM (compound **6**) to over 340 μM (**9**). The aliphatic phosphonates also showed good anti-proliferative activity towards the human breast cancer MCF-7 line (the IC_50_ values ranged from 13 μM to over 540 μM). With the exception of compounds *rac*-**6**, **11** and *rac*-**14**, all of the studied compounds inhibited the proliferation of MCF-7 in a manner equal to or stronger than the controls, incadronate and zoledronate, while being significantly less toxic than the popular anti-cancer agent cisplatin. The most active compounds were *trans*-**7**, *trans*-**13**, and **16**, which were still 2-4 times weaker than cisplatin. On the other hand, only compounds **1**, *trans*-**7**, (1*S*,2S)-**9**, *trans*-**13**, **15**, and **16** appeared to be equipotent with incadronate and zoledronate towards the PC-3 cell line.

All aliphatic bis- and tetraphosphonates showed poor anti-proliferative activity against the prostate cell line PC-3, except for compound *rac*-**14**. Quite interestingly compound *rac*-**14** was as potent as cisplatin while being only weakly active against the remaining cell lines, thus showing marked selectivity.

However, the aromatic bis[aminomethylene(bisphosphonic)] acid (**1)** showed a similar or higher activity than the reference bisphosphonates for the PC-3 line. Compounds **1**, **6** and **11** were the most active (Table 1) against the macrophage cell line RAW 264.7. Compound **1** showed a broad anti-proliferative activity towards all cells applied in the study. It inhibited the proliferation of MCF-7, RAW 264.7 and PC-3 cells with IC_50_ values ranging from approximately 5 μM (RAW 264.7) to 216 μM (PC-3). The IC_50_ value for PC-3 was one and half-fold lower than the corresponding value for zoledronic acid and in the same range as that for incadronic acid but worse than that of cisplatin. For the RAW 264.7 macrophages, compound **1** showed a nine times better anti-proliferative activity than zoledronate and a ten times higher activity than incadronate.

The derivatives **6**, **7**, **8**, **10**, **11**, **13**, **15**, and **16** exhibited a specificity of action against RAW 264.7 cells. Compounds **7**, **8**, **10**, **13**, **15** showed comparable results to zoledronic acid, while compounds **6** and **11** showed 20 and 6 times stronger anti-proliferative activities, respectively.

Similar studies of the anti-proliferative activity of this class of compounds towards the J774E, MC-7 and HL60 cell lines have been recently published [23]. The described set included compounds **1**, *trans*-**7**, and **16**. The results of these studies are more or less similar to those presented in this work, although with IC_50_ values that are significantly higher (including those of the controls). These studies also indicated that compound **1** was the most promising candidate for further studies.

Taking into account the possible clinical application compounds, **1**, *trans*-**7**, and **16** are the most interesting of all the compounds tested. They show broad nonspecific activity, higher than that found for incadronic acid, influencing the proliferation of RAW 264.7 macrophages and the PC-3 and MCF-7 cell lines. Compound **1** shows a broad nonspecific activity that is higher than that of incadronic acid. Compound **1** specifically and strongly influences the proliferation of RAW 264.7 macrophages and the PC-3 and MCF-7 lines. This is especially interesting in the context of anti-osteolytic treatment and the blocking of the interactions and mutual activation of osteoclasts and tumor metastatic cells in the bone microenvironment.

### 2.3. In vivo Evaluation of Compounds

Compounds **1**, *trans*-**7**, and **16** were the logical options for the treatment of sheep with induced osteoporosis. These animals were chosen due to the similarity of sheep to humans in weight, bone and joint structure, and bone regeneration mechanisms. Based on the previous literature report and our studies, compound **1** seemed to be the best choice. However, it is unstable in the solution prepared for application in sheep [24,25]. This is clearly visible from the solution turning violet, and ^31^P NMR spectra taken versus time indicate the decomposition of the carbon-to phosphorus bond to form phosphorous acid. Therefore, compound *trans*-**7** was chosen for in vivo studies, taking into consideration its stability and solubility in aqueous solutions. Because osteoporosis does not occur naturally in animals, it must be induced in sheep. It was induced by a separately described regimen based on ovariectomy followed by controlled methylprednisolone treatment with respect to bone metabolism and a suitable calcium/vitamin D-restricted diet [26]. The level of bone loss was determined by measuring the bone mineral density by means of quantitative computed tomography (QCT) and computer radiography at the PET-TK Laboratory, Medical Diagnostic Center in Kraków. The bone structural parameters were determined from iliac crest biopsy specimens using a diamond trephine.

**Figure 10:**
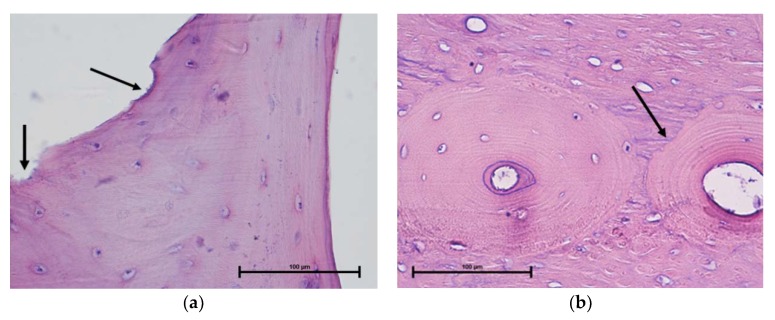
Erosion bays in tibia shown by arrows (**a**) and large osteon from shaft of femur, arrow indicates arrested osteogenesis (**b**). Magnification 400×.

After two months, the administration of bisphosphonate was started. An aqueous solution of the sodium salt of bisphosphonate *trans*-**7** (35 mg/sheep) was given to the animals every week using a probe directly inserted into the rumen. Ten doses of the drug were given to five out of seven sheep. One sheep was not medicated (negative control), whereas one was healthy (no osteoporosis was induced here—positive control). At the end, the animals were sacrificed, and bone samples were collected from the shaft, total hip, and radius of the femur and the proximal and distal tibial epiphysis for histology. This examination revealed the presence of small erosion bays, where no osteogenesis was observed, and large osteons characterized by arrested osteogenesis (Figure 10 and Supplementary Data). In some bone lamella, a dysfunctional course of collagen fibers was noted, which indicates an inability to finish the bone turnover process.

Summing up, a mild antiosteoporotic activity of compound *trans*-**7**, compared to incadronate (results not shown), has been found, and thus these results are considered unsatisfactory.

## 3. Experimental Section

### 3.1. General Information

All solvents and reagents were purchased from commercial suppliers, were of analytical grade and were used without further purification. Unless otherwise specified, solvents were removed with a rotary evaporator. The ^1^H-, ^31^P- and ^13^C-NMR spectroscopic experiments were performed on a Bruker Avance II Ultrashield Plus (Bruker, Rheinstetten, Germany) operating at 600.58 MHz (^1^H), 243.12 MHz (^31^P{^1^H}) and 151.016 MHz (^13^C), a Bruker Avance III 500 MHz (Bruker, Rheinstetten, Germany) operating at 500.14 MHz (^1^H), 202.46 MHz (^31^P) and 125.77 MHz (^13^C), a Bruker Advance TM DRX operating at 121.51 MHz (^31^P), and a JEOL JNM-ECZ 400S Research FT NMR Spectrometer (JEOL Ltd., Tokyo, Japan) operating at 399.78 MHz (^1^H), 161.83 MHz (^31^P{^1^H}) and 100.53 (^13^C). Measurements were made in solutions of D_2_O + NaOD at 300 K, and all solvents were supplied by ARMAR AG (Dottingen, Switzerland). Chemical shifts are reported in ppm relative to TMS (tetramethylsilane) and 85% H_3_PO_4_, used as external standards, and coupling constants are reported in Hz. Melting points were determined on an SRS Melting Point Apparatus OptiMelt MPA 100 (Stanford Research Systems, Sunnyvale, CA, USA) and are reported uncorrected. The purity of all test compounds is higher than 95% by ^1^H NMR and LC-MS. Mass spectra were recorded at the Faculty of Chemistry, Wrocław University of Science and Technology using a Waters LCT Premier XE mass spectrometer (method of electrospray ionization, ESI) (Waters, Milford, MA, USA).

### 3.2. Synthetic Procedures

#### 3.2.1. General Procedure for the Synthesis of Aliphatic Bisaminomethylenebisphosphonates (6, 8–11, 16): Method A

A mixture of an aliphatic diamine (0.03 mol) and the appropriate amounts of diethyl phosphite (0.126 mol, 16.32 mL) and triethyl orthoformate (0.063 mol, 4.4 mL) was heated with stirring at an elevated temperature (130 °C) for 15 h (overnight) on the heating plate of a Radleys Carousel. Then, the volatile components of the reaction mixture were evaporated, and the residue was dissolved in chloroform (200 mL). The organic layer was extracted three times with water (3 × 150 mL), and the inorganic layer was evaporated under reduced pressure. The crude esters were then subjected to hydrolysis.

#### 3.2.2. General Procedure for the Synthesis of Bisaminomethylenebisphosphonates (1–7,trans-8, 11–14): Method B

Diamine (0.03 mol) and the appropriate amounts of diethyl phosphite (0.126 mol, 16.32 mL) and triethyl orthoformate (0.063 mol, 4.4 mL) were heated and simultaneously stirred at a temperature of ~130 °C on a heating plate (125 °C in the reaction medium) of a Radleys Carousel apparatus overnight (15 h). The mixture was cooled, and the volatile components were removed using a rotary evaporator. The resulting mixture was dissolved in ethyl acetate (100 mL) and purified by washing with water (100 mL), saturated sodium chloride solution (100 mL) and again with water (100 mL). The solution was dried over anhyd MgSO4, and the solvent was evaporated under vacuum. The crude esters were then subjected to hydrolysis.

#### 3.2.3. Hydrolysis—General Procedure

The obtained ester (0.030 mol) was refluxed for 15 h (overnight) in 40 mL 6 M aqueous hydrochloric acid solution. After cooling, the volatile components were removed using a rotary evaporator, and the resulting oil was dissolved in a minimal amount of water (40–50 mL), decolored with activated charcoal and purified by crystallization from a water–ethanol mixture (80/20 *v*/*v*). The impure product was mixed with water for a few days until the dissolution of impurities was observed and then was filtered and washed with distilled water and dried in vacuo.

Phenylene-1,4-di(aminomethylenebisphosphonic) acid (**1**) [14,23] was obtained as a purple solid; yield: 62% (method B); mp 342–344 °C.

Cyclohexane-1,3-di(aminomethylenebisphosphonic) acid (**6**) [27] was obtained as a white solid; yields: 11% (method A) and 51% (method B); mp 269–270 °C.

*trans*-Cyclohexane-1,4-di(aminomethylenebisphosphonic) acid (*trans*-**7**) [14,23,27] was obtained as a white solid; yield: 52% (method B); mp 243 °C.

(1*S*,2*S*)(+)-4-Cyclohexane-1-amino-2-aminomethylenebisphosphonic acid [(1*S*,2*S*)-**8**] was obtained as a white solid; yield: 19% (method A); mp 245–246 °C; ^31^P-NMR (243.12 MHz, D_2_O + NaOD, ppm) δ = 17.79 (broad AB spin system); ^1^H-NMR (600.58 MHz, D_2_O + NaOD, ppm) δ = 0.74–0.85 (m, 1H), 0.94–1.14 (m, 3H), 1.43–1.56 (m, 2H), 1.61–1.68 (m, 1H), 1.93–2.03 (m, 1H), 2.17–2.27 (m, 1H), 2.40–2.49 (m, 1H), 2.68 (t_br_, 1H, *J* = 19.9 Hz, CHP_2_); ^13^C-NMR (151.02 MHz, D_2_O + NaOD, ppm) δ = 23.88, 24.22, 29.72, 31.87, 54.76, 55.47 (d, *J* = 115.83 Hz, CHP), 56.31 (d, *J* = 115.57 Hz, CHP), 62.30; HRMS (TOF MS ESI); [M − H]^−^ Calcd for C_7_H_18_N_2_O_6_P_2_: 287.0562; found: 287.0511.

(1*R*,2*R*)(−)-4-Cyclohexane-1-amino-2-aminomethylenebisphosphonic acid [(1*R*,2*R*)-**8**] was obtained as a white solid; yield: 13% (method A); mp 238–239 °C; ^31^P-NMR (243.12 MHz, D_2_O + NaOD, ppm) δ = 17.61 (broad AB spin system); ^1^H-NMR (600.58 MHz, D_2_O + NaOD ppm) δ = 0.75–0.88 (m, 1H), 0.94–1.18 (m, 3H), 1.44–1.60 (m, 2H), 1.66–1.71 (m, 1H), 1.92–2.05 (m, 1H), 2.12–2.30 (m, 1H), 2.41–2.52 (m, 1H), 2.69 (t, 1H, *J* = 23.24 Hz, CHP_2_); ^13^C-NMR (151.02 MHz, D_2_O + NaOD, ppm) δ = 24.44, 24.67, 30.42, 33.55, 55.32, 56.27 (d, *J* = 123.20 Hz, CHP), 57.25 (d, *J* = 123.14 Hz, CHP), 63.06; HRMS (TOF MS ESI); [M − H]^−^ Calcd for C_7_H_18_N_2_O_6_P_2_: 287.0562; found: 287.0548 and 575.0829 [2M − H]^−^.

(±)-(*trans*)-Cyclohexane-1-amino-2-aminomethylenebisphosphonic acid [(*trans*)-**8**] was obtained as a white solid; yields: 10% (method A) and 64% (method B); mp 235–236 °C; ^31^P-NMR (243.12 MHz, D_2_O + NaOD, ppm) δ= 17.73 (AB spin system, *J* = 17.73 Hz); ^1^H-NMR (600.58 MHz, D_2_O + NaOD, ppm) δ= 0.82 (q_br_, 1H, *J* = 11.50 Hz), 1.01 (q, 1H *J* = 11.65 Hz), 1.08–1.12 (m, 2H), 1.49–1.54 (m, 2H), 1.66 (d, 1H, *J* = 12.60 Hz), 1.99 (d, 1H, *J* = 12.75 Hz), 2.21–2.26 (m, 1H), 2.44–2.48 (m, 1H), 2.67 (d, 0.5H, *J* = 16.88 Hz, CHP); 2.71 (d, 0.5H, J = 17.17 Hz, CHP); ^13^C-NMR (151.02 MHz, D_2_O + NaOD, ppm) δ = 24.51, 24.73, 30.57, 33.68, 55.43, 56.25 (d, *J* = 124.58 Hz, CHP), 57.13 (d, *J* = 124.50 Hz, CHP), 57.54; HRMS (TOF MS ESI); [M − H]^−^ Calcd for C_7_H_18_N_2_O_6_P_2_: 287.0562; found: 287.0511.

Racemic cyclohexane-1-amino-2-aminomethylenebisphosphonic acid [(*rac*)-**8**] [27] was obtained as a white solid; yield: 18% (method A); mp 235–236 °C

(1*S*,2*S*)-Cyclopentane-1-amino-2-aminomethylenebisphosphonic acid [(*1S*,*2S*)-**9**] was obtained as a white solid; yield: 17% (method A); mp 227–228 °C; ^31^P-NMR (121.49 MHz, D_2_O + NaOD, ppm): δ = 18.33 (broad AB spin system); ^1^H-NMR (500.14 MHz, D_2_O + NaOD, ppm): δ = 1.14–1.25 (m, 2H), 1.44–1.49 (p, 2H, *J* = 7.34 Hz), 1.72–1.79 (m, 1H), 1.85–1.92 (m, 1H), 2.62 (t, 1H, *J* = 17.33 Hz, CHP_2_), 2.80 (q, 1H, *J* = 6.50 Hz, CHN), 3.10 (q, 1H, *J* = 6.59 Hz, CHN); ^13^C-NMR (125.77 MHz, D_2_O + NaOD, ppm) δ = 20.84, 30.29, 32.70, 34.27 (t, *J* = 121.64 Hz, CHP_2_), 57.45 (CHN), 66.28 (CHN); HRMS (TOF MS ESI); [M − H]^−^ Calcd for C_6_H_16_N_2_O_6_P_2_: 273.0405 found: 273.0296.

(1*S*,2*S*)(+)-4-Cyclohexene-1-amino-2-aminomethylenebisphosphonic acid [(1*S*,2*S*)-**10**] was obtained as a light brown solid; yield: 21% (method A); mp 220–221 °C; ^31^P-NMR (243.12 MHz, D_2_O + NaOD, ppm) δ =17.80 and 18.06; ^1^H-NMR (600.58 MHz, D_2_O + NaOD, ppm) δ = 1.61−1.77 (m, 2H), 2.08−2.32 (m, 2H), 2.57−2.70 (m, 2H), 2.787−2.89 (m, 1H, CHP_2_), 5.37−5.53 (m, 2H, CH =CH); ^13^C-NMR (151.02 MHz, D_2_O + NaOD, ppm) δ = 29.12, 31.82, 50.40, 56.04 (d, *J* = 124.23 Hz, CHP), 56.91 (d, *J* = 124.13 Hz, CHP), 124.89, 125.37; HRMS (TOF MS ESI); [M + H]^+^ Calcd for C_7_H_16_N_2_O_6_P_2_: 287.0562; found: 287.0572.

(1*R*,2*R*)-4-Cyclohexene-1-amino-2-aminomethylenebisphosphonic acid [(1*R*,2*R*)-**10**] was obtained as a light brown solid; yield: 12% (method A); mp 235−236 °C; ^31^P-NMR (243.12 MHz, D_2_O + NaOD, ppm) δ = 18.72 (AB spin system, *J* = 7.81 Hz); ^1^H-NMR (600.58 MHz, D_2_O + NaOD, ppm) δ = 1.34−1.54 (m, 2H), 1.88 (d, 1H, *J* = 17.70 Hz), 2.00 (d, 1H, *J* = 18.22 Hz), 2.30−2.48 (m, 2H), 2.53−2.68 (m, 1H, CHP_2_), 5.15−5.28 (m, 2H, CH = CH); ^13^C-NMR (151.02 MHz, D_2_O + NaOD, ppm) δ= 28.61, 31.55, 49.74, 55.50 (t, *J* = 196.3 Hz, CHP_2_), 56.62, 124.98, 1215.59; HRMS (TOF MS ESI); [M − H]^−^ Calcd for C_7_H_16_N_2_O_6_P_2_: 285.0405; found: 285.0405.

(±)-*trans*-4-Cyclohexene-1-amino-2-aminomethylenebisphosphonic acid [(*trans*)-**10**] was obtained as a cream solid; yield: 18% (method A); mp 243–244 °C; ^31^P-NMR (243.12 MHz, D_2_O + NaOD, ppm) δ = 17.85 (AB spin system, *J* = 7.56 Hz); ^1^H-NMR (600.58 MHz, D_2_O + NaOD, ppm) δ = 1.70–1.74 (m, 2H), 2.18 (d, 1H, *J* = 17.56 Hz), 2.32 (d, 1H, *J* = 17.85 Hz), 2.65–2.72 (m, 2H), 2.87–2.91 (m, 1H, CHP_2_), 5.49–5.52 (m, 2H, CH =CH); ^13^C-NMR (151.02 MHz, D_2_O + NaOD, ppm) δ = 29.16, 31.83, 50,35, 55.79 (d, *J* = 127.32 Hz CHP), 56.67 (d, *J* = 126.71 Hz CHP), 57.31(s_br_), 124.99, 125.67; HRMS (TOF MS ESI); [M − H]^−^ Calcd for C_7_H_16_N_2_O_6_P_2_: 285.0405; found: 285.0418.

Piperaz-1,4-diylmethylenebisphosphonic acid (**11**) [14] was obtained as a white solid; yield: 25% (method A), 42% (method B); mp 270–271 °C; ^13^C-NMR (151.02 MHz, D_2_O + NaOD, ppm) δ =51.32, 66.17 (t, *J* = 122.92 Hz, CHP_2_); HRMS (TOF MS ESI); [M − H]^−^ Calcd for C_6_H_18_N_2_O_12_P_4_: 432.9732; found: 432. 9713.

Piperaz-1-ylmethylenebisphosphonic acid (**12**) was obtained as a white solid; yield: 52% (method B); mp 251–252 °C; ^31^P-NMR (161.83 MHz, D_2_O + NaOD, ppm) δ = 17.63; ^1^H-NMR (399.78 MHz, D_2_O + NaOD, ppm) δ = 2.24–2.31 (m, 4H), 2.33 (t, 1H, *J* = 21.75 Hz, CHP_2_), 2.50–2.60 (m, 4H); ^13^C-NMR (100.53 MHz, D_2_O + NaOD, ppm) δ = 45.01, 51.16, 66.11 (d, *J* = 114.4 Hz, CHP), 67.36 (d, *J* = 119.4 Hz, CHP); HRMS (TOF MS ESI); [M − H]^−^ Calcd for C_5_H_14_N_2_O_6_P_2_: 259.0249; found: 259.0243.

2,5-*trans*-Dimethylpiperaz-1,4-diylmethylenebisphosphonic acid [(*trans*)-**13**] was obtained as a white solid; yield: 32% (method B); mp 244–245 °C; ^31^P-NMR (243.12 MHz, D_2_O + NaOD, ppm) δ = 13.96 (AB spin system, *J* = 18.60 Hz); ^1^H-NMR (600.58 MHz, D_2_O + NaOD, ppm) δ = 0.95 and 0.96 (s, 6H), 2.88 (t, 2H, *J* = 11.23 Hz), 3.15 (t, 2H, *J* = 23.28 Hz, CHP_2_), 3.29–3.39 (m, 2H), 3.47 (d, 2H, *J* = 11.62 Hz); ^13^C-NMR (151.02 MHz, D_2_O + NaOD, ppm) δ = 16.19, 55.60, 55.67, 56.13, 58.76 (t, *J* = 124.2 Hz, CHP_2_); HRMS (TOF MS ESI); [M − H]^−^ Calcd for C_8_H_22_N_2_O_12_P_4_: 461.0045; found: 461.0029.

2,5-*cis*-Dimethylpiperaz-1,4-diylmethylenebisphosphonic acid [(*cis*)-**13**] was obtained as a white solid; yield: 32% (method B); mp 237–239 °C; ^31^P-NMR (202.46 MHz, D_2_O + NaOD, ppm) δ = 13.89 (ABX spin system, *J*_P-H_ = 19.73 Hz); ^1^H-NMR (500.14 MHz, D_2_O + NaOD, ppm) δ = 0.99 and 1.00 (s, 6H), 2.91 (t, 2H, *J* = 11.87 Hz, CHP_2_), 3.16 (d, 2H, *J* = 19.34 Hz), 3.21 (d, 2H, *J* = 19.28 Hz), 3.32–3.42 (m, 2H), 3.51 (d, 2H, *J* = 10.66 Hz); ^13^C-NMR (125.77 MHz, D_2_O + NaOD, ppm) δ = 16.10, 55.62, 55.73, 56.16, 56.20, 58.30 (d, *J* = 115.8 Hz, CHP), 59.29 (d, *J* = 115.7 Hz, CHP); HRMS (TOF MS ESI); [M − H]^−^ Calcd for C_8_H_22_N_2_O_12_P_4_: 461.0045; found: 461.0027.

Racemic 2,6-Dimethylpiperaz-1,4-diylmethylenebisphosphonic acid [(*rac*)-**14**] was obtained as a white solid; mixture of trans/cis isomers (4:2); yield: 37% (method B); mp 242–243 °C; ^31^P-NMR (161.83 MHz, D_2_O + NaOD, ppm) δ = 17.90 (AB spin system, *J* = 35.90 Hz, both isomers); 17.48 (s, major isomer), 17.67 (s, minor isomer); ^1^H-NMR (399.78 MHz, D_2_O + NaOD, ppm) δ = 0.56–0.70 (m, 4H), 0.82–0.93 (m, 2H), 2.10–2.29 (m, 2H, CHP_2_), 2.33–2.70 (m, 5H), 3.50–3.70 (m, 1H); ^13^C-NMR (100.53 MHz, D_2_O + NaOD, ppm) δ = 16.17, 16.19, 16.23, 16.25, 18.37, 18.42, 50.00, 50.12, 50.26, 56.70, 57.36, 58.46, 60.98, 61.04, 63.83 (t, *J* = 131 Hz, CHP_2_); HRMS (TOF MS ESI); [M − H]^−^ Calcd for C_8_H_22_N_2_O_12_P_4_: 461.0045; found: 461.0043.

2,6-*cis*-Dimethylpiperaz-1,4-diylmethylenebisphosphonic acid [(*cis*)-**14**] was obtained as a white solid; yield: 27% (method B); mp 240–242 °C; ^31^P-NMR (243.16 MHz, D_2_O + NaOD, ppm) δ = 17.11 (s), 17.39 (AB system, *J* = 36.15 Hz); ^1^H-NMR (600.58 MHz, D_2_O + NaOD, ppm) δ = 0.61–0.67 (m, 3H), 0.87–0.96 (m, 3H), 2.18 (t, 1H, *J* = 12.07 Hz, CHP_2_), 2.25 (t, 1H, *J* = 11.15 Hz, CHP_2_), 2.39–2.49 (m, 2H), 2.52–2.68 (m, 2H), 3.55–3.64 (m, 2H); HRMS (TOF MS ESI); [M − H]^−^ Calcd for C_8_H_22_N_2_O_12_P_4_: 461.0045; found: 461.0055.

Hexylene-di(aminomethylenebisphosphonic acid (**16**) [23] was obtained as a white solid; yield: 37% (method A); mp 211–212 °C.

#### 3.2.4. Cell Cultures

Mycoplasma-free MCF-7, PC-3, and RAW 264.7 cell lines were purchased from the European Collection of Authenticated Cell Cultures (ECACC) and maintained at the Institute of Immunology and Experimental Therapy (IIET), Wrocław, Poland. MCF-7 cell line was cultured in Eagle medium (Life Technologies, Scotland, UK) supplemented with 10% (*v*/*v*) FBS (Fetal Bovine Serum), 2 mM L-glutamine, 1% NEAA (Non-Essential Amino Acid), 0.01 mg/mL insulin (all Sigma-Aldrich, Poland). The PC-3 cell line was cultured in RPMI-1640 medium (Life Technologies, Scotland) supplemented with 10% (*v*/*v*) FBS and 2 mM L-glutamine. The RAW 264.7 cell line was cultured in DMEM (Dulbecco’s Modified Eagle Medium, Life Technologies, Scotland) supplemented with 10% (*v*/*v*) FBS and 2 mM L-glutamine. All culture media were additionally supplemented with 100 µg/mL streptomycin and 100 U/mL penicillin. Cell lines were cultured during all experiments in a humid atmosphere at 37 °C and 5% CO_2_ and passaged twice a week using EDTA-trypsin (pH 8; IIET, Wrocław, Poland) solution as a detachment agent.

#### 3.2.5. SRB Anti-Proliferative Assay

24 h before adding the tested compounds, the cells were seeded on 96-well plates (Sarstedt, Germany) in appropriate culture medium with 0.75 × 10^5^ cells/mL for MCF-7, 10^5^ cells/mL for PC-3, and 0.1 × 10^5^ cells/mL for RAW 264.7. Cells were treated with each compound in at least four concentrations in the range of 1000 µM^−1^ µM for 72 h. 0.2 M NaOH, used as a stock solution solvent, was tested for anti-proliferative activity, and it did not affect the cell proliferation at 1 mM, the highest concentration used in the compound solutions.

Experiments were carried out according to the described procedure [28], with minor modifications for all adherent cells. Briefly, cells were fixed with 50 µL/well of 50% (*w/v*) trichloroacetic acid (Avantor Performance Materials, Gliwice, Poland). After 1h incubation, the plates were washed several times with tap water, and 50 µL of a 0.1% (*w*/*v*) solution of sulforhodamine B (Sigma-Aldrich, Germany) in 1% (*v*/*v*) acetic acid (Avantor Performance Materials, Gliwice, Poland) was added to each well. After 30 min of incubation at room temperature, the unbound dye was washed out with 1% (*v/v*) acetic acid, and the bound dye was solubilized with 10 mM unbuffered TRIS (tris(hydroxyethyl)aminomethane, Avantor Performance Materials, Gliwice, Poland) solution. The entire procedure was performed using a Biotek EL-406 washing station (BioTek Instruments, Winooski, VT, USA). The absorbance was next read using a Biotek Hybrid H4 reader (BioTek Instruments) at 540 nm.

The compounds at each concentration were tested in triplicate in a single experiment, and each experiment was repeated at least three times independently. The results are presented as the mean IC_50_ ± standard deviation (SD) and calculated using the Prolab-3 system based on Cheburator 0.4 software [29].

### 3.3. In vivo Studies

#### 3.3.1. Studies on Sheep

Compound *trans*-**7** was tested on a group of seven female “merino” sheep, aged 5–6 years and weighing 45–60 kg. Sheep were purchased from Agricultural Experimental Station of Wrocław University of Environmental and Life Sciences in Pawłowice, Poland. The group contained one sheep on which no procedures were performed (negative control), one sheep after ovariectomy (positive control) and five sheep after ovariectomy and glucocorticosteroid therapy. The animals were kept in small boxes of a stable with a size of 10 m^2^, three in each. This was done to restrict their physical activity and access to sunlight. The sheep were fed twice a day with hay and provided with unrestricted access to water. All animals were weighed, and blood samples for the biochemical, morphological, hormonal, and bone turnover markers were collected each month of the experiment. Two months after the induction of osteoporosis, the administration of the tested bisphosphonate started. Thus, 35 mg of *trans*-**7** was dissolved in 35 mL of deionized water and pH was adjusted to 7.24 by addition 3M NaOH (ca. 1.3 mL). This solution (35 mg of bisphosphonic acid/sheep, which is equal to 0.58–0.78 mg/kg) was given to the animals every week using a probe inserted directly into the rumen. Ten doses of bisphosphonate were given to each sheep. Finally, sheep were maintained at normal diet for additional 3,5 months. At the end, the animals were sacrificed, and bone samples from the femur and tibia were collected for histology.

All studies were conducted in accordance with the Wrocław 2nd Local Ethics Committee for Animal Experiments (resolution no 85/2009 of 27.02.2009) and was reviewed at and the study protocol was approved by this ethics committee.

#### 3.3.2. Histological Studies

The bone samples underwent conservation in 4% formalin solution and were decalcified by successive bathing in 10% sodium versenate, distilled water, a mixture of formic and citric acids, and distilled water. Then, they were dehydrated and kept in paraffin. The bone segments were sectioned with a microtome to 6–7 μm slides for histomorphometric analysis. Before the analysis, the samples were fixed for 3 h in a 3.5% solution of glutaraldehyde in phosphate buffer (0.1 M, pH between 7.2 and 7.4), washed thoroughly with buffer and then fixed with a solution containing 1.0% osmium tetroxide in the same buffer. After drying (ethanol-acetone), the samples were submerged in Epon 812.

The bone slides were stained by the Delafield method with hematoxylin and eosin and analyzed by transmission microscopy (Tesla BS-500).

## 4. Conclusions

A three-component reaction between 1,4-diamines, triethyl orthoformate, and diethyl phosphite resulted in introduction of two aminomethylenebisphosphonate moieties into the molecule. When 1,2-diamines were used as the substrates, the reaction surprisingly provided mono-substituted aminomethylenebisphosphonates despite the ratio of the substrates used most likely, as a result of steric hindrance. Moreover, stability of the mono-substituted products is enhanced by the formation of hydrogen bonding between the phosphonate oxygen and the hydrogen of the adjacent amino group. When 1,3-diamines were used as substrates mixtures of products of mono- and di-substitution had been obtained. ^31^P NMR spectra revealed interesting non-equivalency of phosphorus atoms of the obtained compounds.

The compounds obtained revealed diverse, albeit promising, activity towards PC-3, MCF-7, and RAW 264.7 cells. Three of them, namely compounds **1**, *trans*-**7**, and **16**, showed broad nonspecific activity, higher than that of incadronic acid (control), influencing the proliferation of all cell lines and thus seem to be of particular interest in the context of anti-osteolytic therapy.

Tetraphosphonate *trans*-**7** was chosen for the medication of sheep with induced osteoporosis, but the result of this therapy was unsatisfactory. This shows that screening done based on the anti-proliferative activity of bisphosphonates towards macrophage-like cell lines is not fully predictive if considering antiosteoporetic activity. On the other hand, medication with bisphosphonates is a long process, while the results of this study were obtained after just two and a half of months of medication with some improvement of the bone status of the sheep observed after next three and a half of months. Thus, this might be seen as a promising result.

## Figures and Tables

**Table 1:** Structures and in vitro anti-proliferative activities of bis[aminomethylenebis(phosphonic)] acids against RAW 264.7 mouse macrophages, PC-3 human prostate cancer cells and MCF-7 human breast cancer cells.

Compound No.	Structure	IC_50_ [μM] ^a^		
		RAW 264.7	PC-3	MCF-7
1	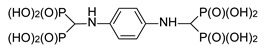	4.9 ± 2.3	215.8 ± 10.9	79.1 ± 25.6
*rac*-6	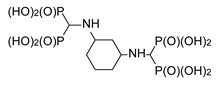	2.1 ± 5.3	496.9 ± 166.3	UN ^b^
*trans*-7	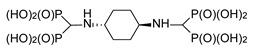	23.3 ± 7.1	284.5 ± 19.8	13.0 ± 5.1
(1*S*,2*S*)-8	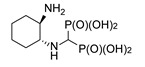	30.7 ± 2.0	616.5 ± 172.5	53.7 ± 10.9
*trans*-8	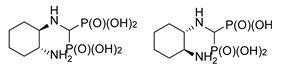	18.5 ± 8.3	854.8 ± 96.6	73.8 ± 22.0
*rac*-8	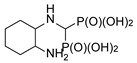	19.9 ± 5.4	794.8 ± 18.7	83.2 ± 10.8
(1*S*,2*S*)-9	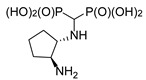	341.8 ± 56.8	212.3 ± 5.7	124.6 ± 4.0
(1*S*, 2*S*)-10	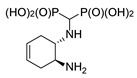	16.3 ± 11.1	492.1 ± 173.5	55.4 ± 19.2
(1*R*, 2*R*)-10	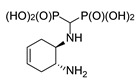	16.6 ± 4.7	640.6 ± 162.3	66.4 ± 25.3
*trans*-10	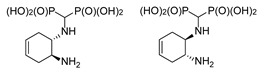	26.9 ± 8.2	668.1 ± 158.8	213.6 ± 85.9
11	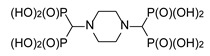	7.0 ± 12.6	UN ^b^	UN ^b^
*trans*-13	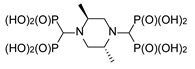	40.8 ± 9.7	228.2 ± 112.9	23.6 ± 86.0
*rac*-14	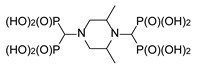	259.7 ± 28.9	6.1 ± 2.2	542.1 ± 40.4
15 ^c^	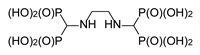	12.2 ± 4.0	169.6 ± 78.5	60.9 ± 18.6
16	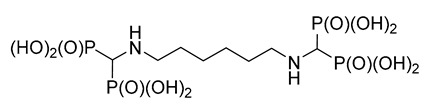	20.8 ± 1.6	293.0 ± 91.4	23.2 ± 6.2
Incadronic acid	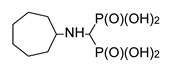	48.4 ± 12.6	228.6 ± 64.5	186.8 ± 24.7
Zoledronic acid	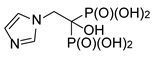	42.2 ± 8.4	146 ± 67.4	115.3 ± 87.6
cisplatin	(H_2_N)_2_PtCl_2_	0.93 ± 0.4	9.83 ± 1.7	6.37 ± 0.8

^a^ IC_50_ values were determined at concentrations in the range of 1–1000 μg/mL. Values are mean ± standard deviation from at least three experiments performed in triplicates. ^b^ Compound was inactive in the concentration range tested. ^c^ Impure compound (see text).

## Supplementary Materials

The following are available online. The datasets generated during the current study are available from the corresponding author on reasonable request. All data generated or analyzed during this study are included in this published article Spectroscopic data for compounds (^1^H NMR, ^31^P NMR, ^13^C NMR and MS) for compounds: **1**, **6**, **7**, (1*S*, 2*S*)-**8**, (1*R*, 2*R*)-**8**, *trans*-**8**, *rac*-**8**, (1*S*, 2*S*)-**9**, (1*S*, 2*S*)-**10**, (1*R*, 2*R*)-**10**, *trans*-**10**, **11**, **12**, *trans*-**13**, *cis*-**13**, *cis*-**14**, *rac*-**14**, **16**, incadronate and mixture of **15** &**17** (only ^1^H NMR and ^31^P NMR); Figure S1. Histology (Delafield staining); Figure S2. Examples of erosion bays; Figure S3. Dysfunctional course of collagen fibres of tibia (magnification 600×); Figure S4. Bone repair (magnification 400×).
