# Supplementary material for: Three-Component Reaction of Diamines with Triethyl Orthoformate and Diethyl Phosphite and Anti-Proliferative and Antiosteoporotic Activities of the Products
Source: Molecules. 2020 Mar 20;25(6):1424. doi: 10.3390/molecules25061424 (PMC7144383; doi:10.3390/molecules25061424)
Supplement: Supplementary file 1 [file molecules-25-01424-s001.pdf]

Three-component reaction of diamines with triethyl orthoformate and diethyl phosphite and antiproliferative and antiosteoporotic activities of the products

Patrycja Petruczynik[1], Paweł Kafarski[1], Mateusz Psurski[2], Joanna Wietrzyk[2], Zdzisław Kiełbowicz[3], Jan Kuryszko[4] and Ewa Chmielewska[1]*

[1]Department of Bioorganic Chemistry, Faculty of Chemistry, Wrocław University of Science and Technology, Wybrzeże Wyspiańskiego 27, 50-370 Wrocław, Poland; ewa.chmielewska@pwr.edu.pl

[2] Laboratory of Experimental Anticancer Therapy, Department Of Experimental Oncology, Ludwik Hirszfeld Institute of Immunology and Experimental TherapyPolish Academy of Sciences, Rudolfa Weigla 12, 53-114 Wrocław, Poland;

[3] Department of Surgery, The Faculty of Veterinary Medicine, Wrocław University of Environmental and Life Sciences, Norwida 31, 50-375 Wrocław, Poland

[4] Division of Histology and Embryology, Department of Animal Physiology and Biostructure, The Faculty of Veterinary Medicine, Wrocław University of Environmental and Life Sciences, Norwida 31, 50-375 Wrocław, Poland

* Correspondence: ewa.chmielewska@pwr.edu.pl ; Tel.: +48-71 320 29 77

1. Spectroscopic Data

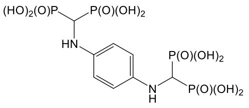

Phenylene-1,4-di(aminomethylenebisphosphonic) acid (**1**)

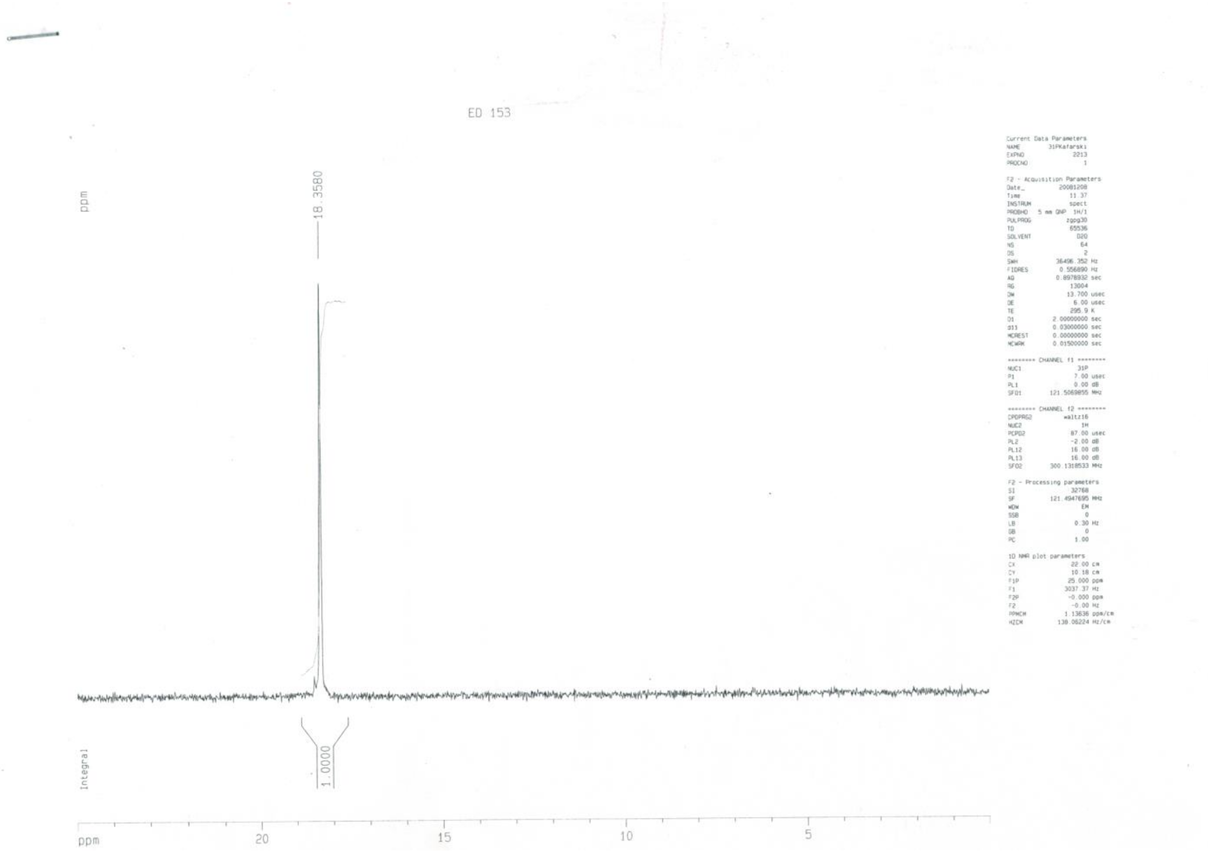

11.12.13 EC_AS_2 (1,4-fenylenodiamina)

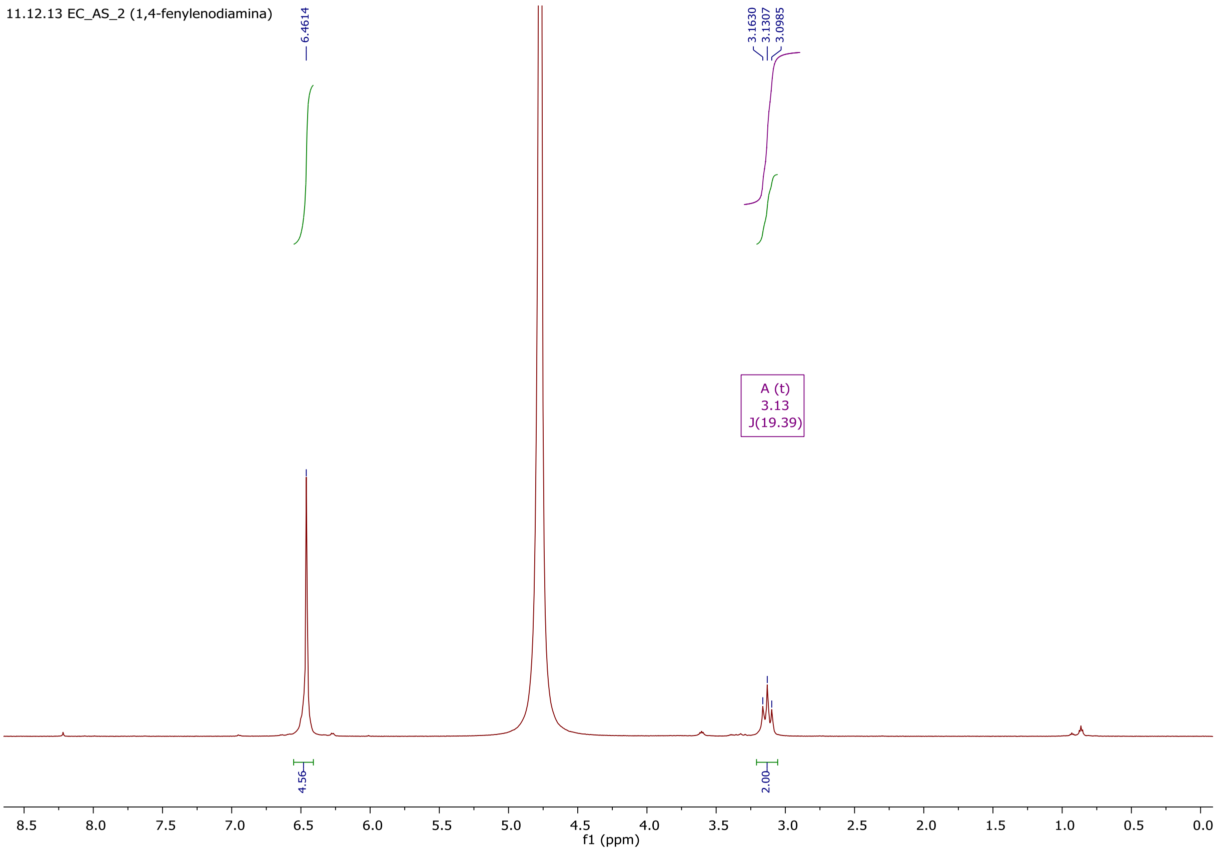

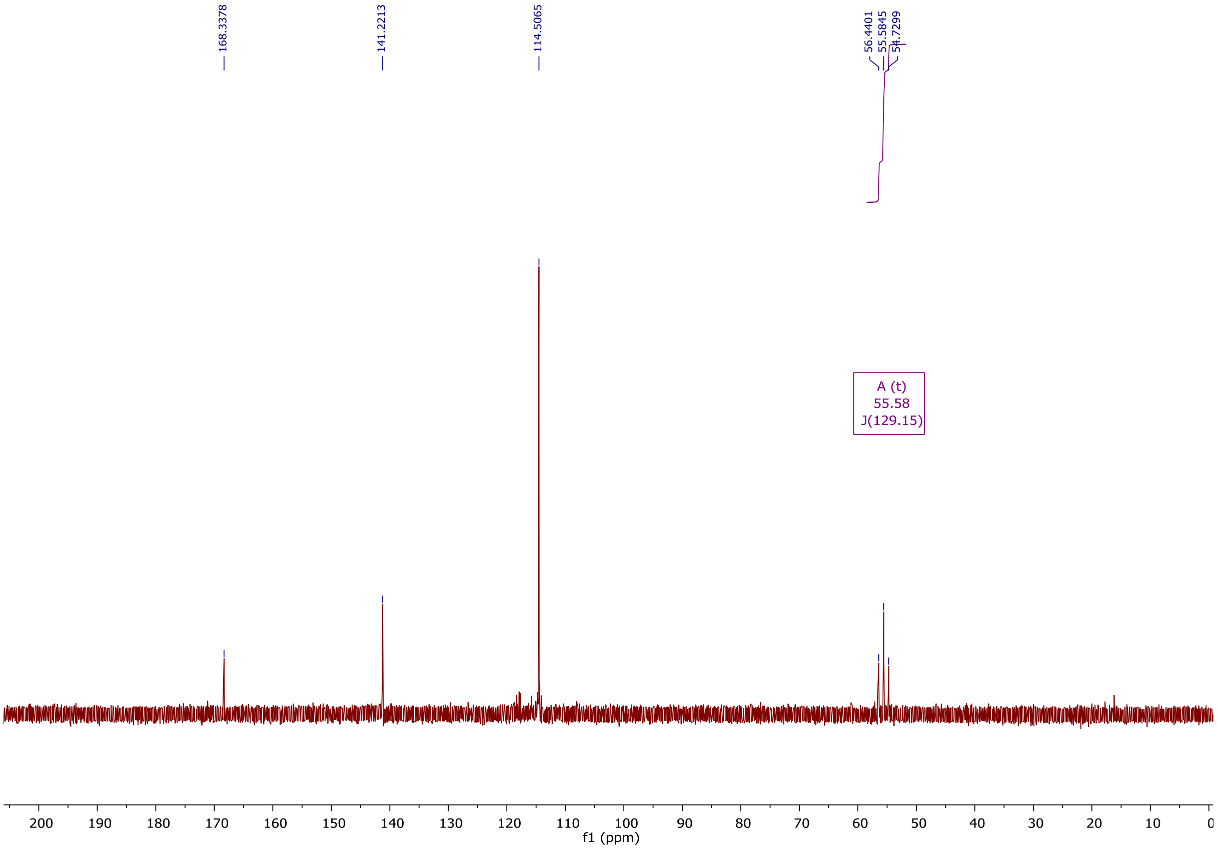

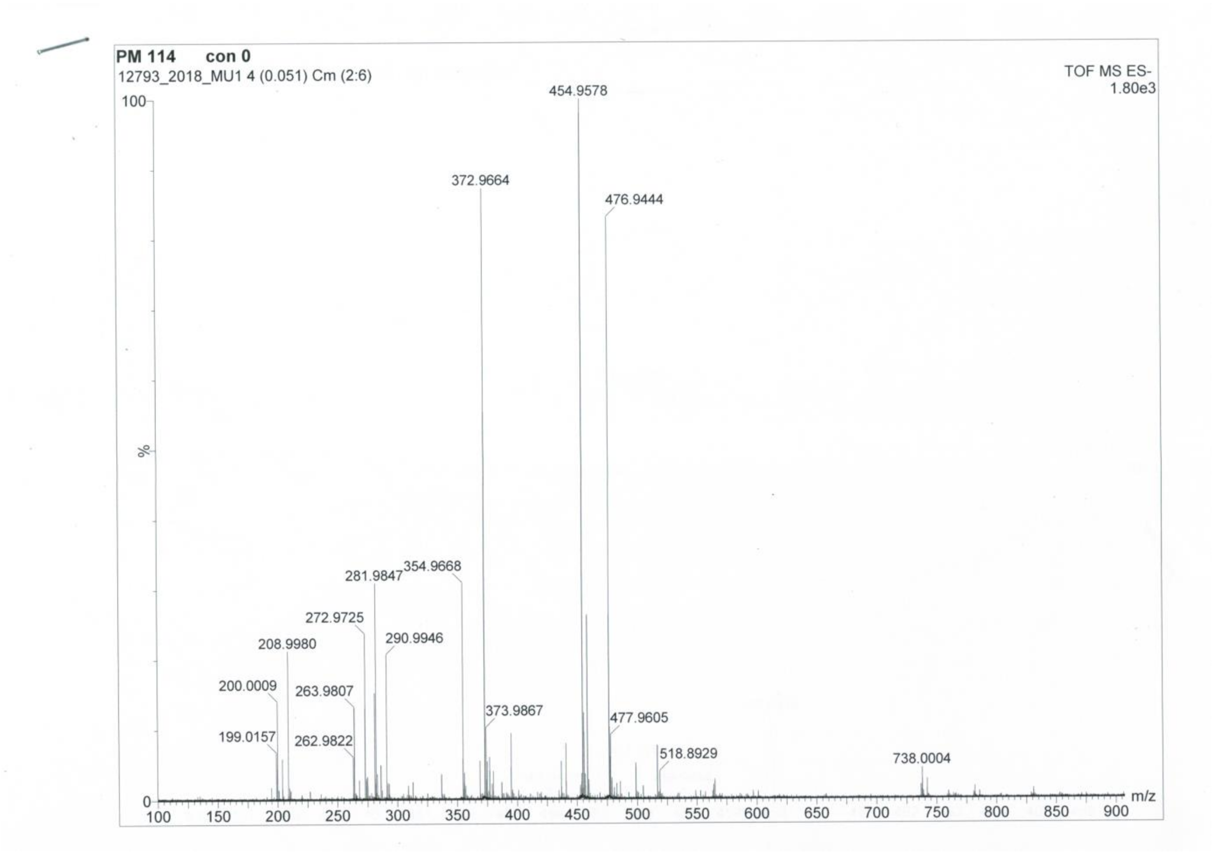

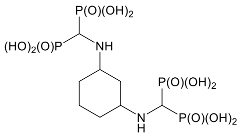

Cyclohexane-1,3-di(aminomethylenebisphosphonic) acid (**6**)

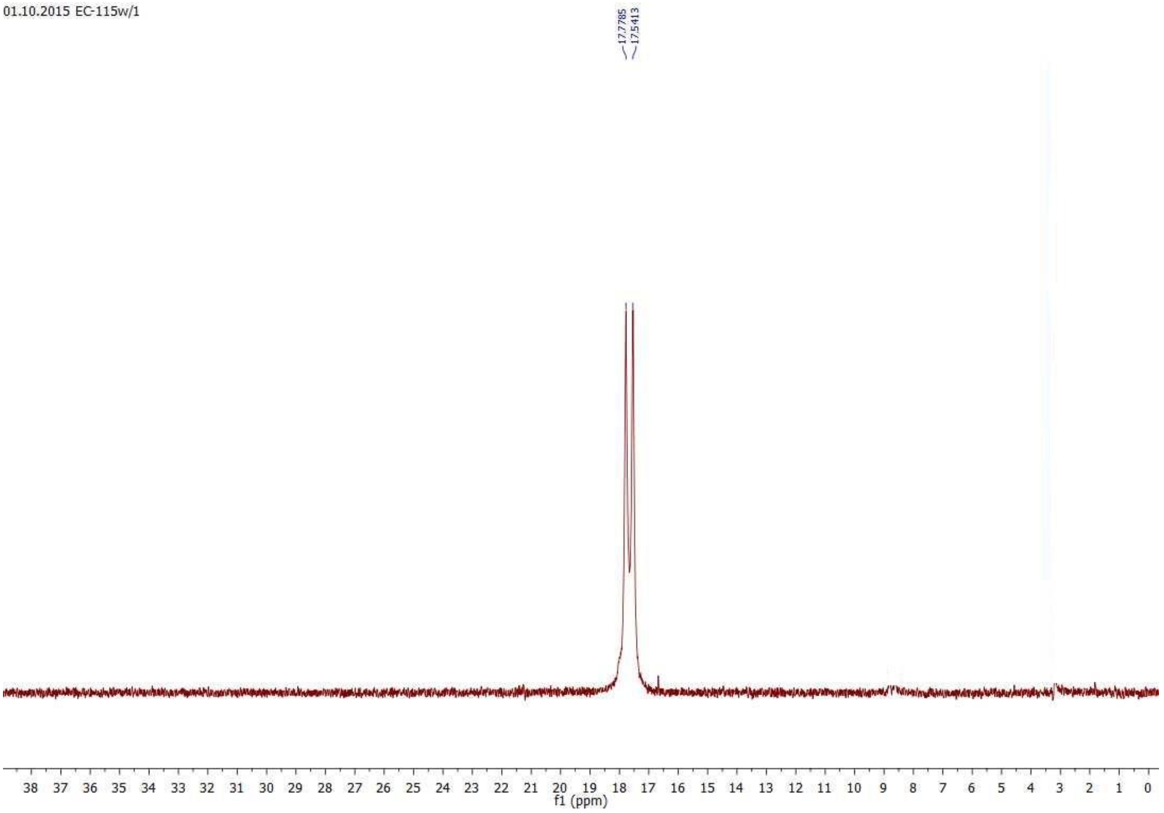

01.10.2015 EC-115w/2

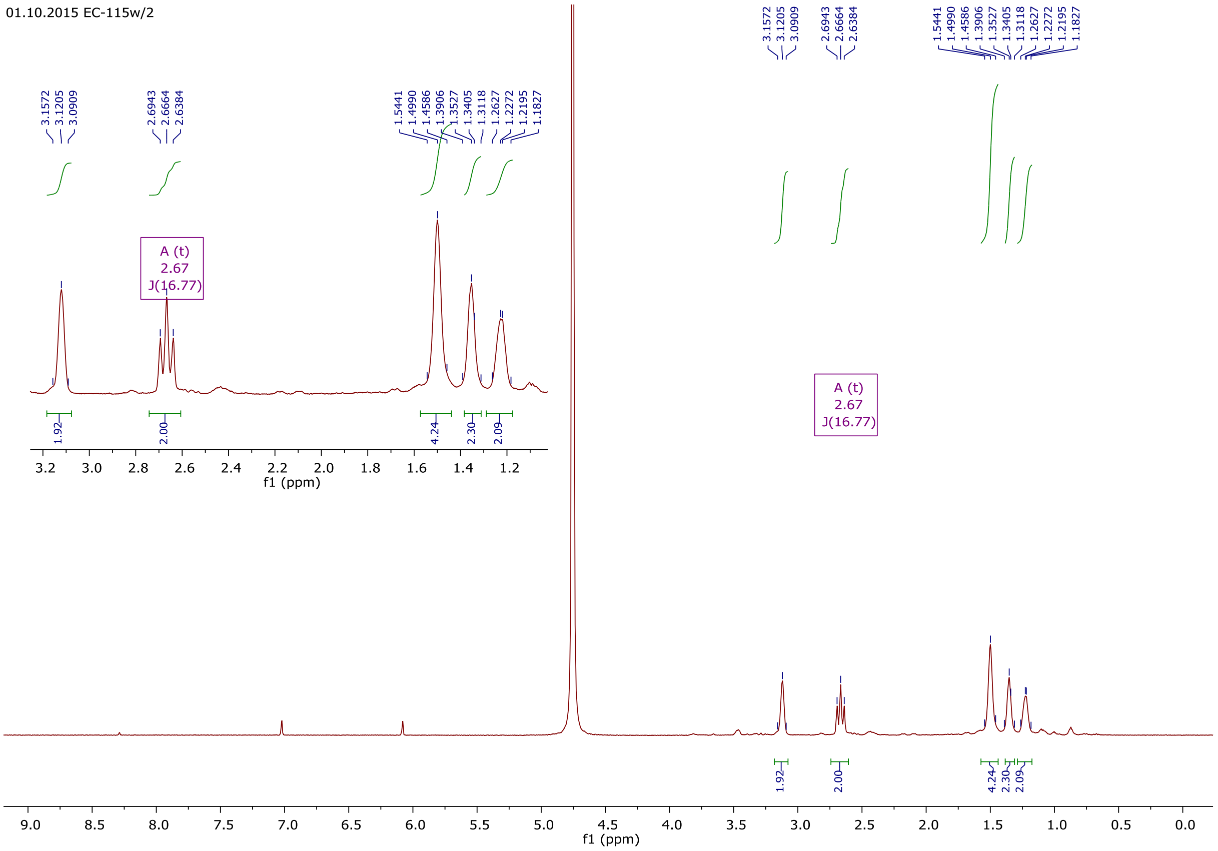

01.10.2015 EC-115w

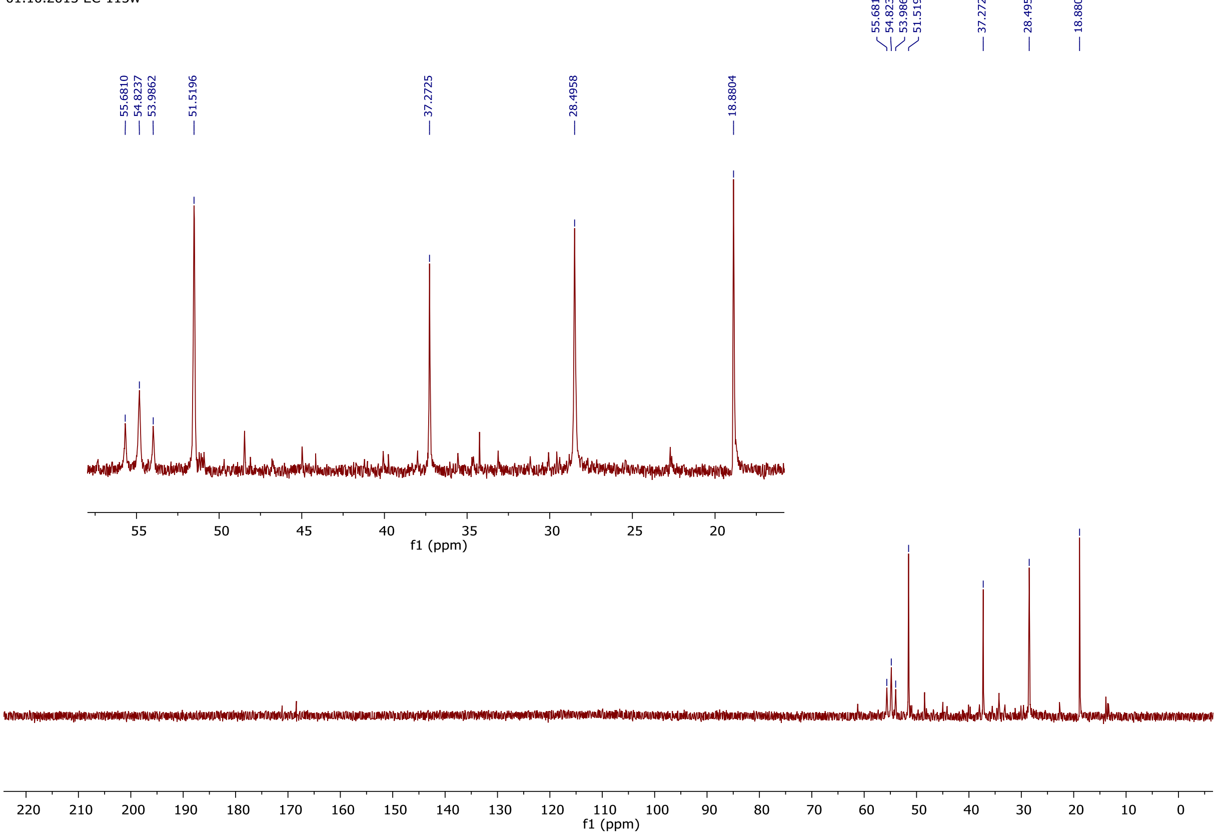

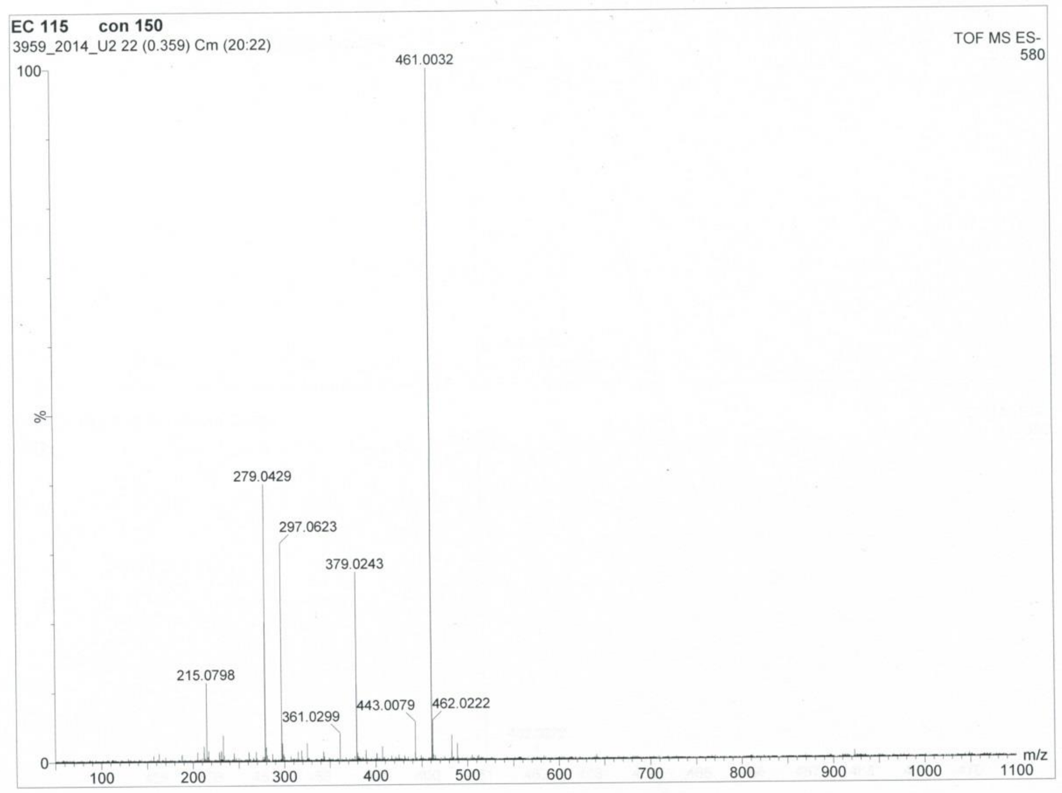

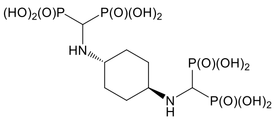

(trans)-Cyclohexane-1,4-di(aminomethylenebisphosphonic) acid (**7**)

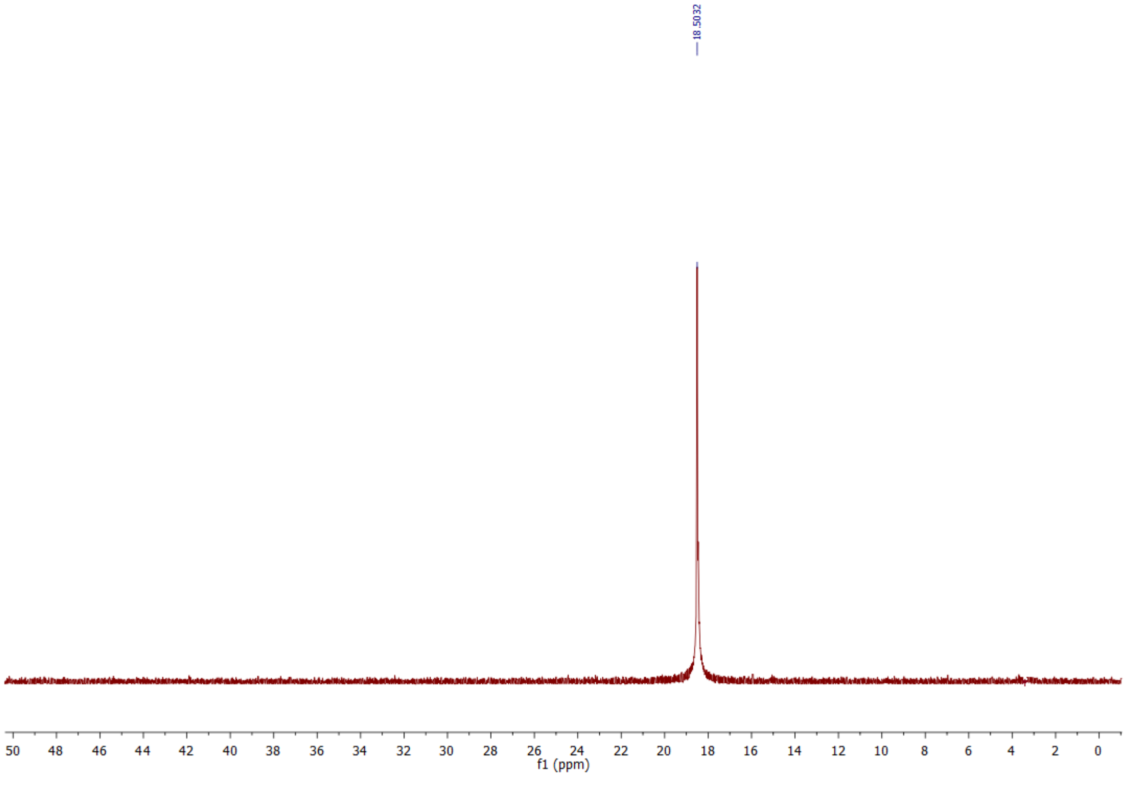

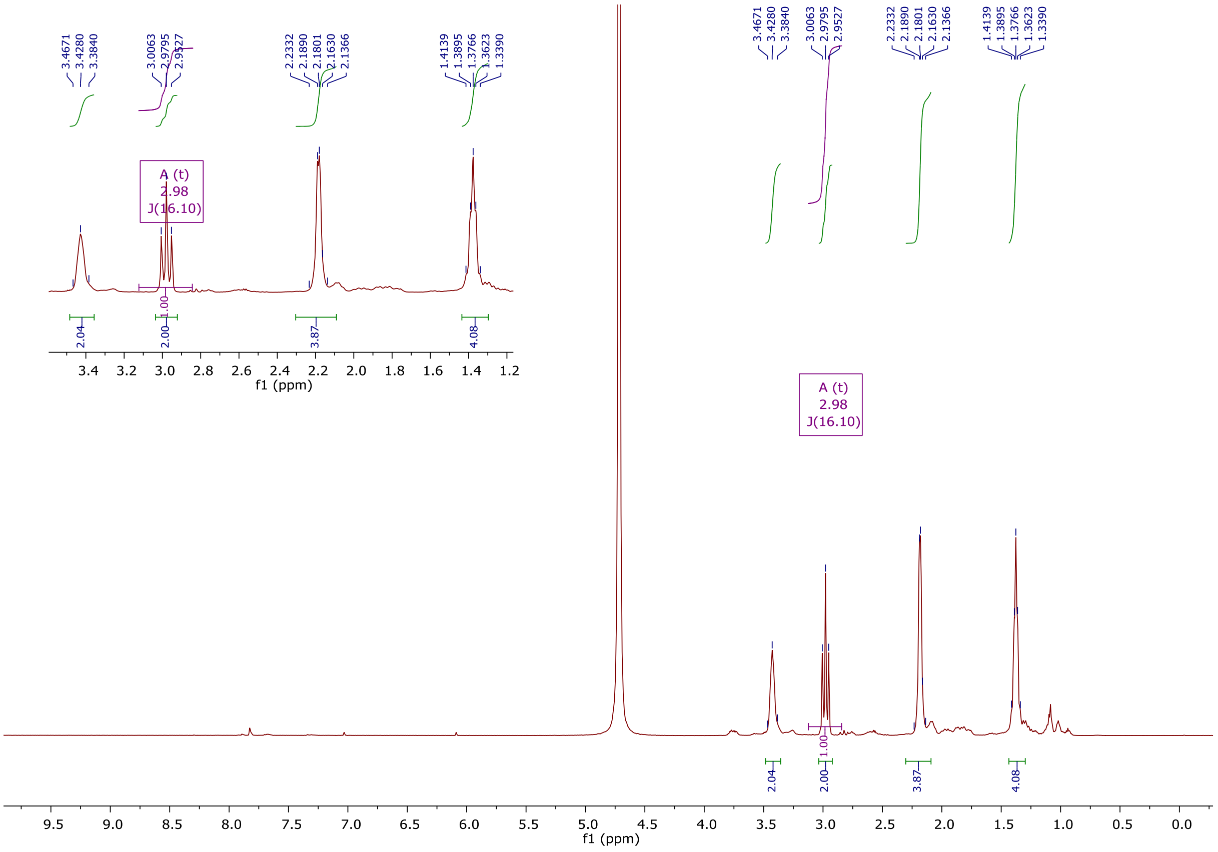
3.4671
3.4280
3.3840
3.0063
2.9795
2.9527
2.2332
2.1890
2.1801
2.1630
2.1366
1.4139
1.3895
1.3766
1.3623
1.3390
A (t)
2.98
J(16.10)
2.04
2.00
1.00
3.87
4.08
f1 (ppm)
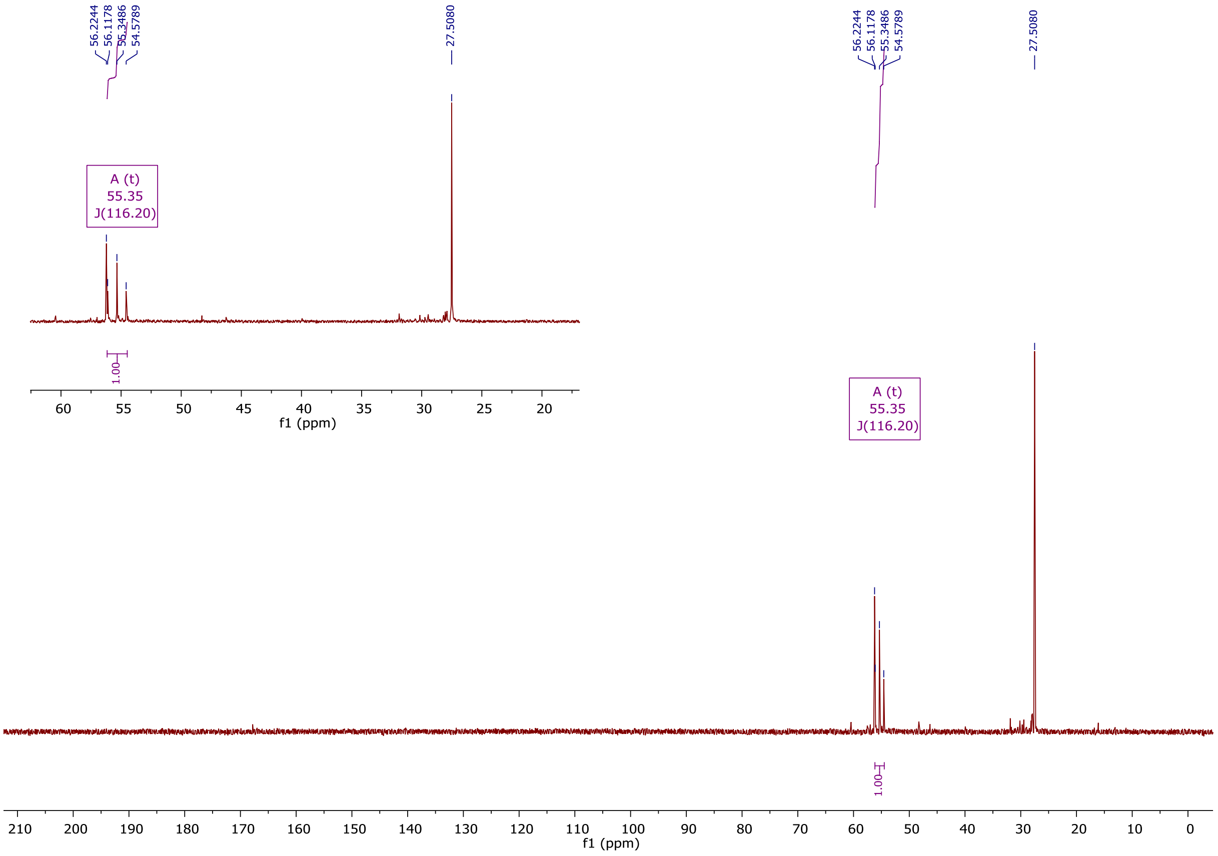
56.2244
56.1178
55.3486
54.5789
27.5080
A (t)
55.35
J(116.20)
1.00
f1 (ppm)

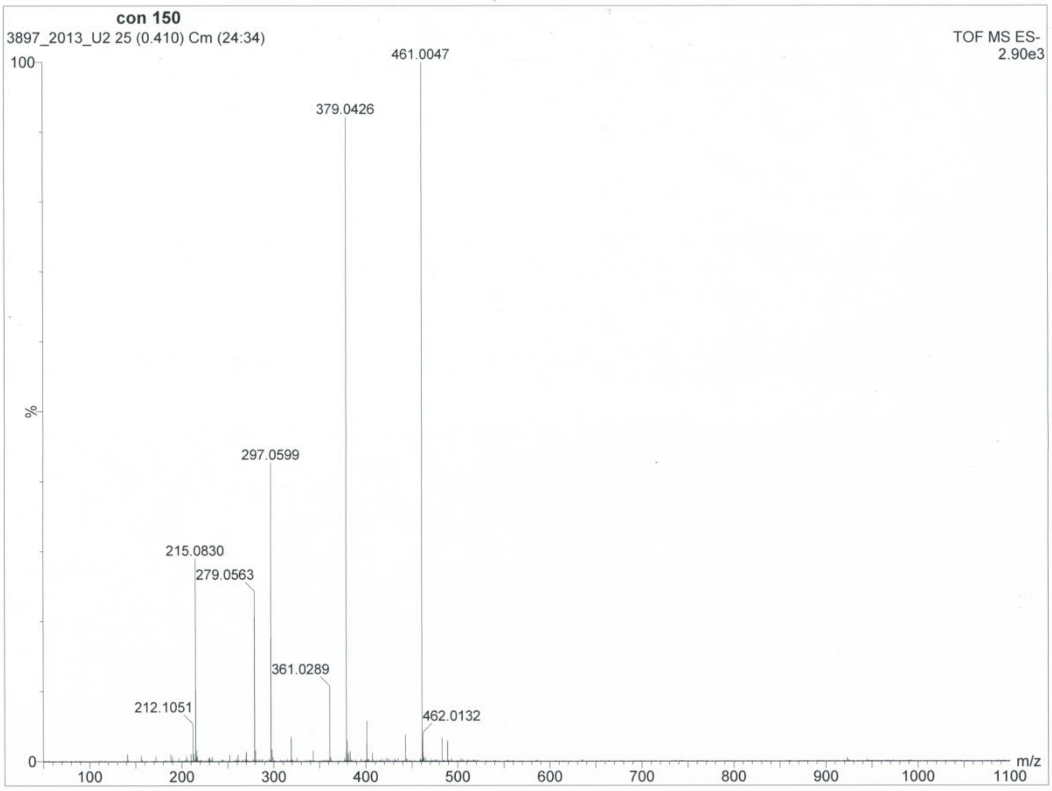

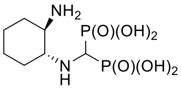

(1*S*,2*S*)-cyclohexane-1-amino-2-aminomethylenebisphosphonic acid [(1*S*,2*S*)-**8**]

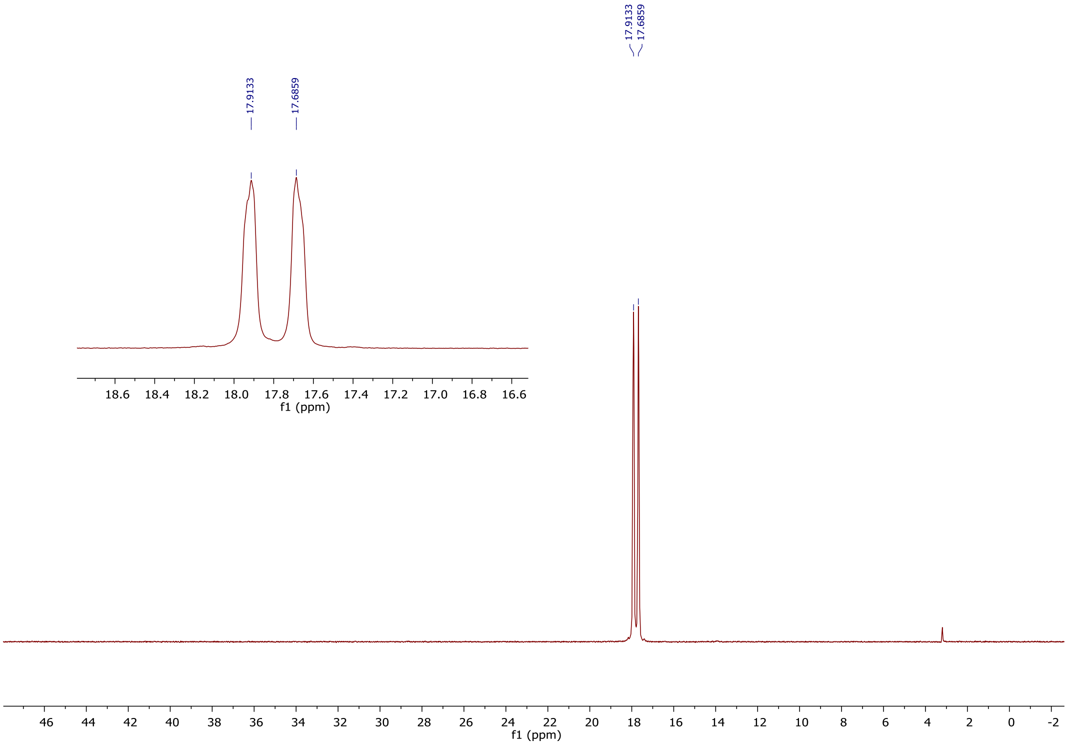

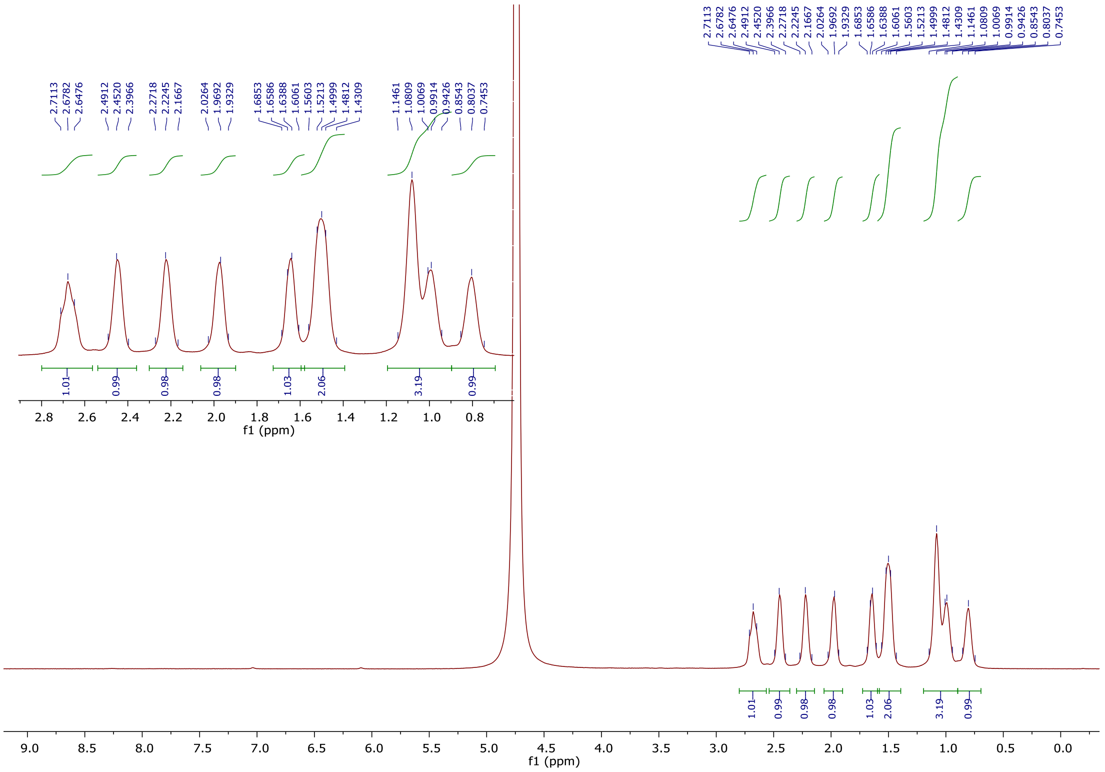
2.7113 2.6782 2.6476 2.4912 2.4520 2.3966 2.2718 2.2245 2.1667 2.0264 1.9692 1.9329 1.6853 1.6586 1.6388 1.6061 1.5603 1.5213 1.4999 1.4812 1.4309 1.1461 1.0809 1.0069 0.9914 0.9426 0.8543 0.8037 0.7453
1.01 0.99 0.98 0.98 1.03 2.06 3.19 0.99
f1 (ppm)

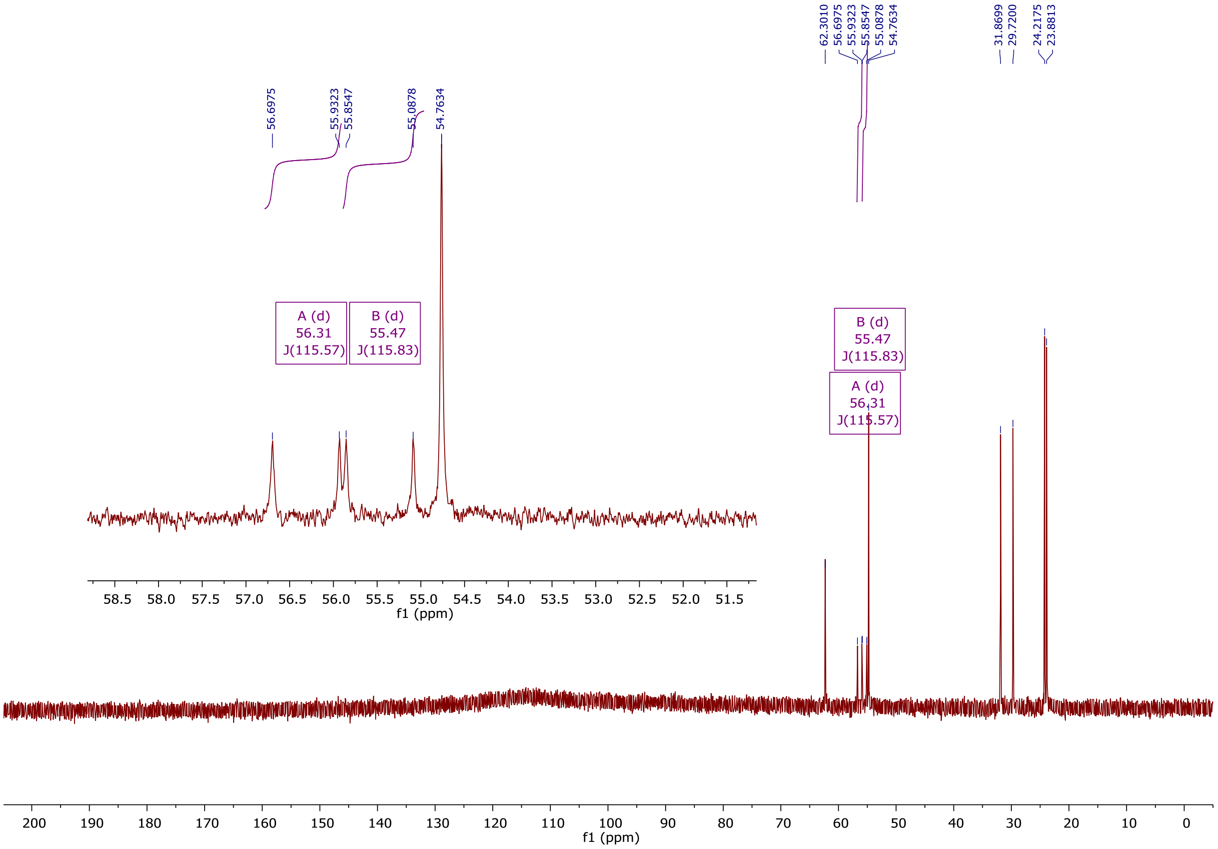
62.3010 56.6975 55.9323 55.8547 55.0878 54.7634 31.8699 29.7200 24.2175 23.8813
A (d)
56.31
J(115.57)
B (d)
55.47
J(115.83)
f1 (ppm)

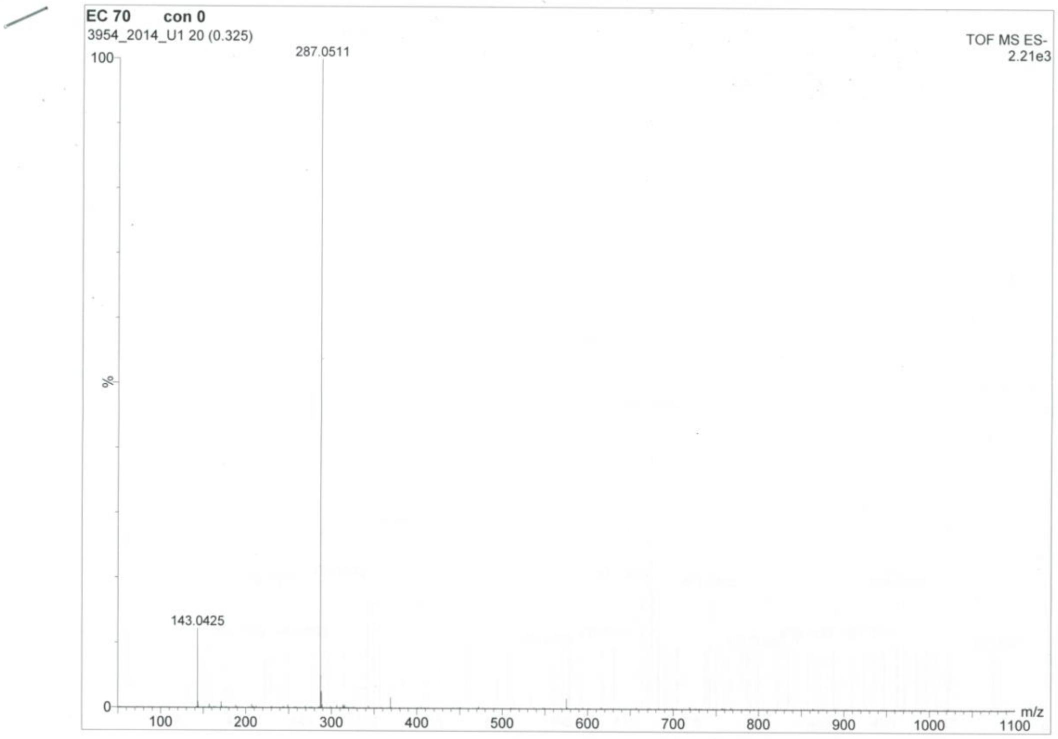

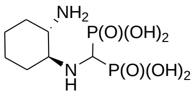

(1*R*, 2*R*)-(-)Cyclohexane-1-amino-2-aminomethylenebisphosphonic acid [(1*R*,2*R*)-**8**]

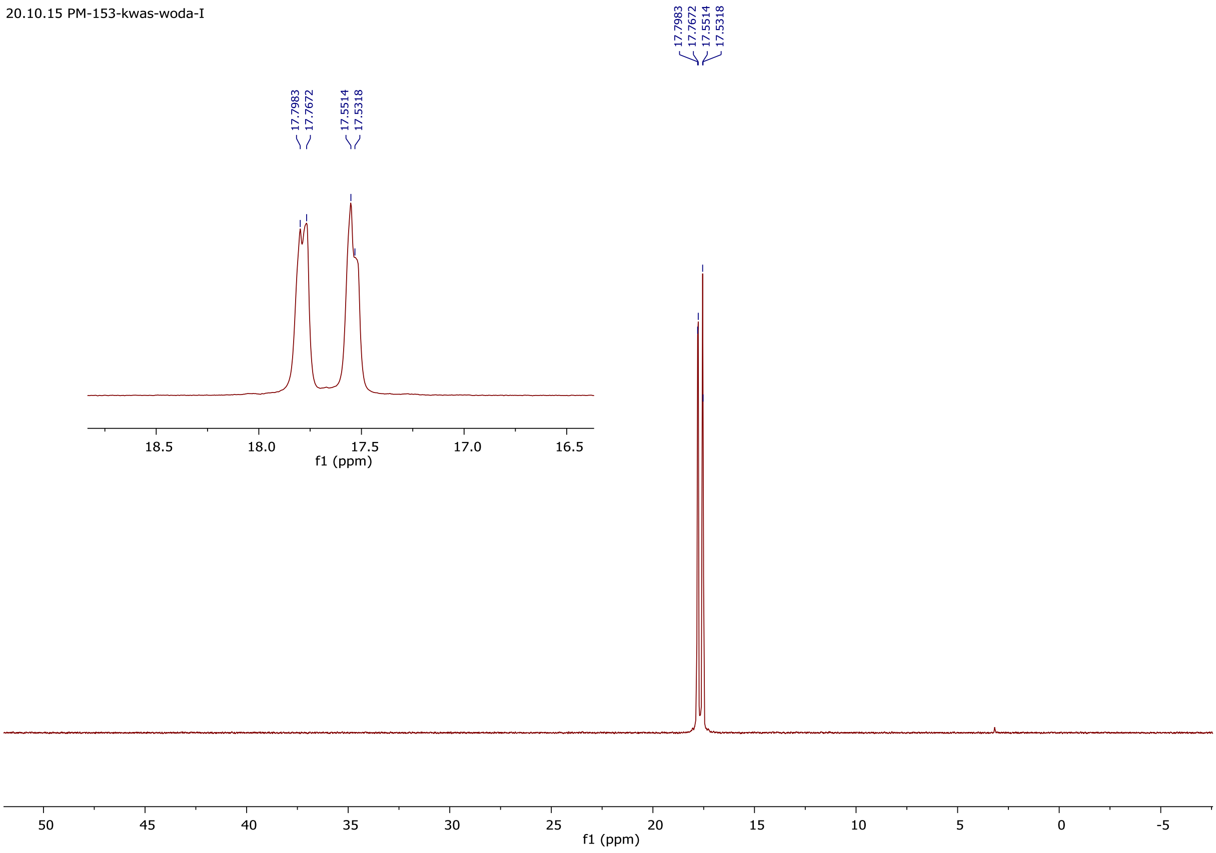

20.10.15 PM-153-kwas-woda-I

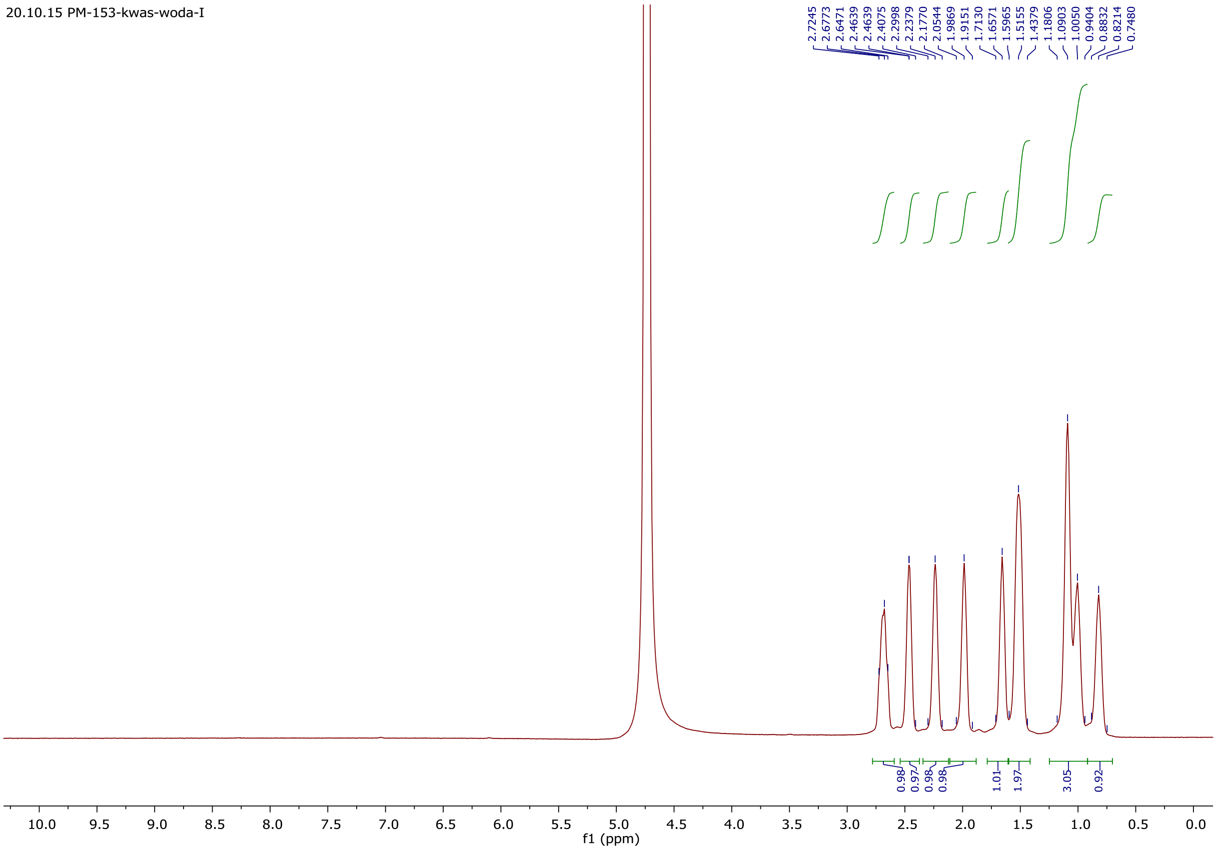

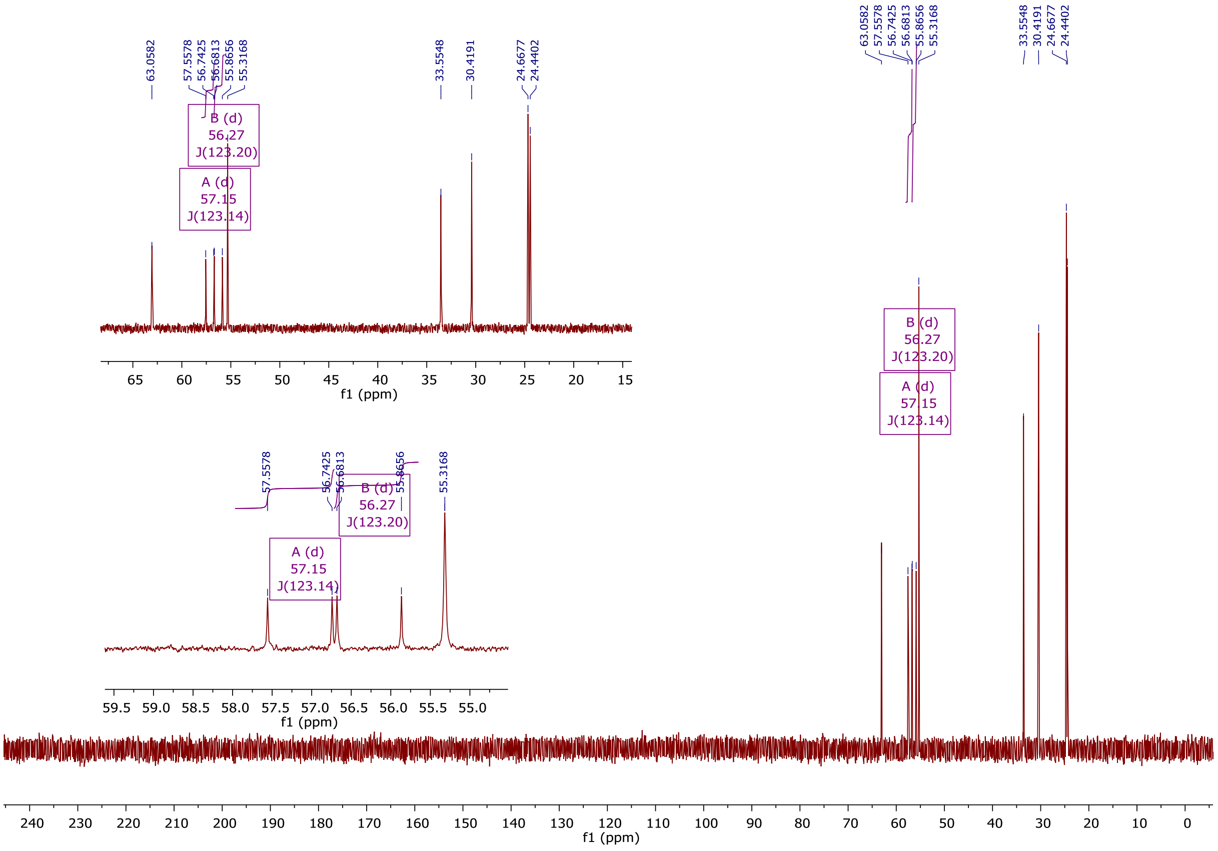

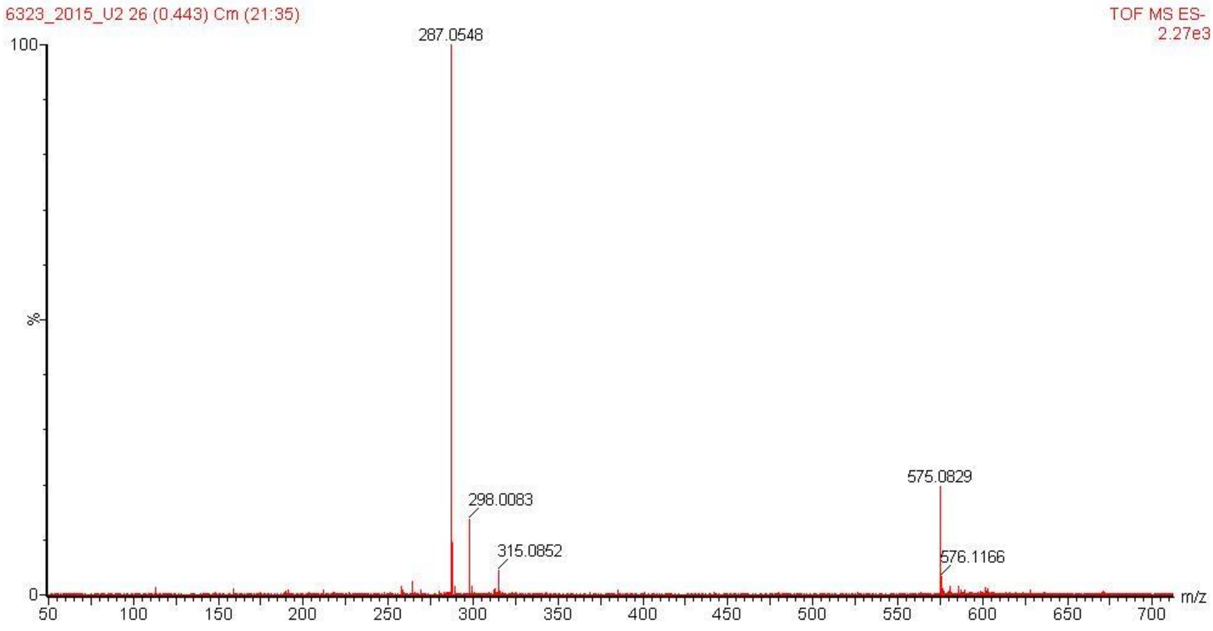

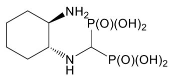

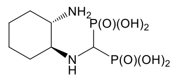

(±)-(trans)-Cyclohexane-1-amino-2-aminomethylenebisphosphonic acid (*trans*-**8**)

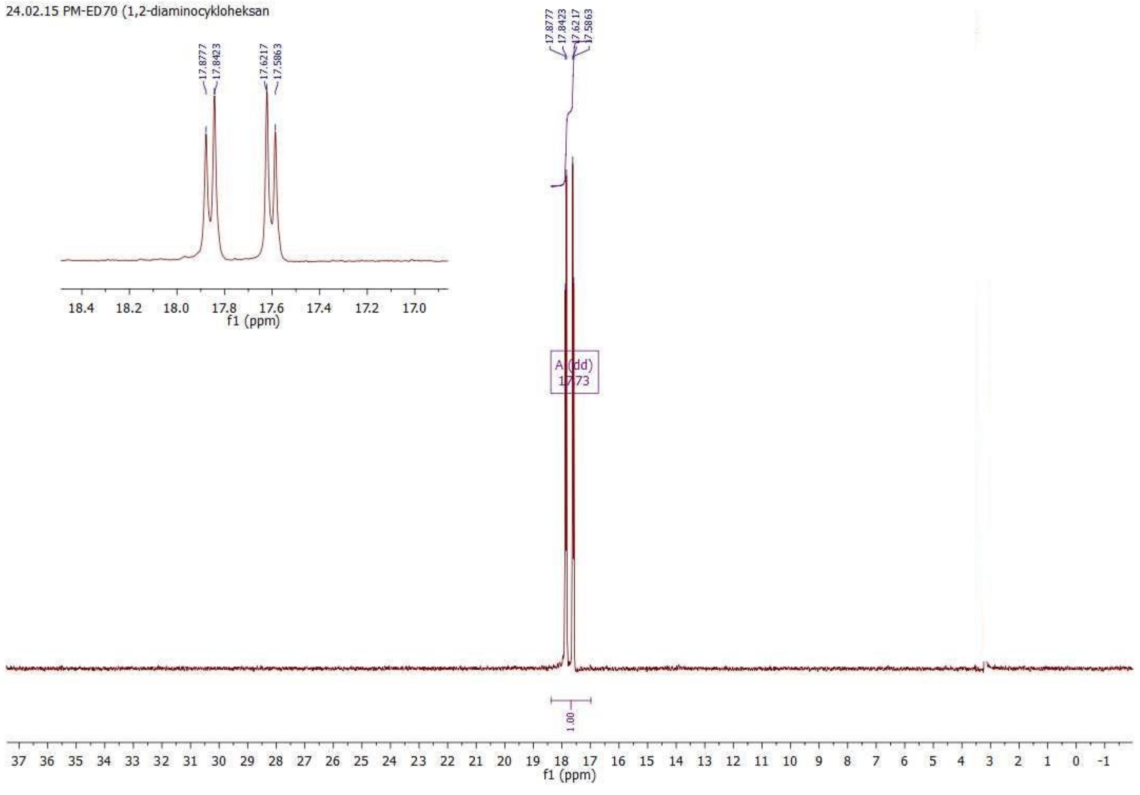

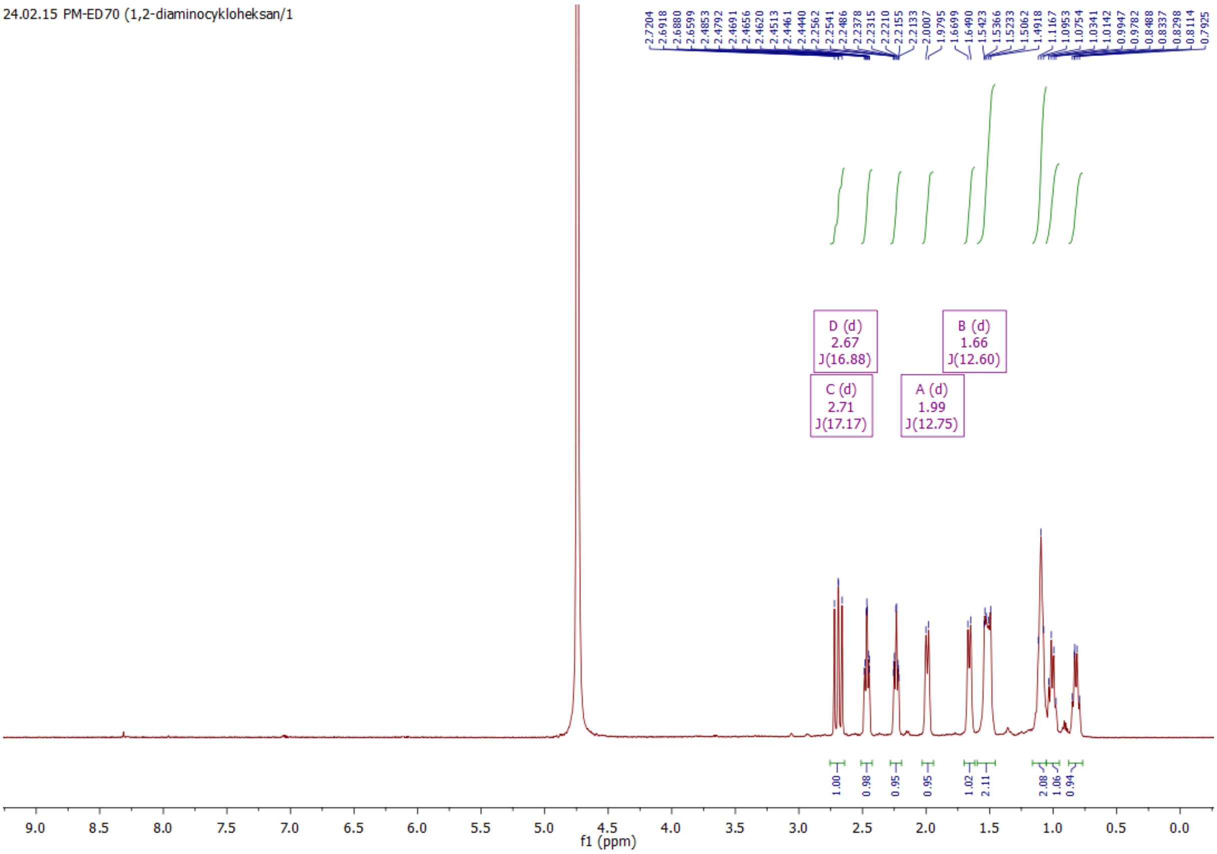
24.02.15 PM-ED70 (1,2-diaminocykloheksan/1
D (d) 2.67 J(16.88)
C (d) 2.71 J(17.17)
A (d) 1.99 J(12.75)
B (d) 1.66 J(12.60)
f1 (ppm)

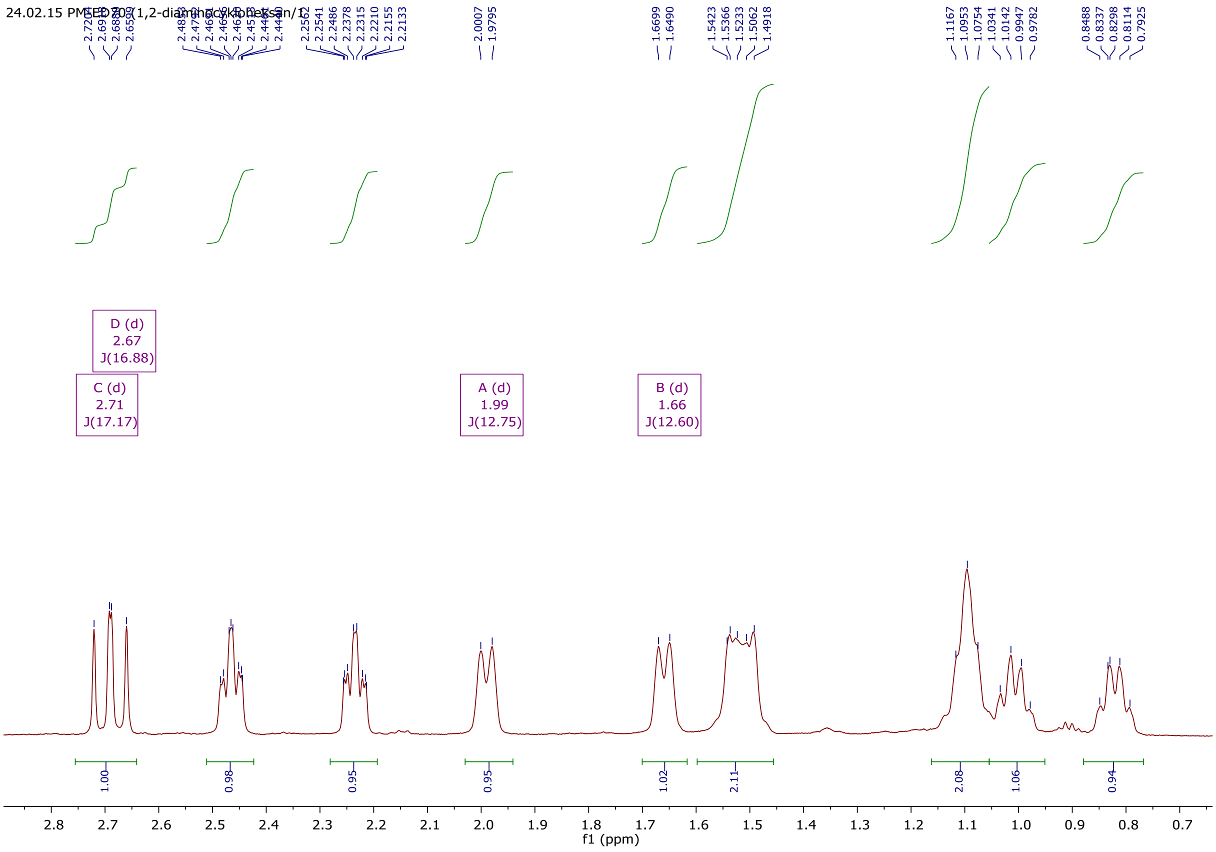
24.02.15 PM-ED70 (1,2-diaminocykloheksan/1
D (d) 2.67 J(16.88)
C (d) 2.71 J(17.17)
A (d) 1.99 J(12.75)
B (d) 1.66 J(12.60)
f1 (ppm)

03.03.15 PM-ED-70-13C (1,2-diaminocykloheksan)/5

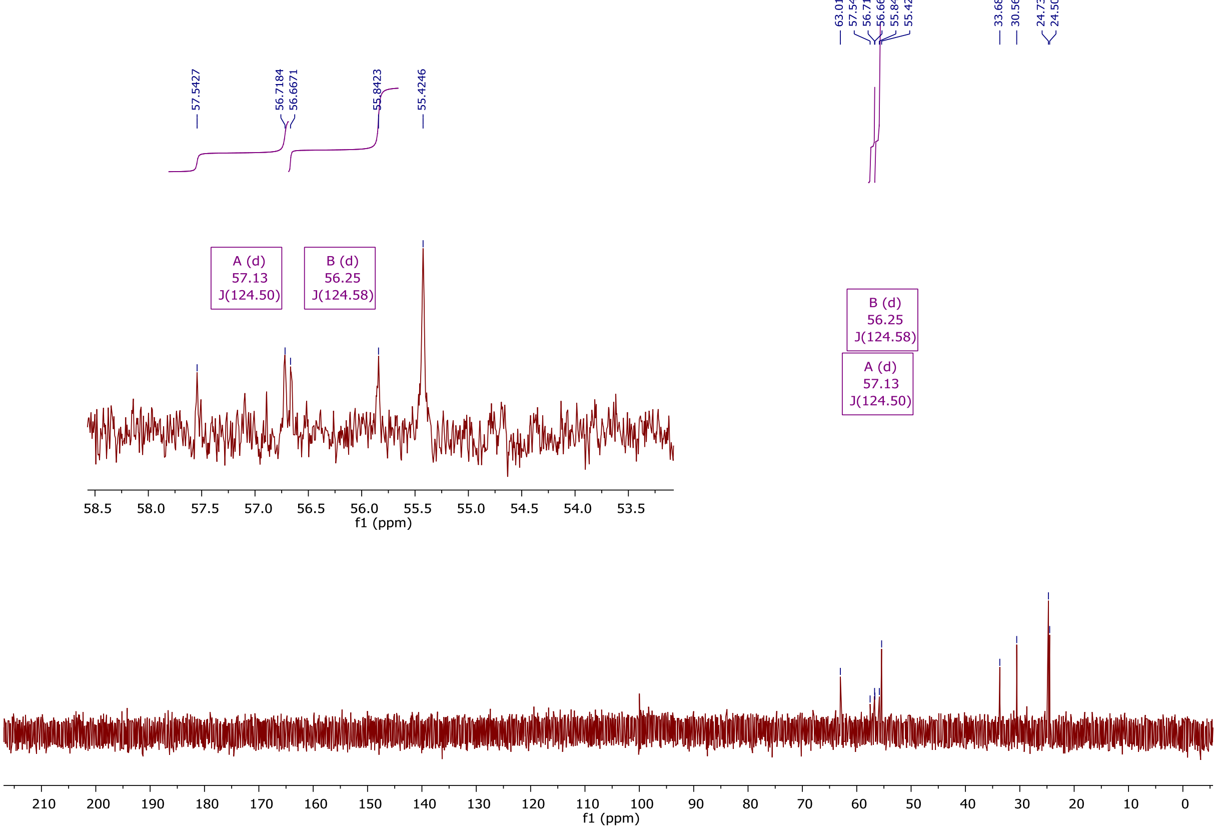

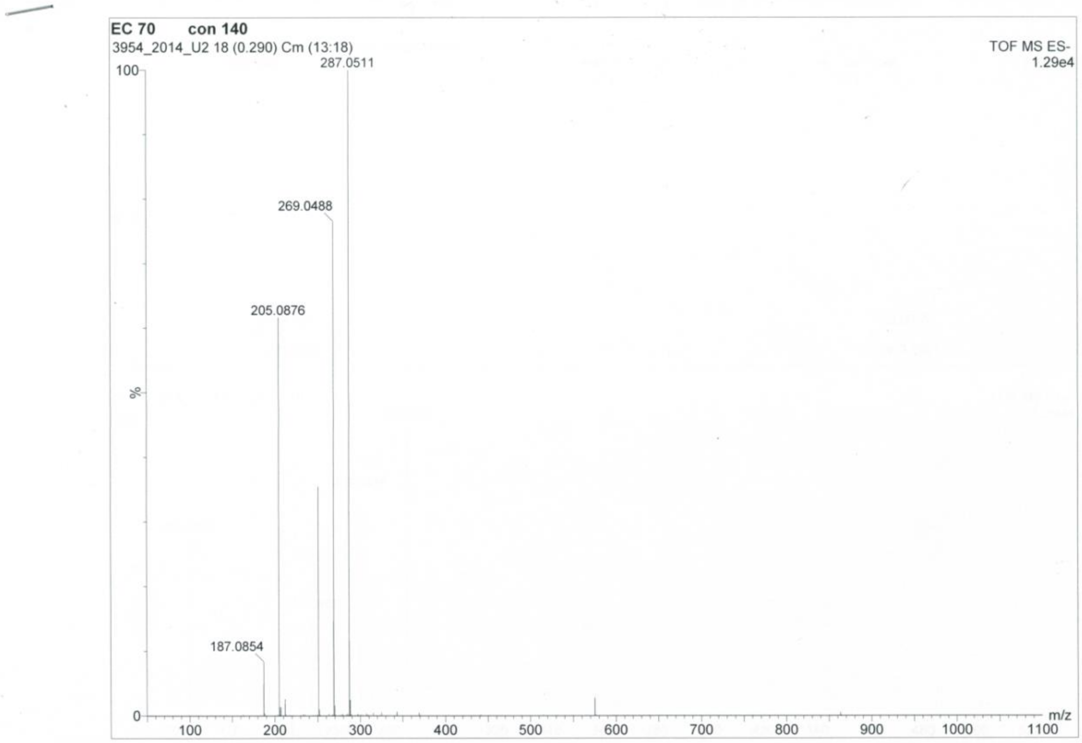

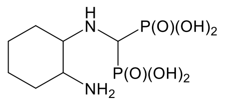

Racemic cyclohexane-1-amino-2-aminomethylenebisphosphonic acid [(rac)-**8**]

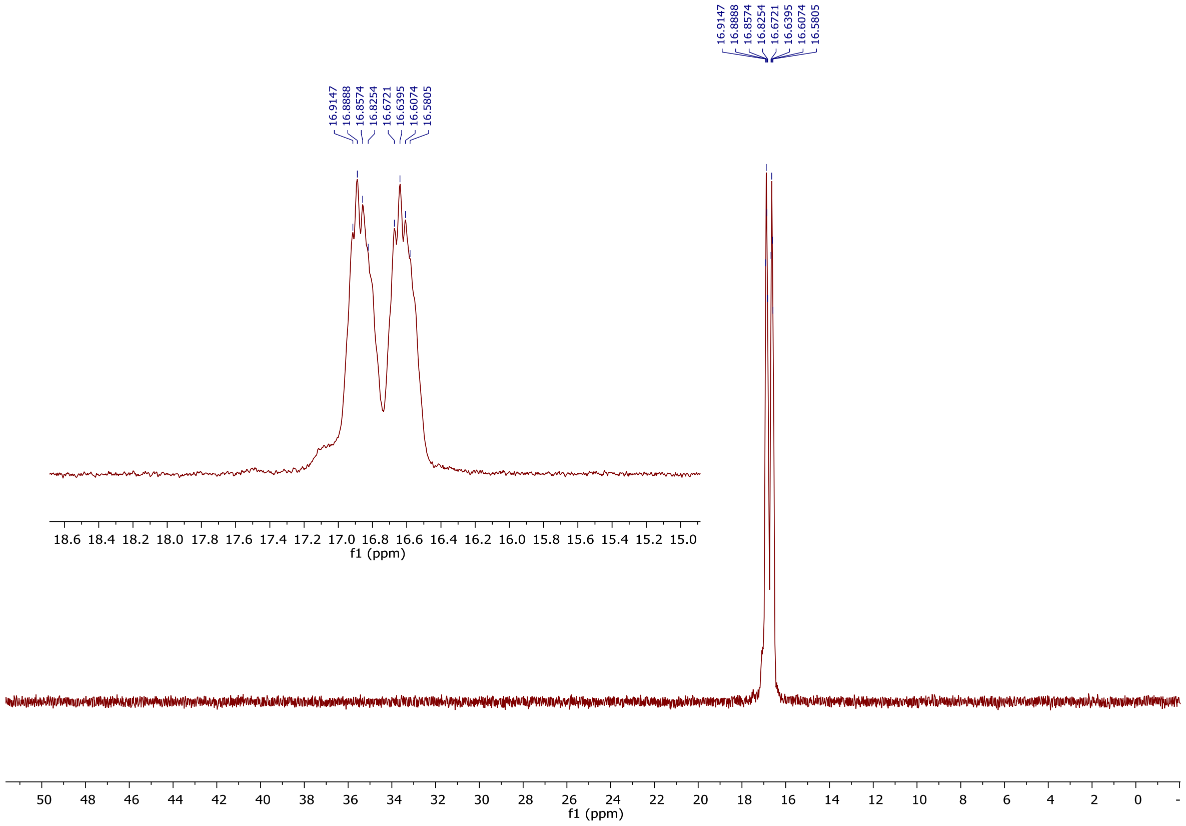

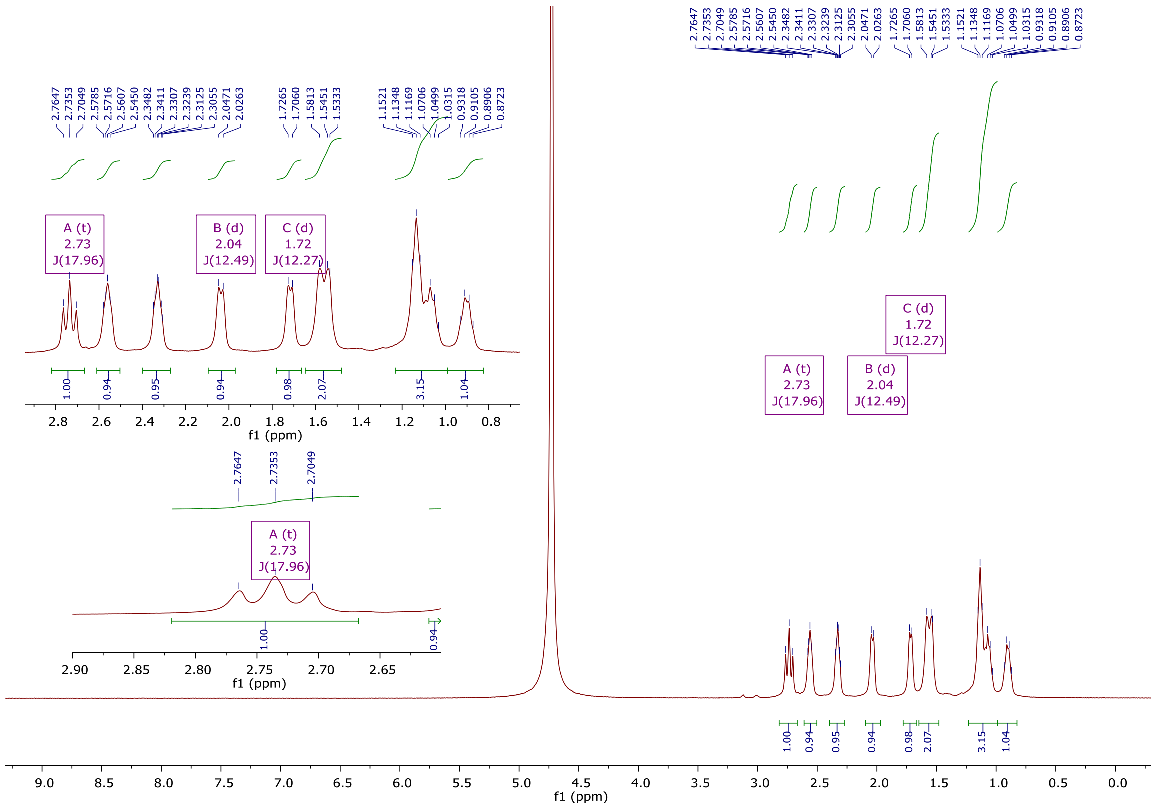

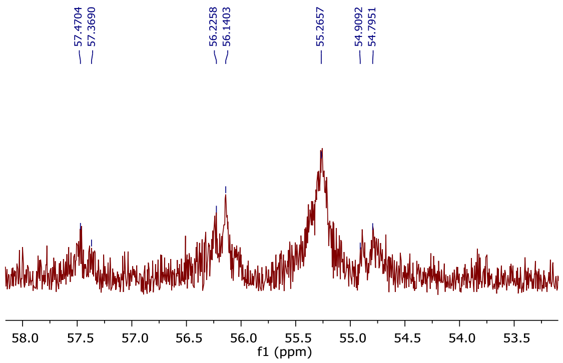
57.4704
57.3690
56.2258
56.1403
55.2657
54.9092
54.7951
58.0
57.5
57.0
56.5
56.0
55.5
55.0
54.5
54.0
53.5
f1 (ppm)

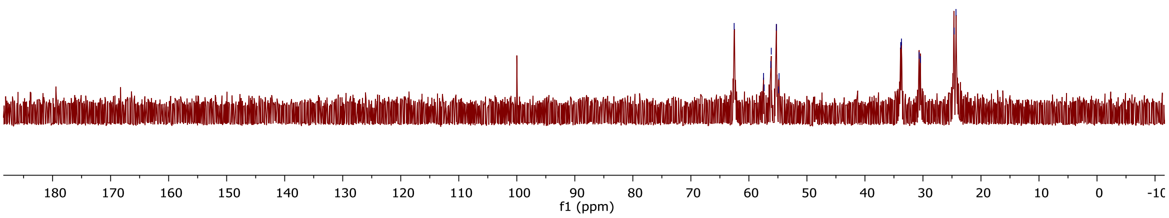
62.5385
57.4704
57.3690
56.2258
56.1403
55.2657
54.9092
54.7951
33.8291
33.7062
30.6534
30.4351
24.6190
24.3079
180
170
160
150
140
130
120
110
100
90
80
70
60
50
40
30
20
10
0
f1 (ppm)

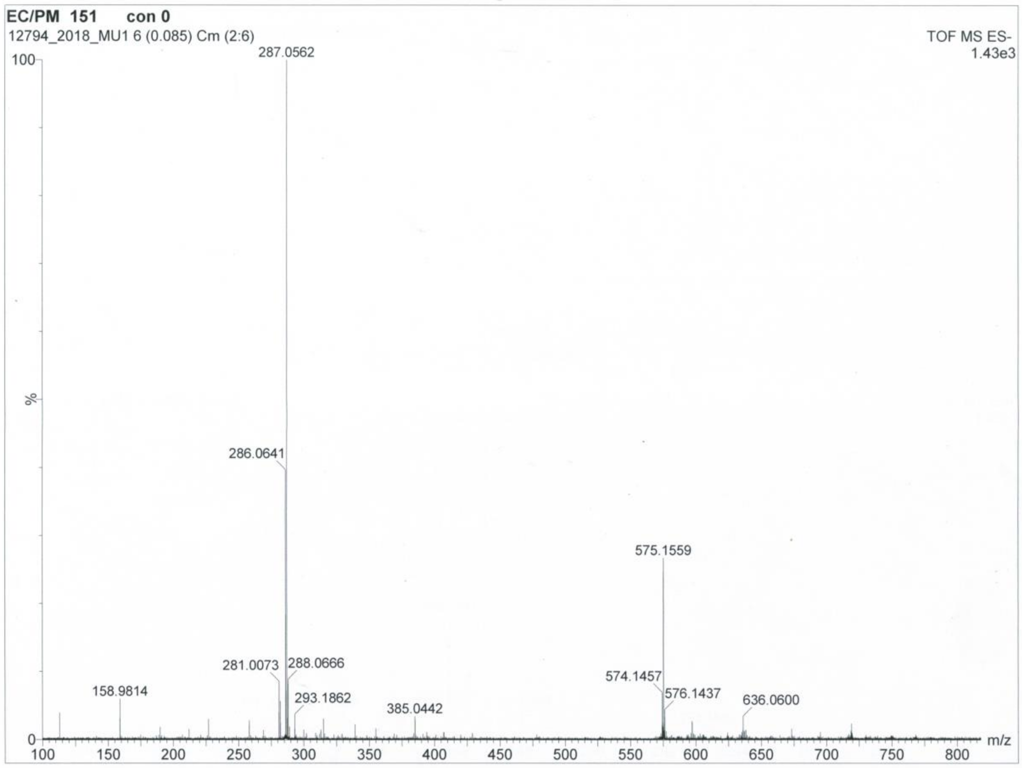
EC/PM 151 con 0
12794_2018_MU1 6 (0.085) Cm (2:6)
TOF MS ES-
1.43e3
100
%
0
287.0562
286.0641
575.1559
281.0073
288.0666
158.9814
293.1862
385.0442
574.1457
576.1437
636.0600
100
150
200
250
300
350
400
450
500
550
600
650
700
750
800
m/z

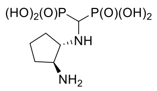

(*1S,2S*)-Cyclopentane-1-amino-2-aminomethylenebisphosphonic acid [(1S,2S)-**9]**

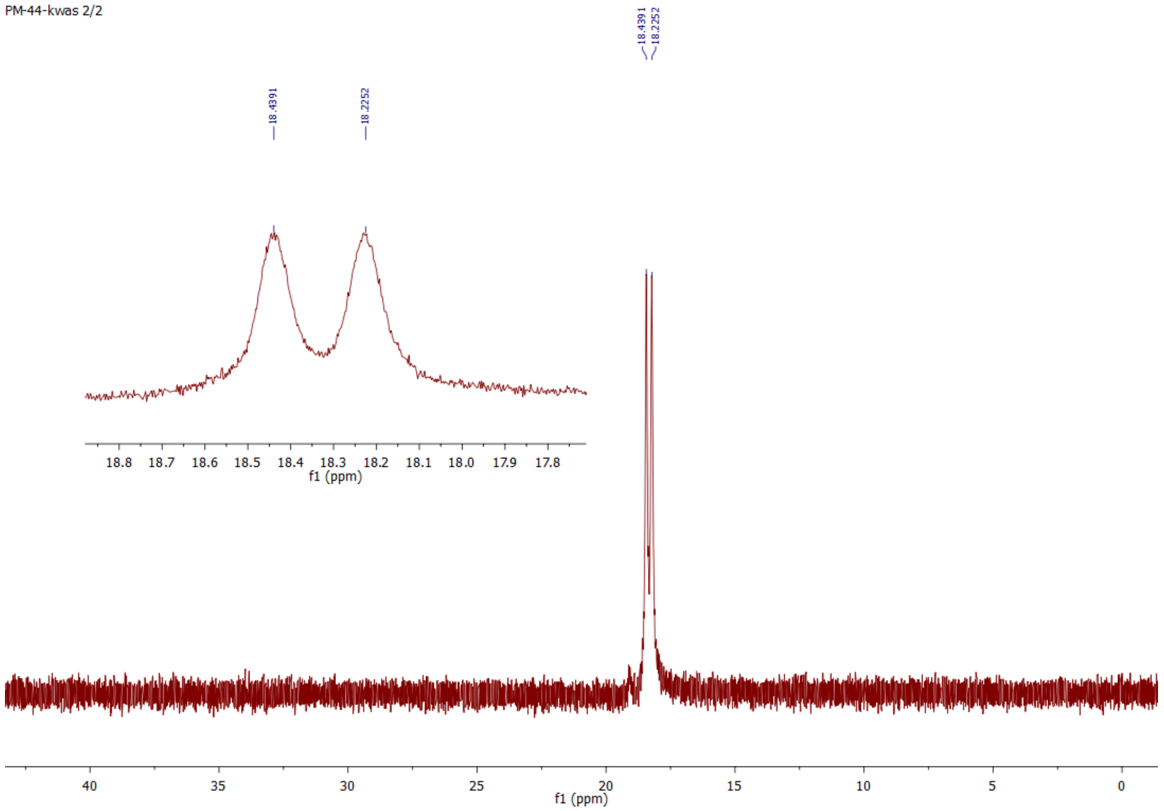

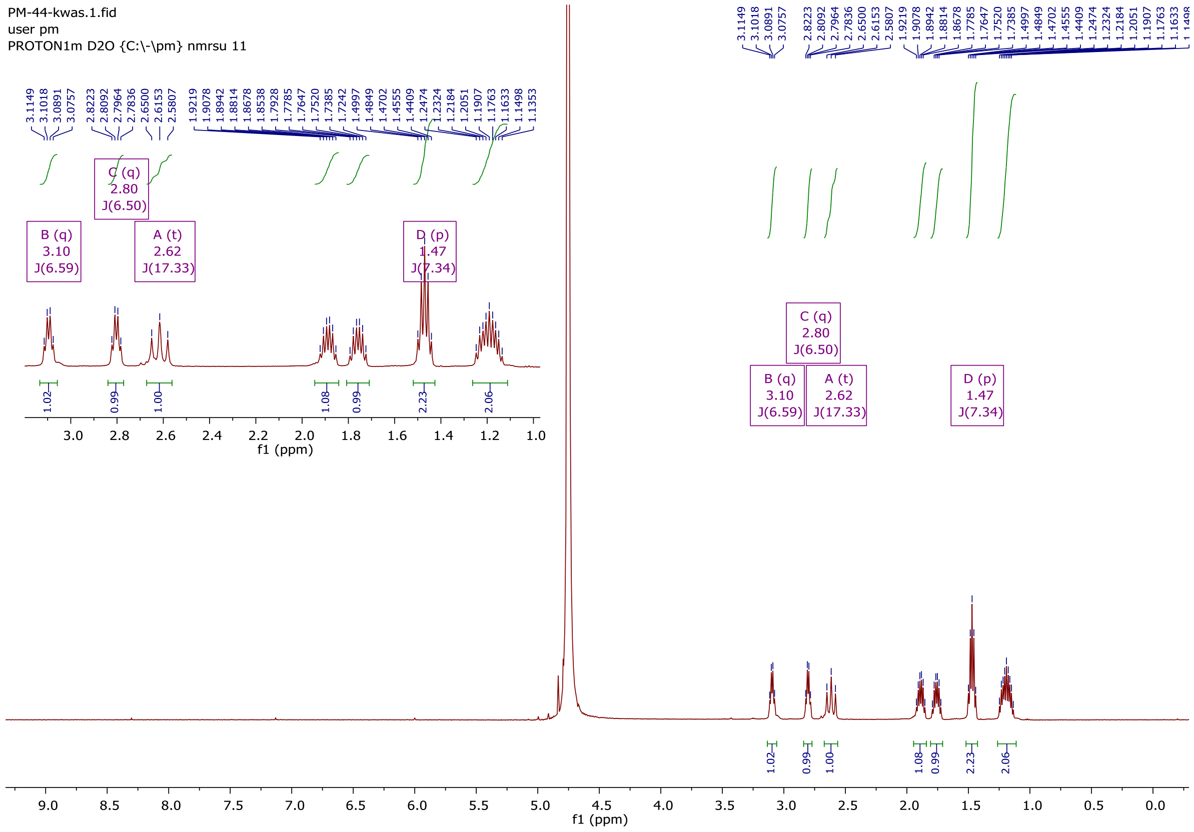

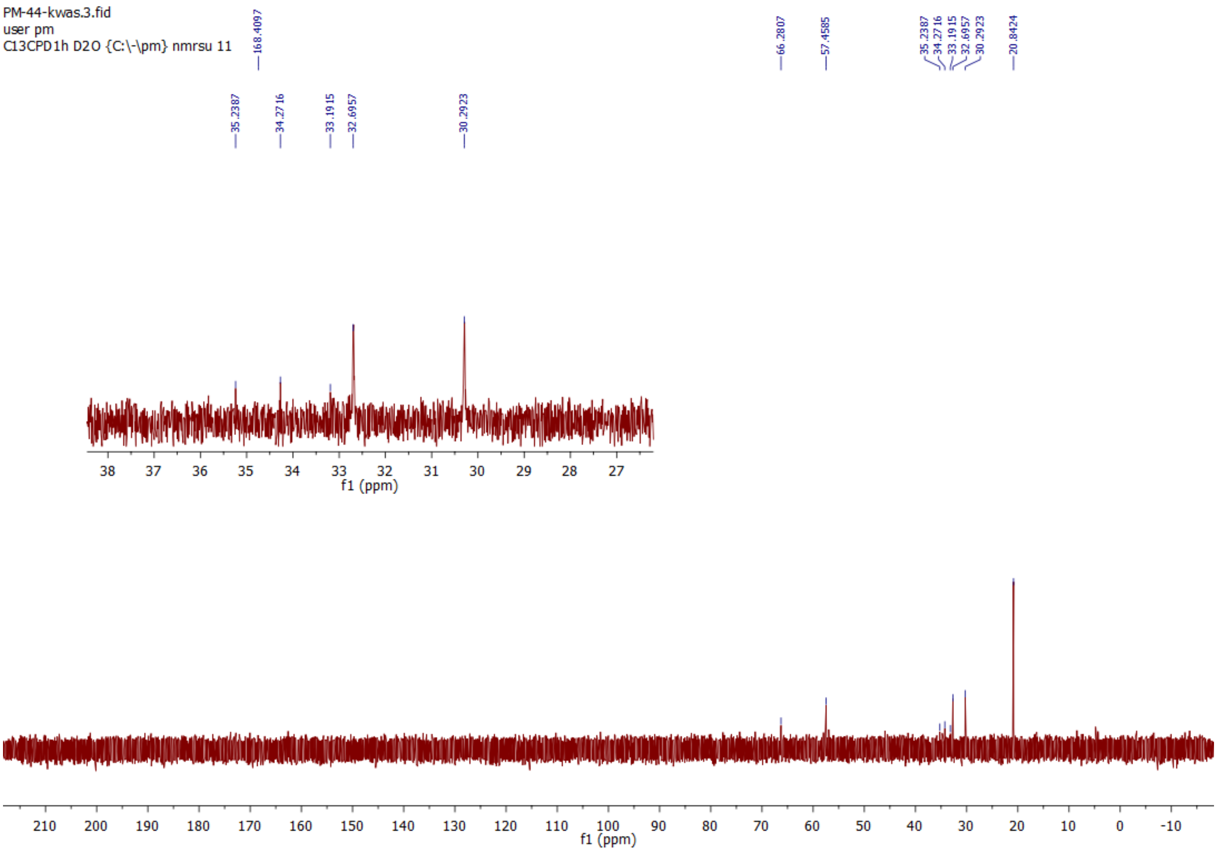
PM-44-kwas.3.fid
user pm
C13CPD1h D2O {C:\-\pm} nmrsu 11
168.4097
66.2807
57.4585
35.2387
34.2716
33.1915
32.6957
30.2923
20.8424
f1 (ppm)

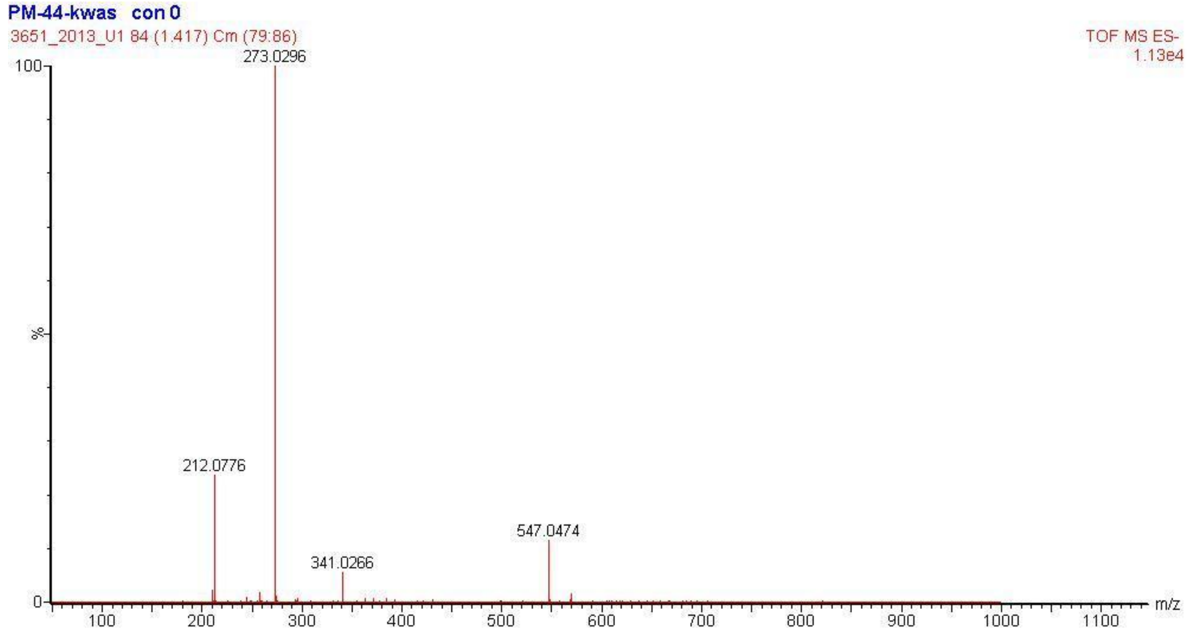
PM-44-kwas con 0
3651_2013_U1 84 (1.417) Cm (79:86)
TOF MS ES-
1.13e4
273.0296
212.0776
547.0474
341.0266
%
m/z

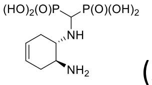

(*1S,2S*)(+)- 4-Cyclohexene-1-amino-2-aminomethylenebisphosphonic acid [(1S,2S)-**10]**

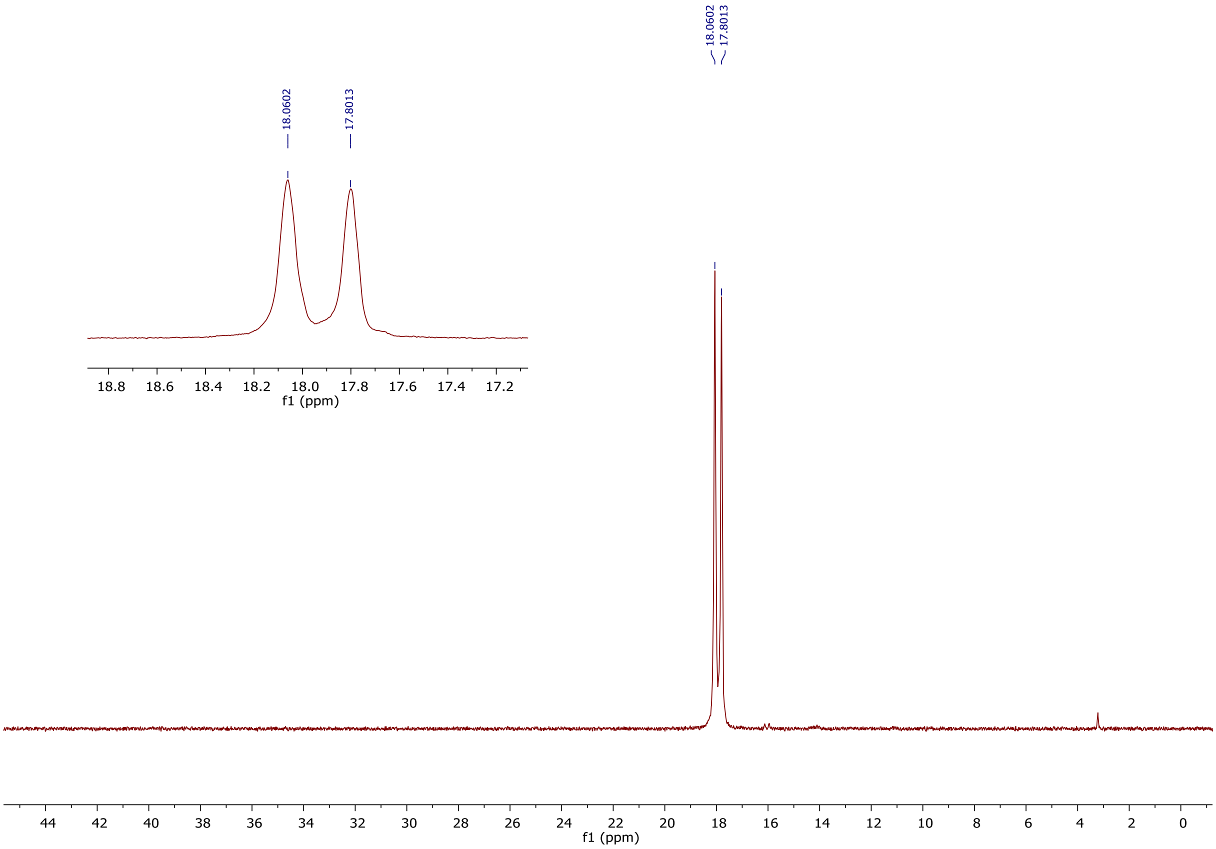

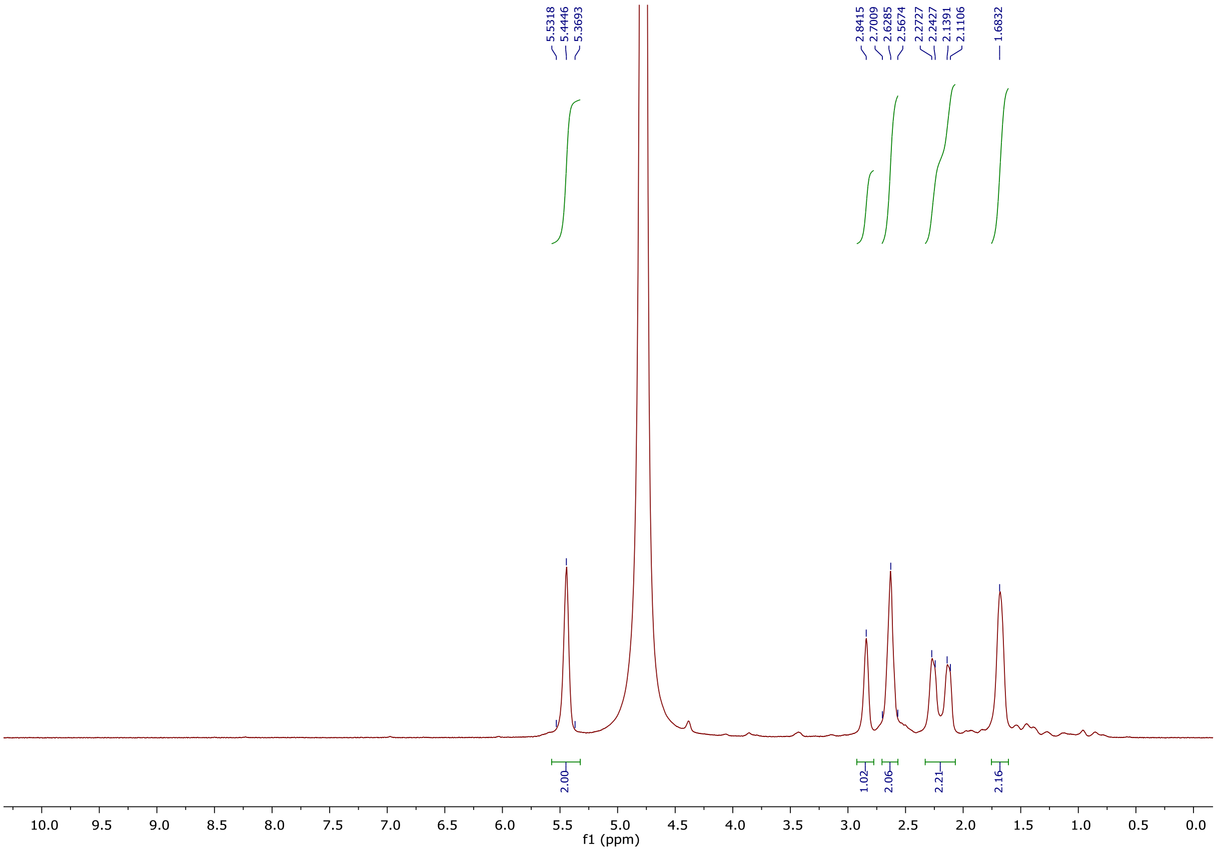

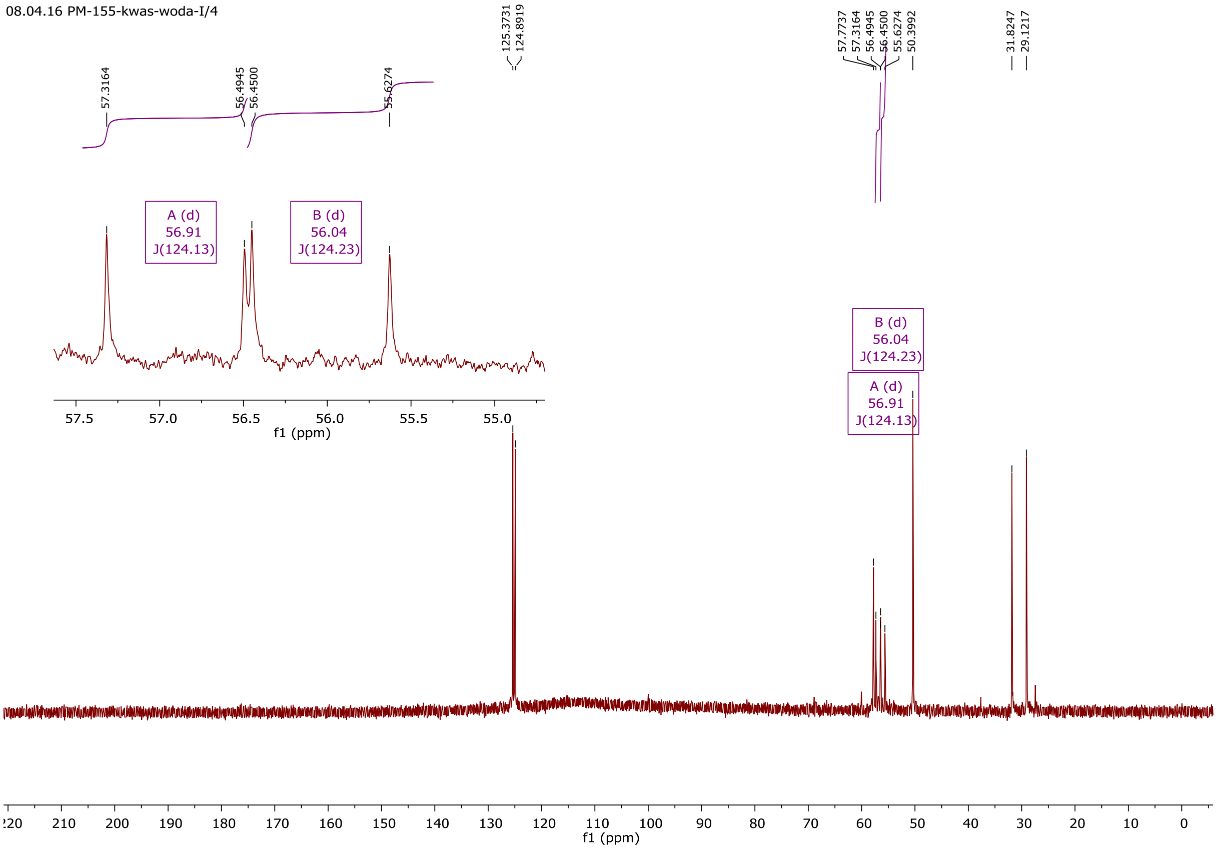
08.04.16 PM-155-kwas-woda-I/4
125.3731
124.8919
57.7737
57.3164
56.4945
56.4500
55.6274
50.3992
31.8247
29.1217
A (d)
56.91
J(124.13)
B (d)
56.04
J(124.23)
f1 (ppm)

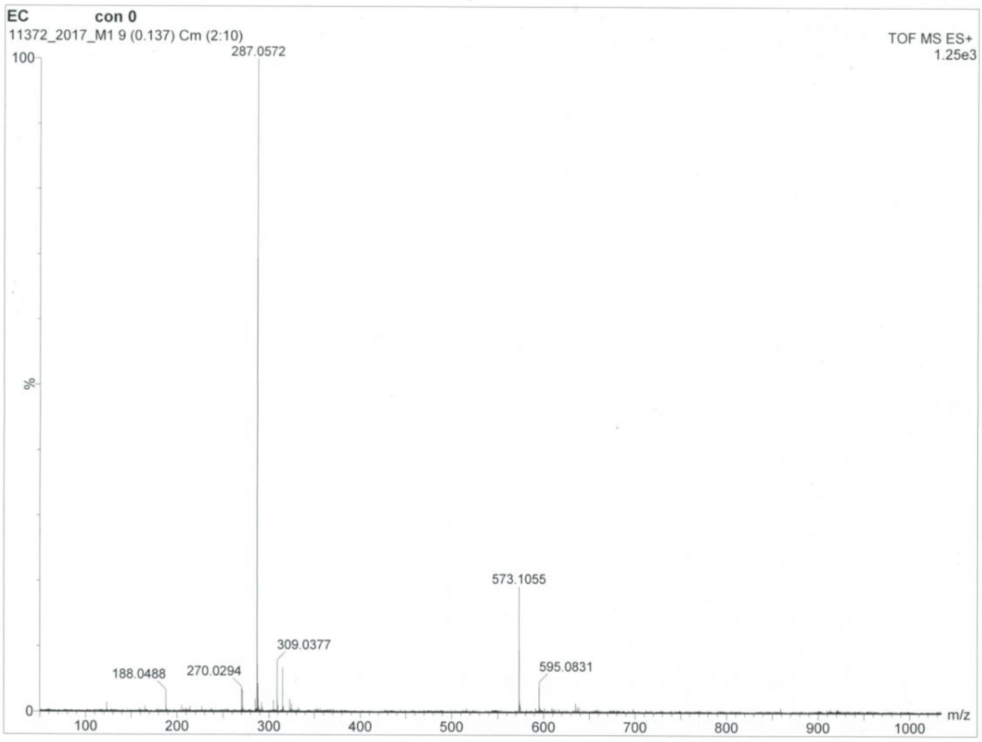
EC con 0
11372_2017_M1 9 (0.137) Cm (2:10)
TOF MS ES+
1.25e3
287.0572
573.1055
309.0377
595.0831
188.0488
270.0294
%
m/z

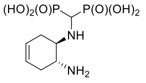

(1*R*,2*R*)- 4-Cyclohexene-1-amino-2-aminomethylenebisphosphonic acid [(1*R*,2*R*)-**10**]

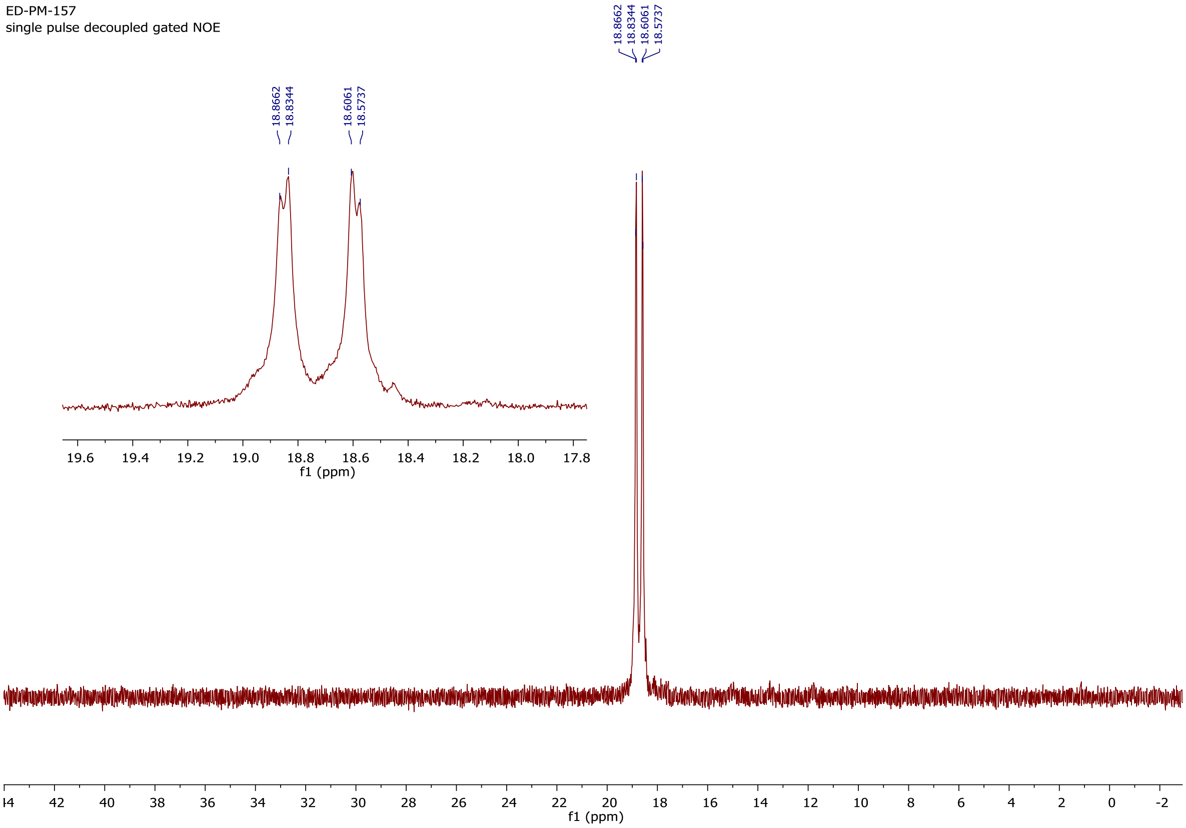

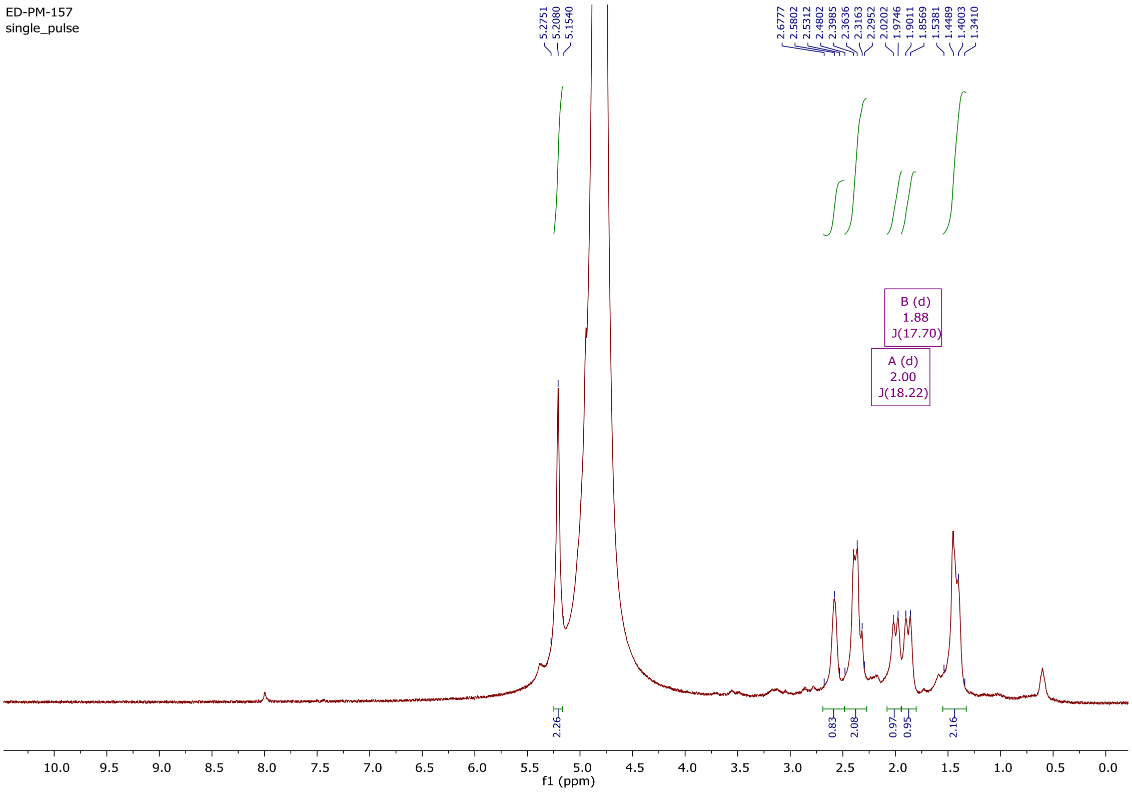

ED-PM-157
single pulse decoupled gated NOE

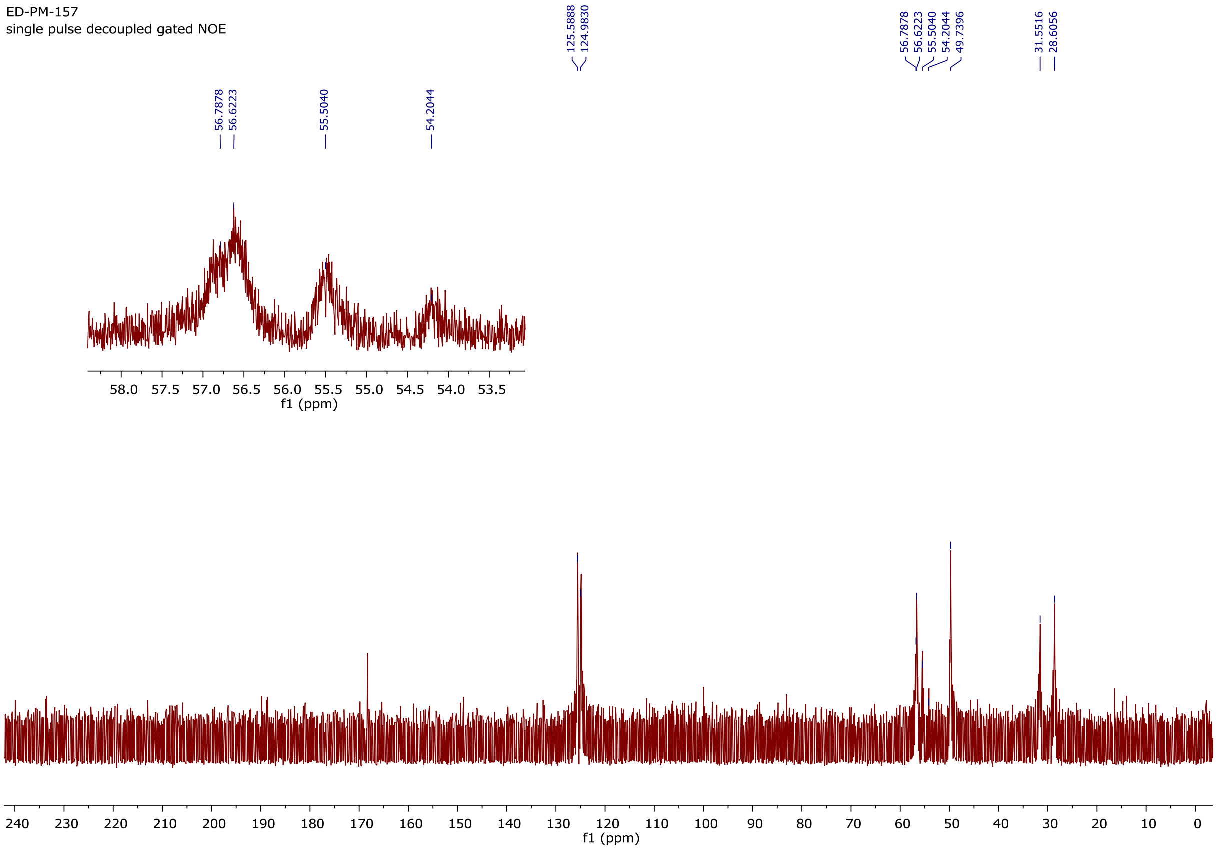

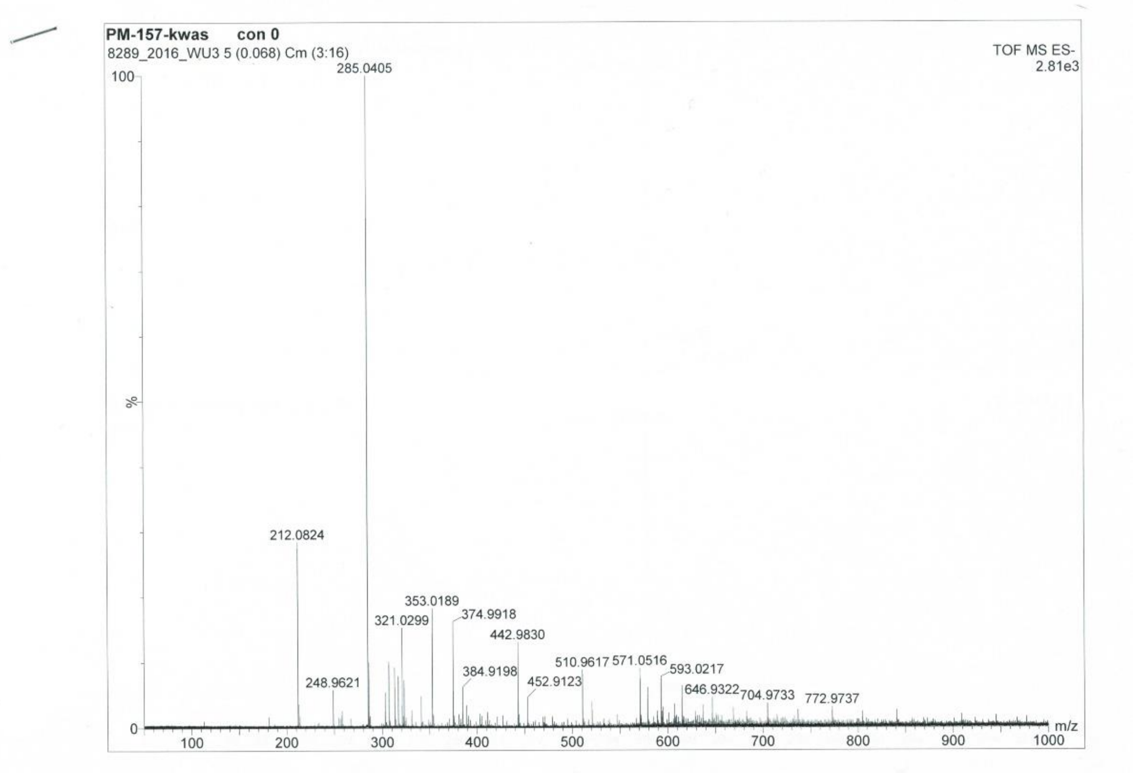

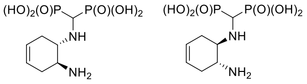

(±)-(trans)-4-Cyclohexene-1-amino-2-aminomethylenebisphosphonic acid [(*trans*)-**10**]

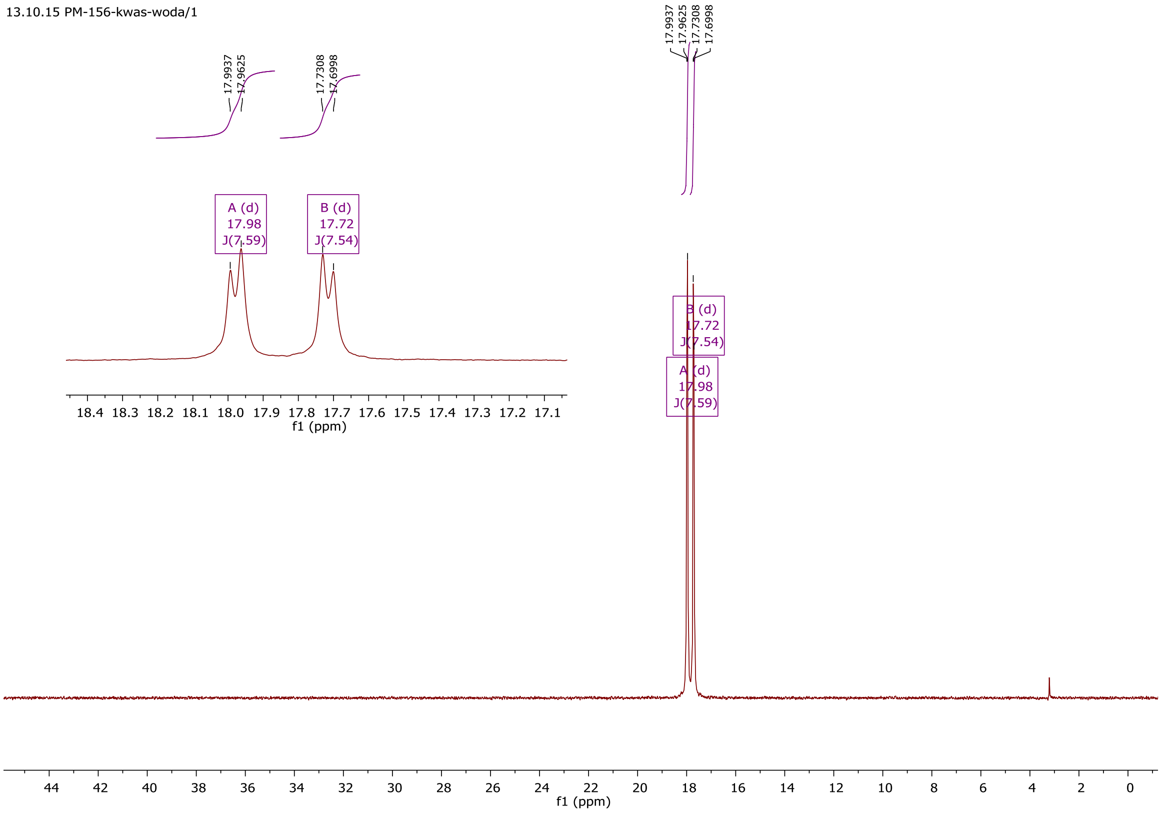

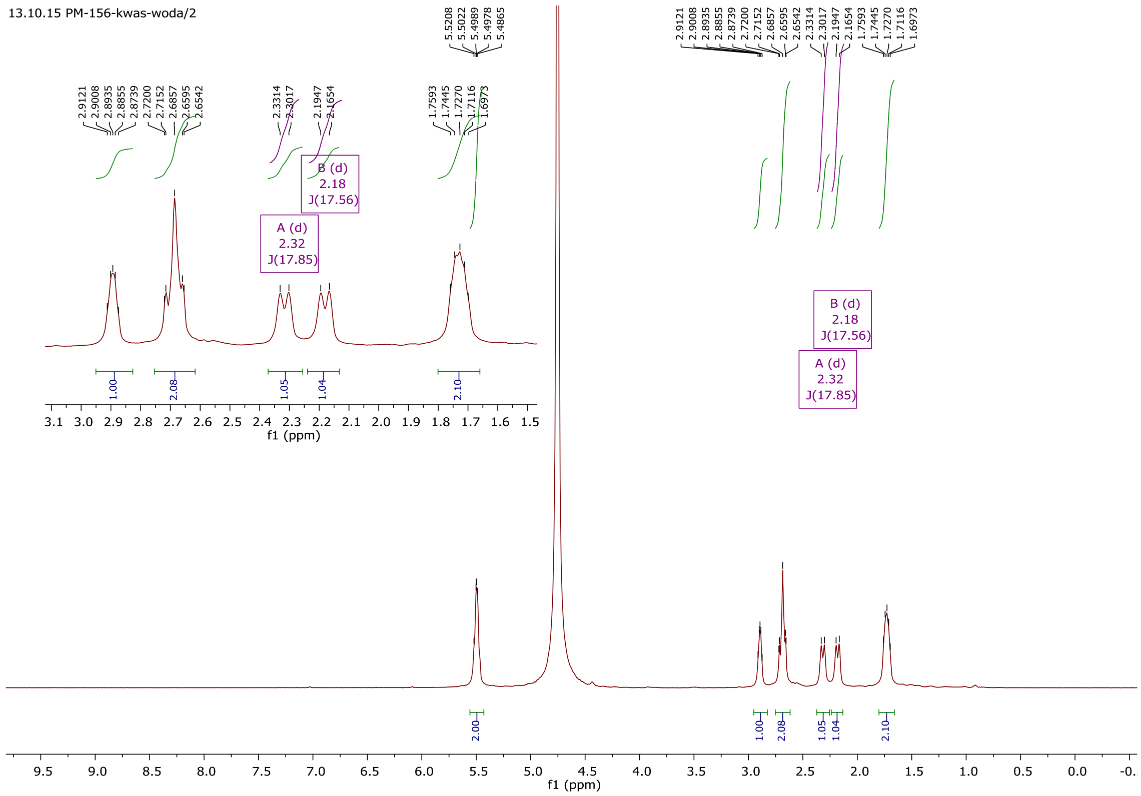

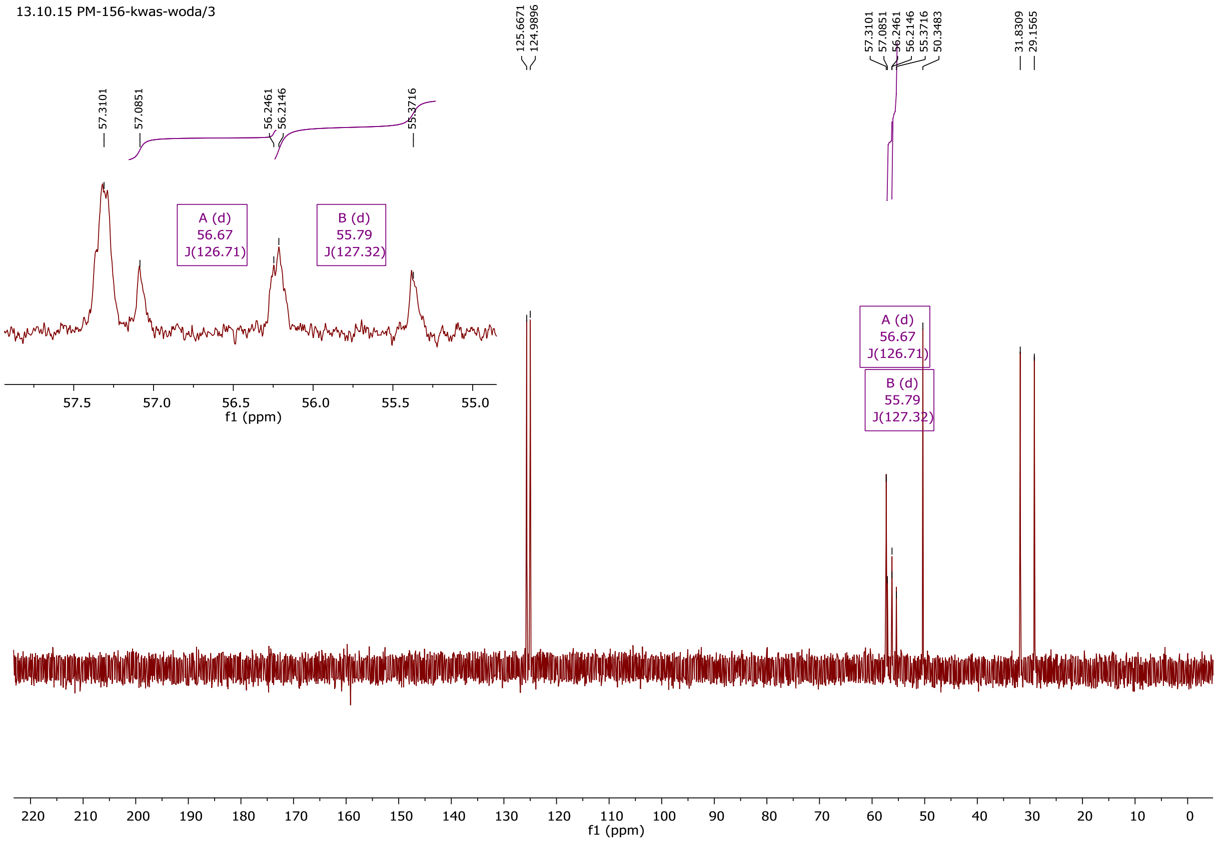
13.10.15 PM-156-kwas-woda/3
125.6671
124.9896
57.3101
57.0851
56.2461
56.2146
55.3716
50.3483
31.8309
29.1565
A (d)
56.67
J(126.71)
B (d)
55.79
J(127.32)
f1 (ppm)

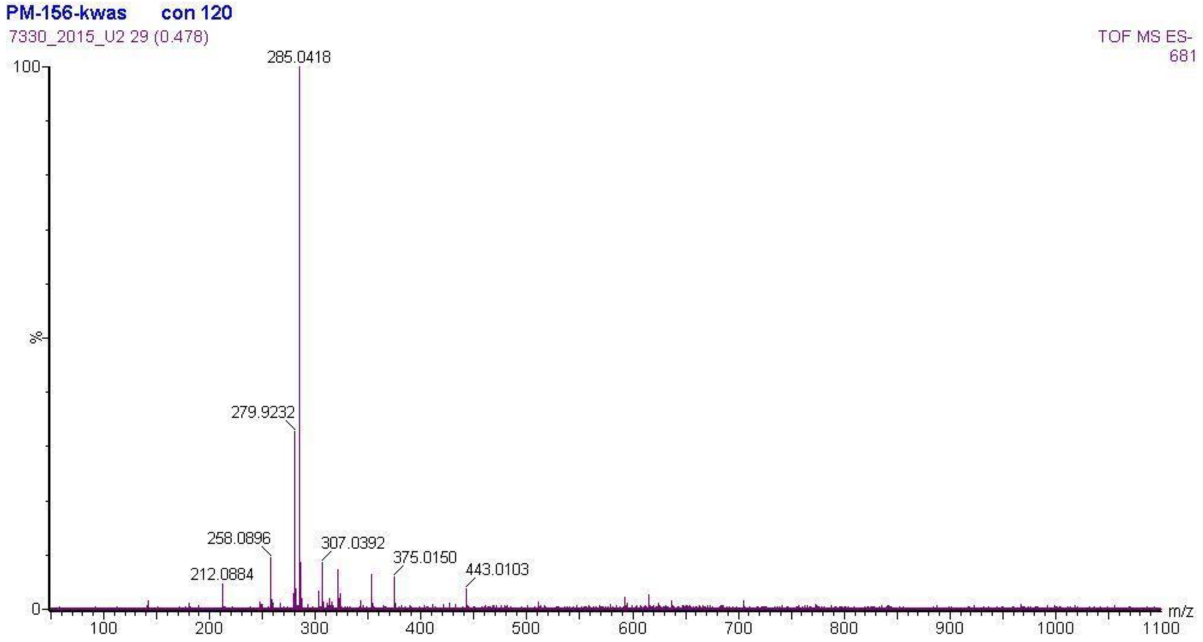
PM-156-kwas con 120
7330_2015_U2 29 (0.478)
TOF MS ES-
681
285.0418
279.9232
258.0896
307.0392
212.0884
375.0150
443.0103
%
m/z

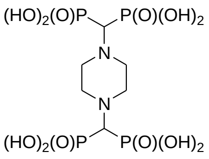

Piperaz-1,4-diylmethylenebisphosphonic acid (**11**)

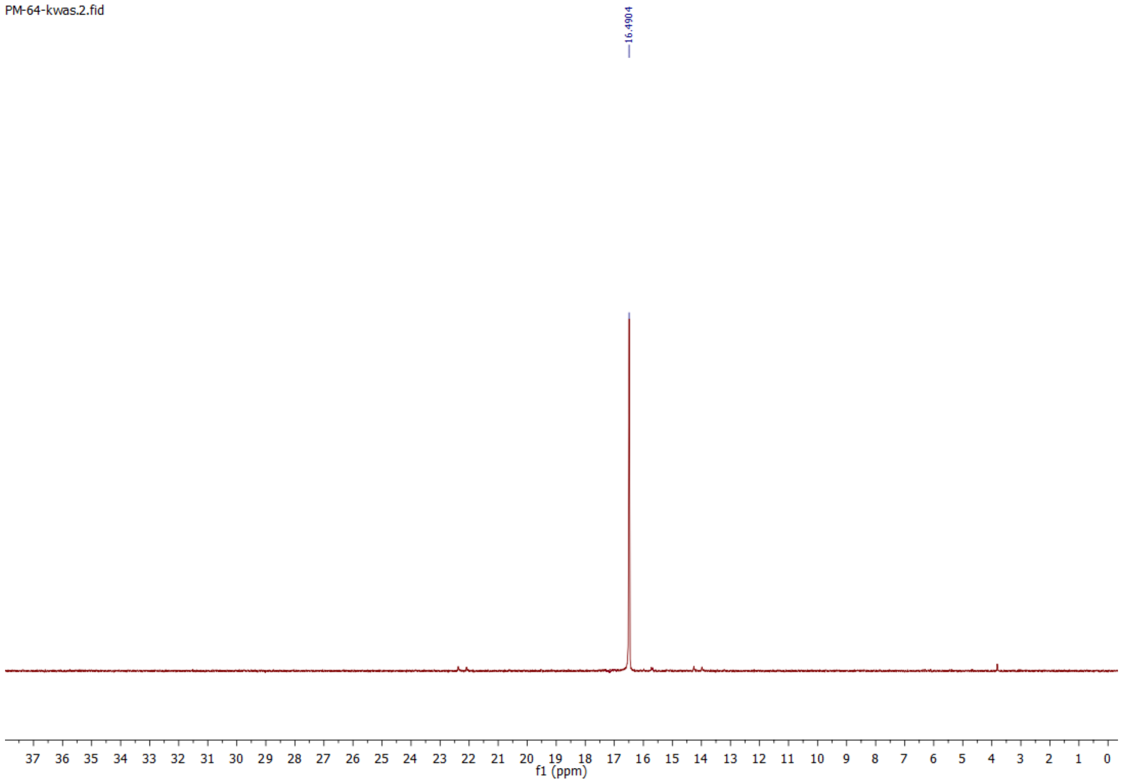

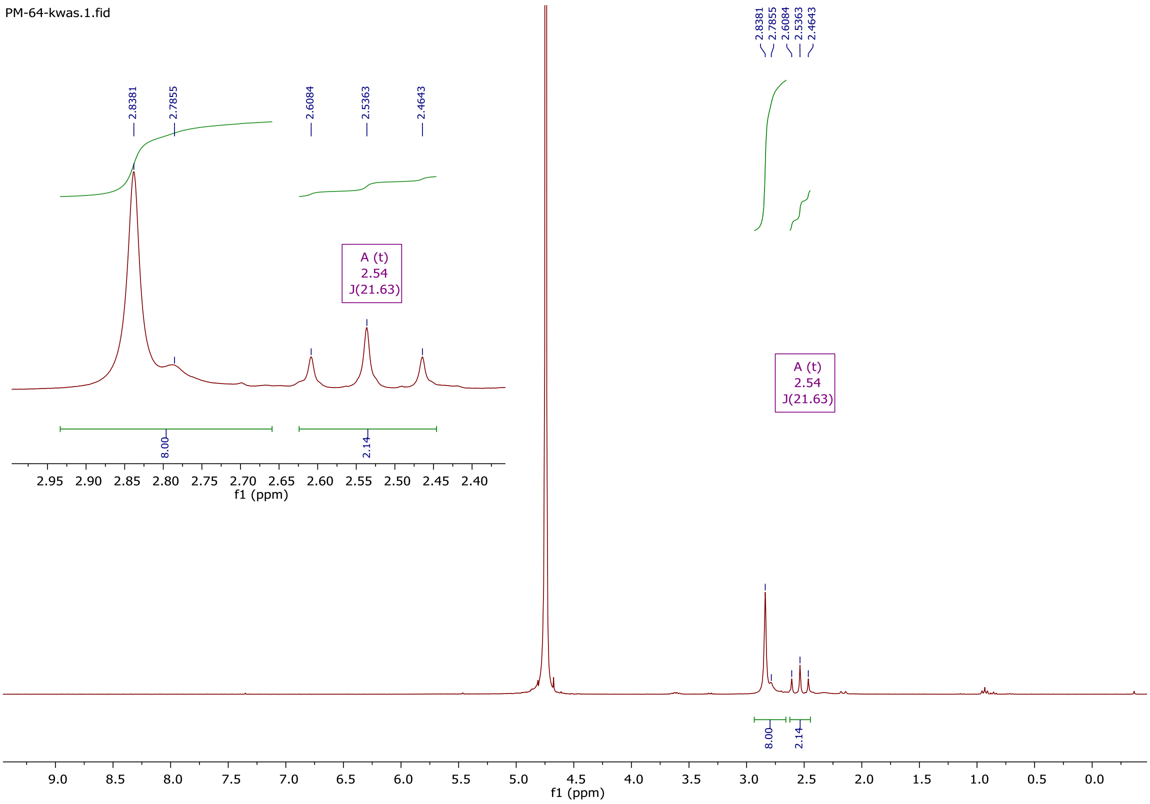

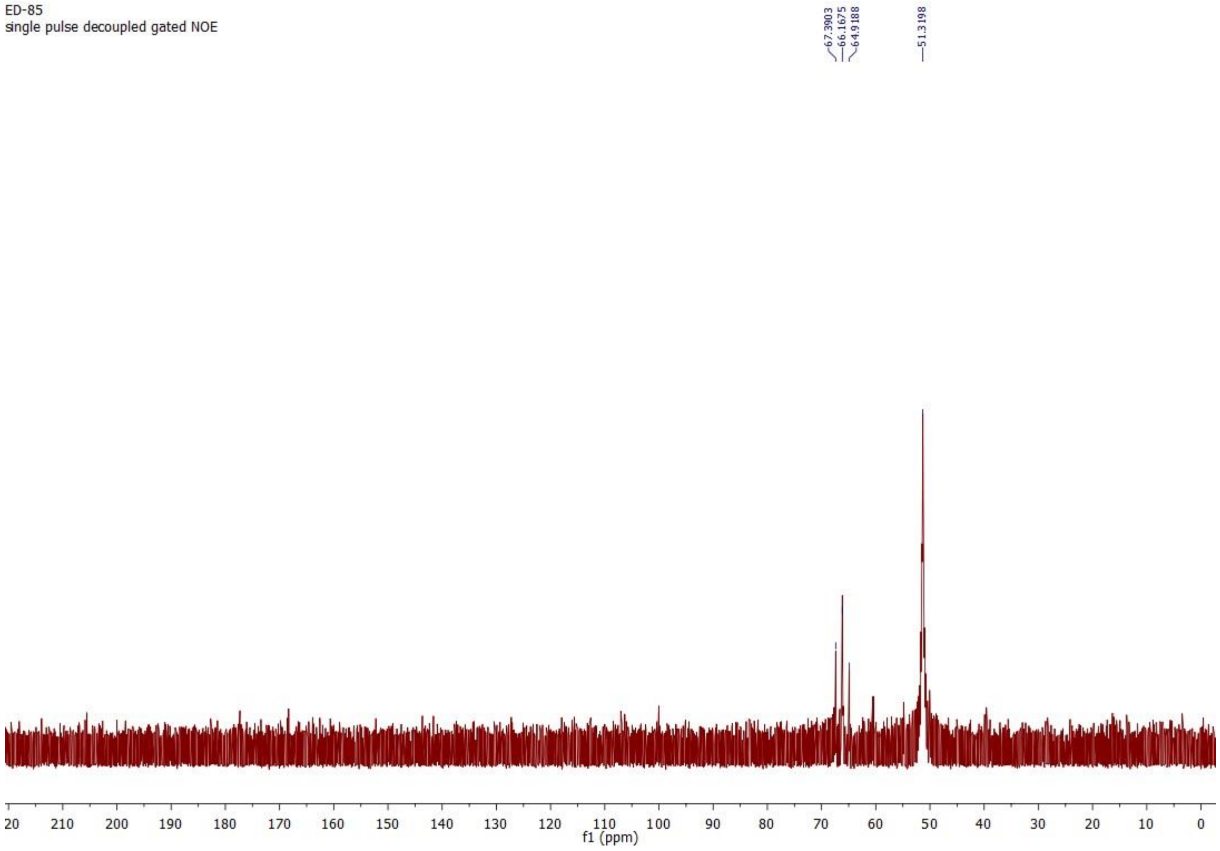
ED-85
single pulse decoupled gated NOE
67.3903
66.1675
64.9188
51.3198
f1 (ppm)

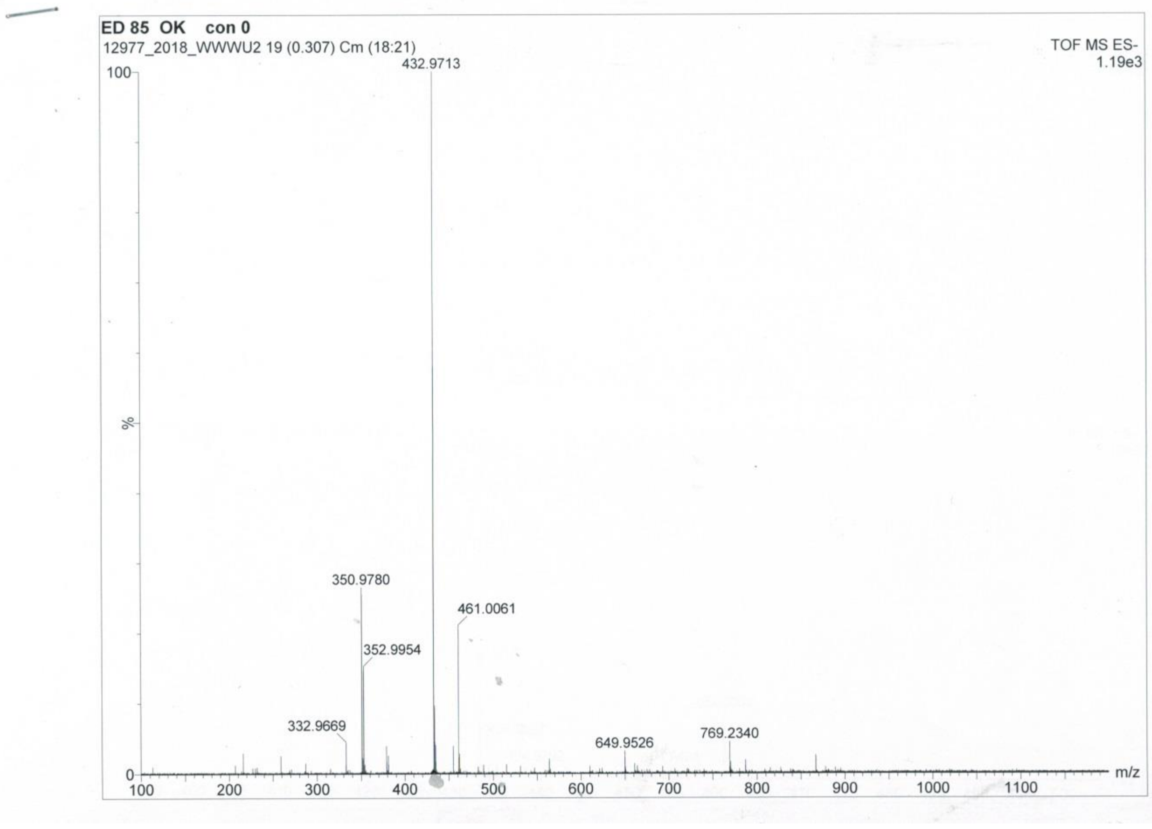
ED 85 OK con 0
12977_2018_WWWU2 19 (0.307) Cm (18:21)
TOF MS ES-
1.19e3
432.9713
350.9780
461.0061
352.9954
332.9669
649.9526
769.2340
%
m/z

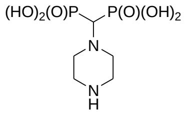

Piperaz-1-ylmethylenebisphosphonic acid (**12**)

EC-15-s-06-FC-205
single pulse decoupled gated NOE

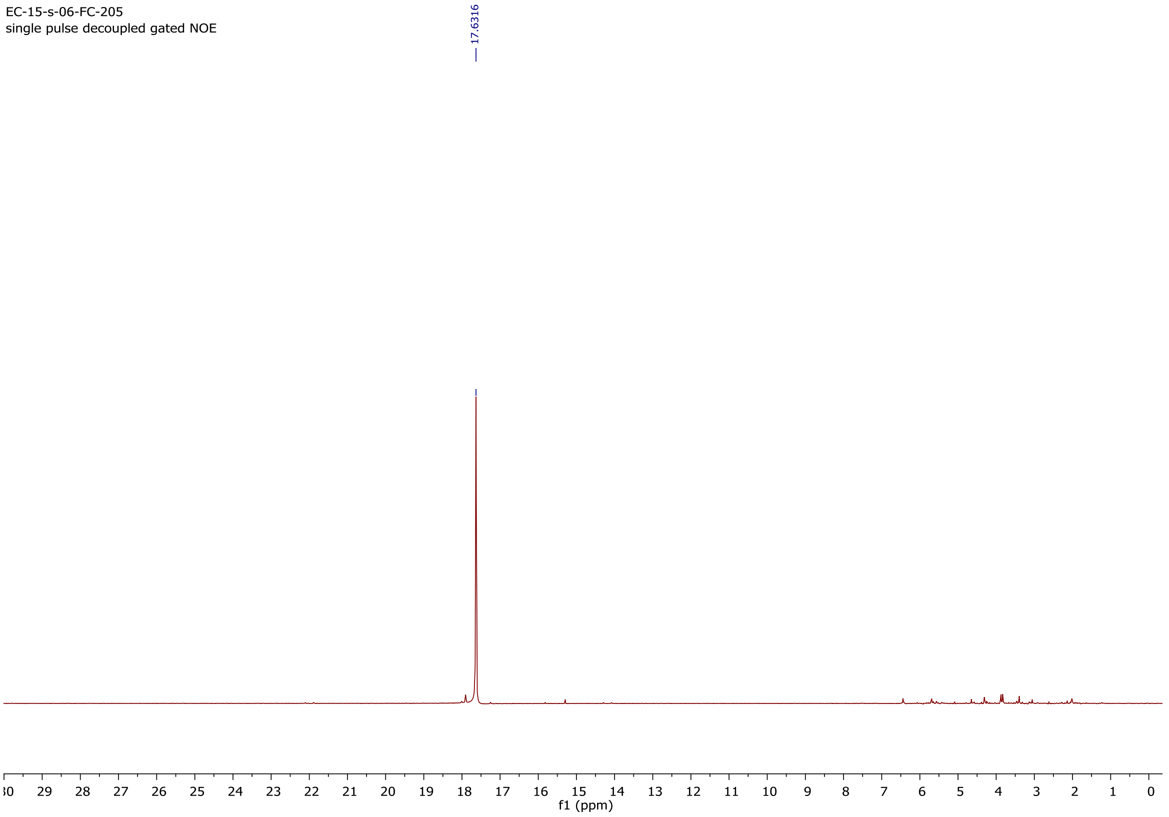

EC-15-s-06-FC-205
single_pulse

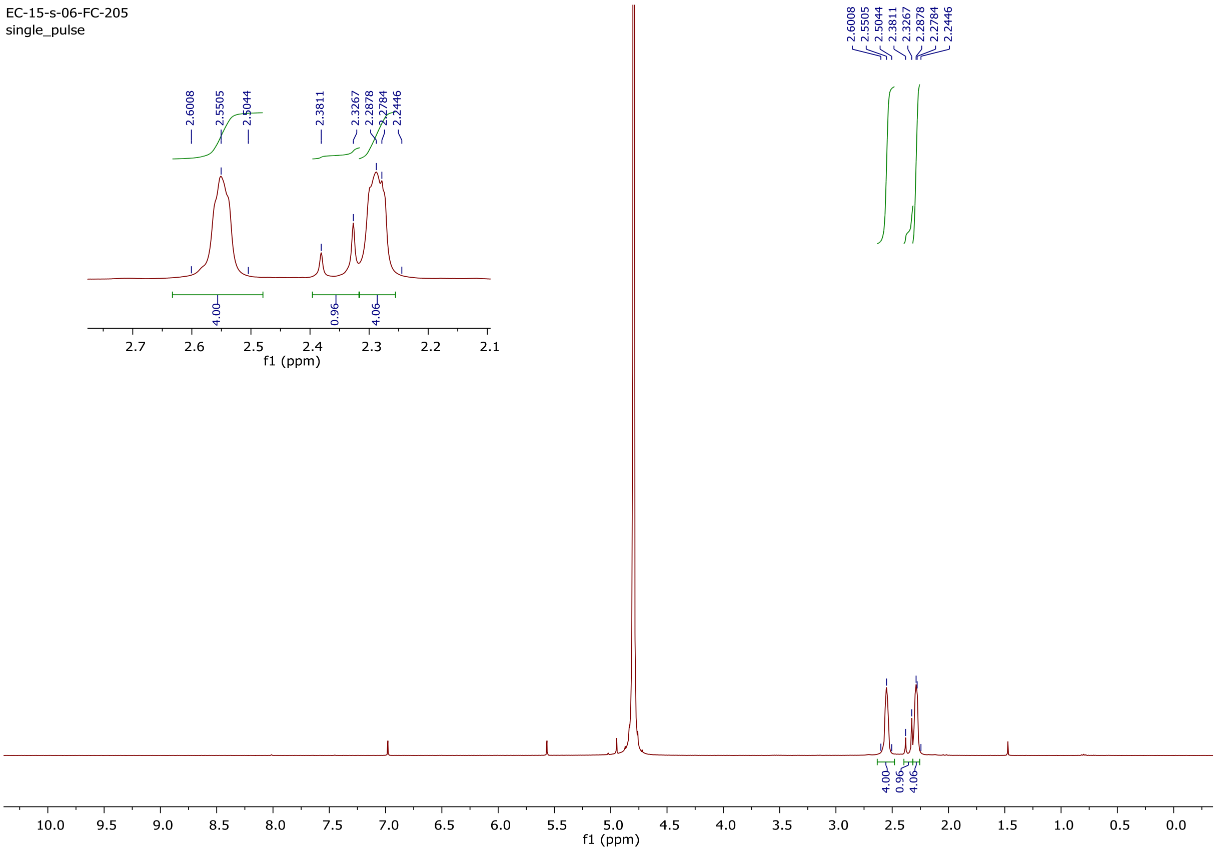

EC-15-s-06-FC-205
single pulse decoupled gated NOE

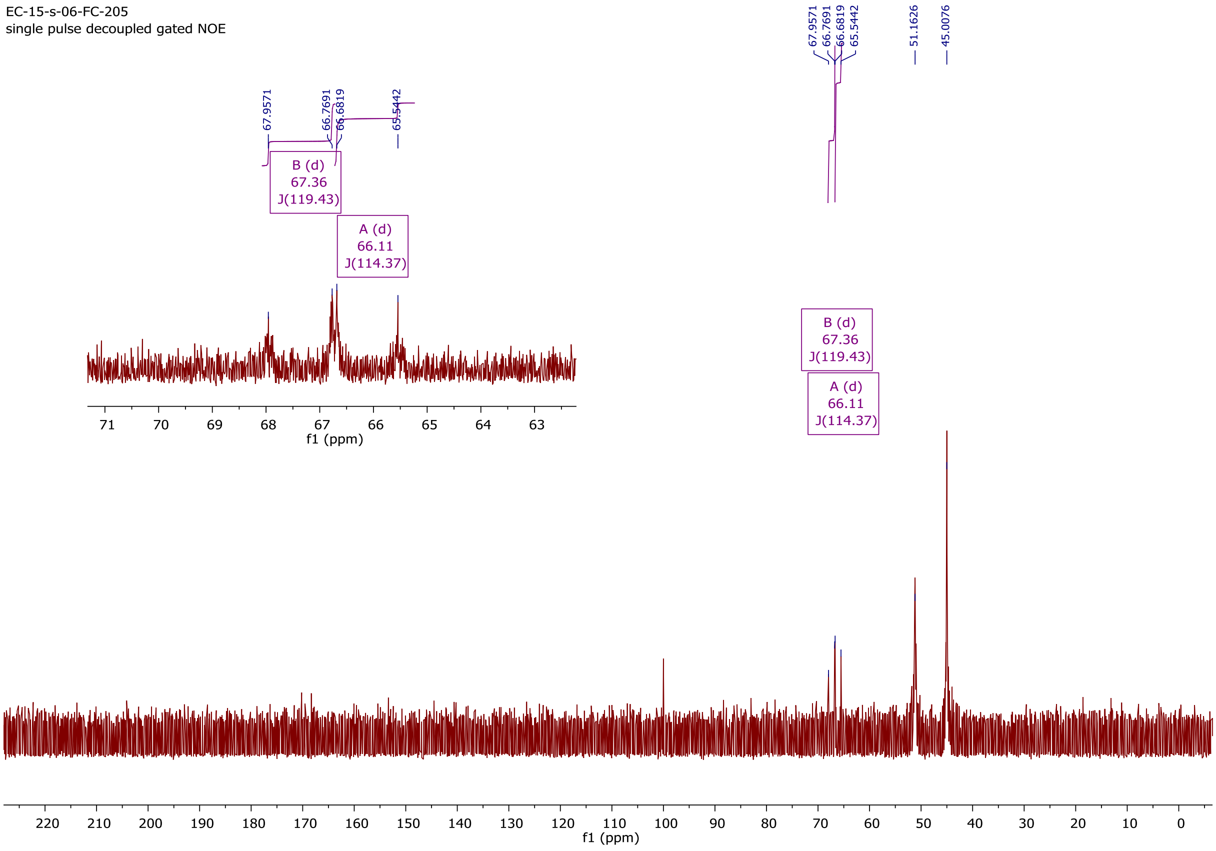

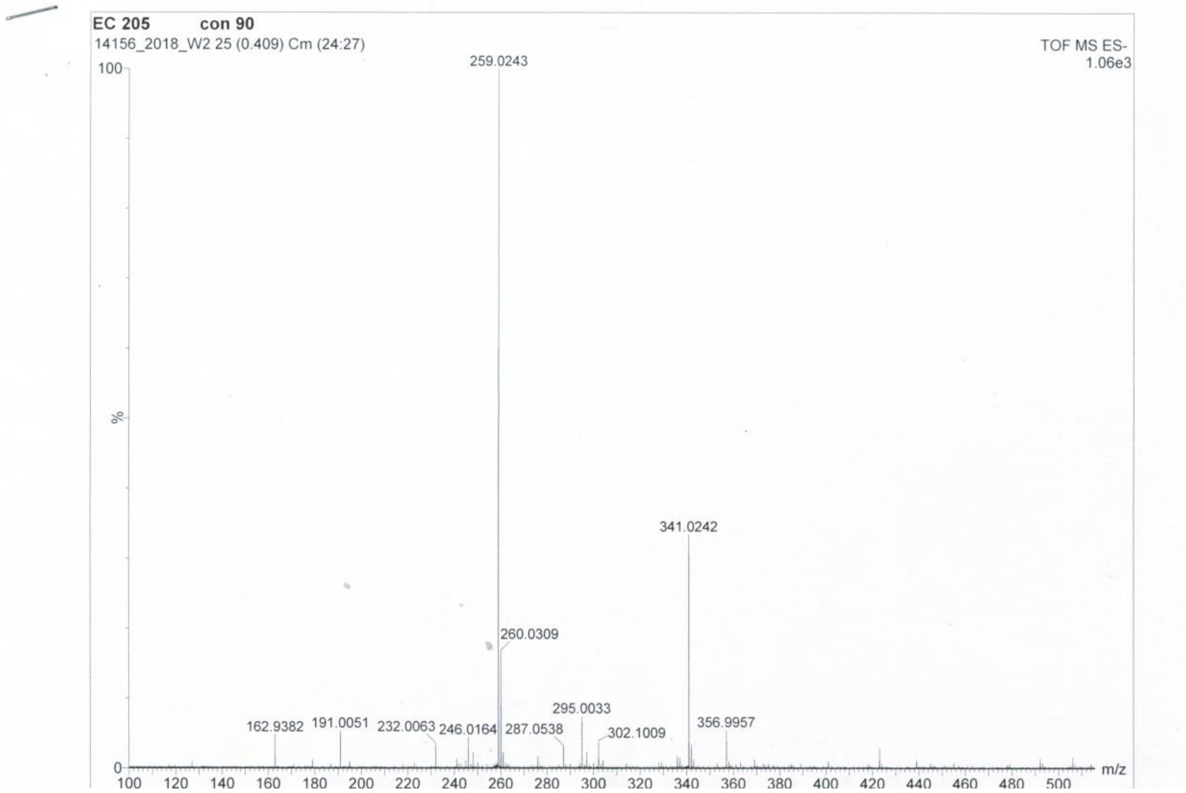

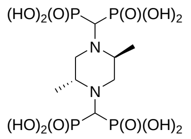

2,5-trans-Dimethylpiperaz-1,4-diylmethylenebisphosphonic acid [(*trans*)-**13]**

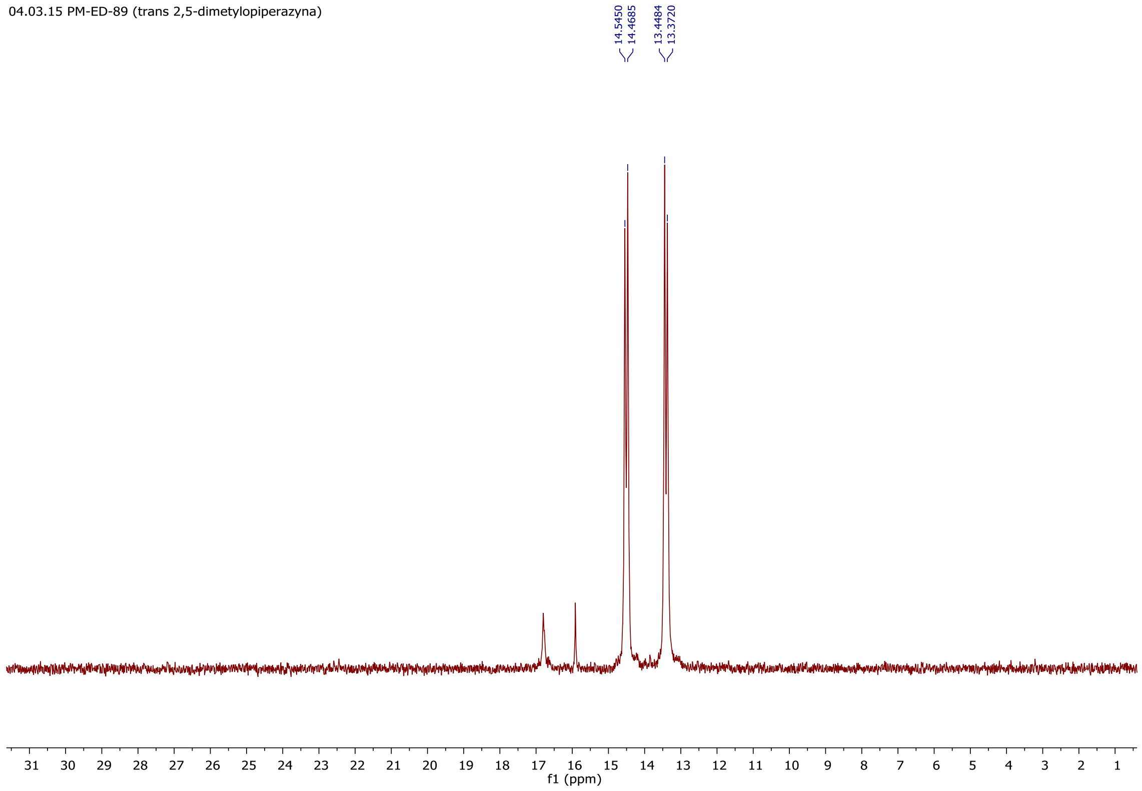

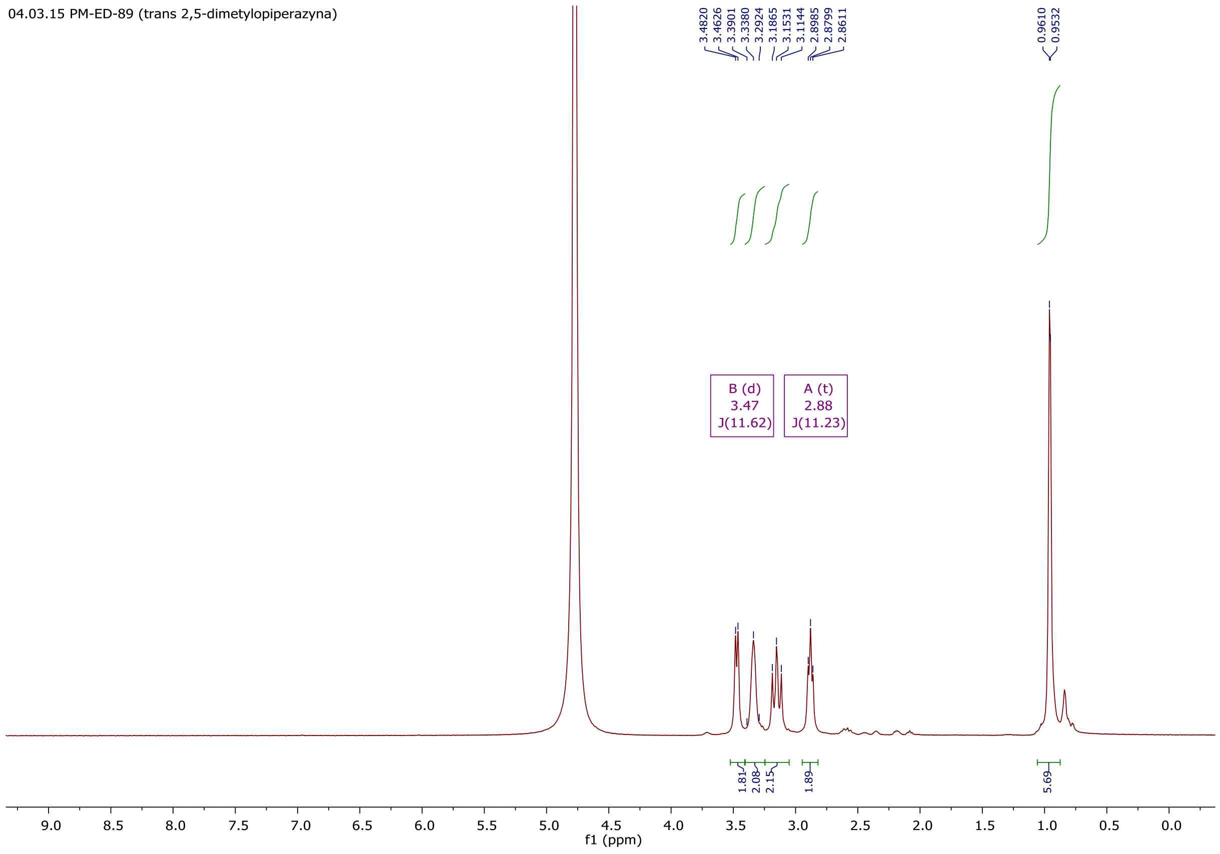

03.03.15 PM-ED-89-13C (trans 2,5-dimetylopiperazyna)

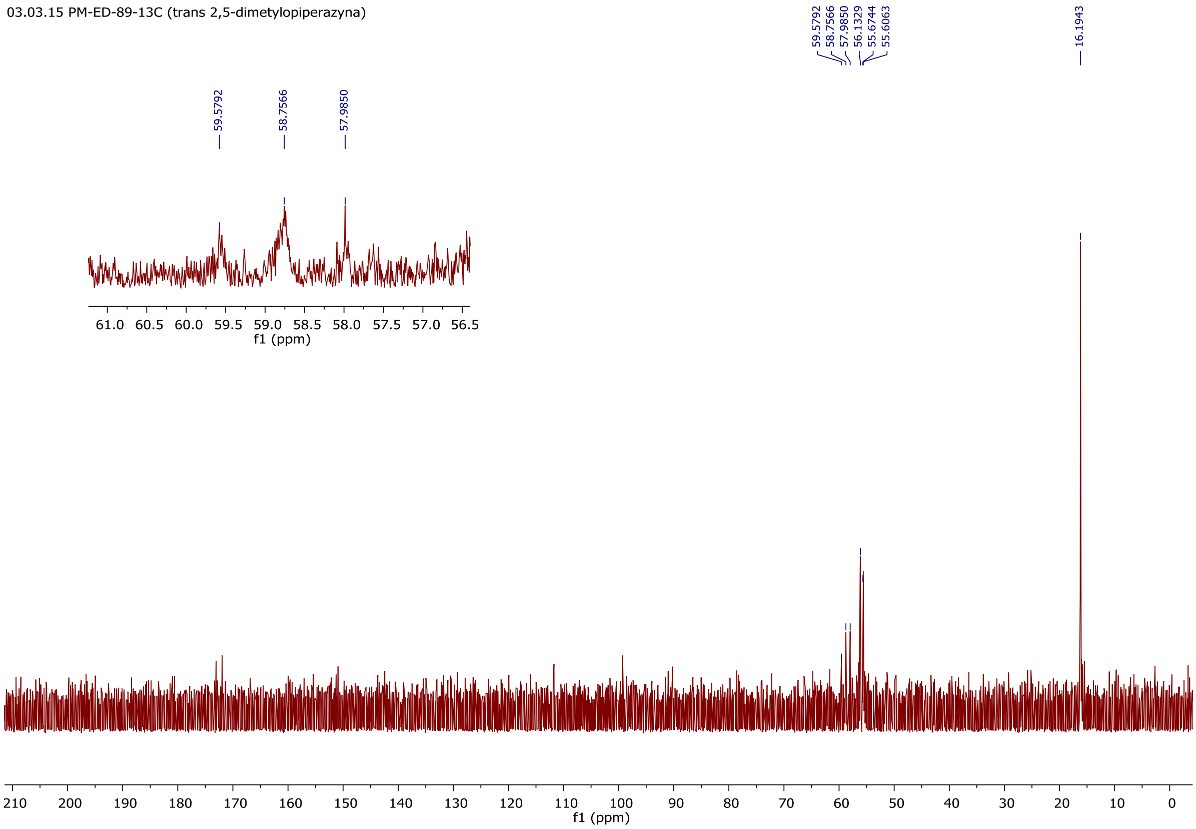

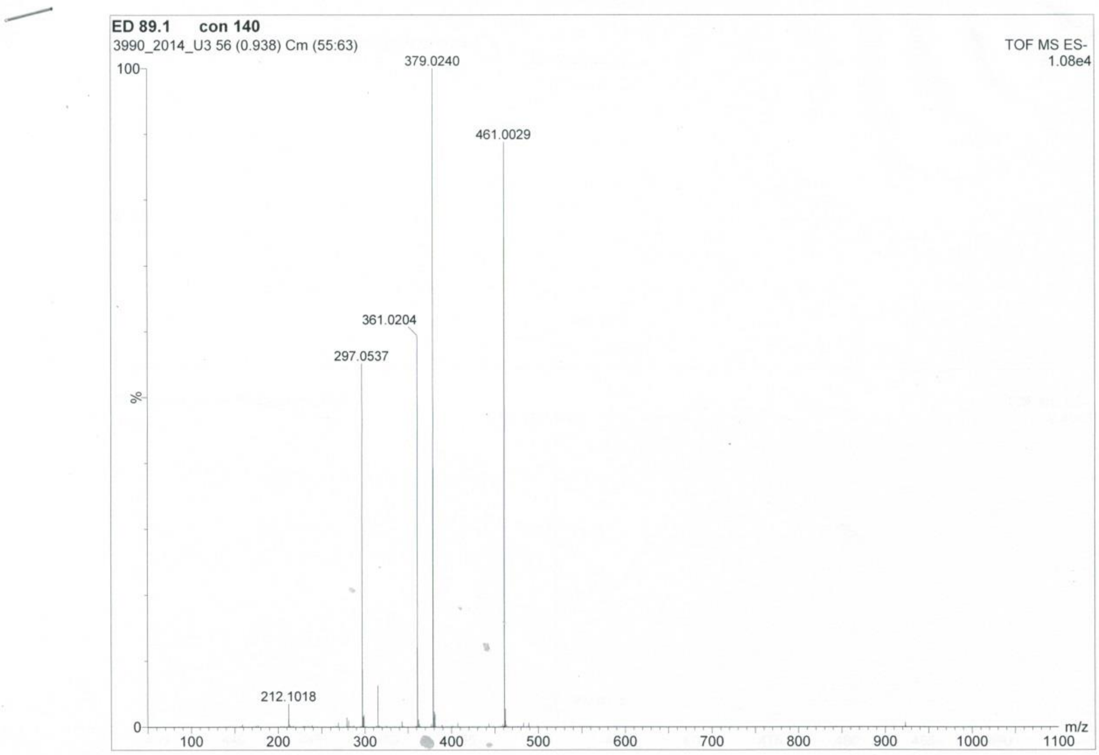

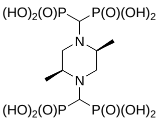

2,5-cis-Dimethylpiperaz-1,4-diylmethylenebisphosphonic acid [(*cis*)-**13]**

15-500JS004
user ec
31P NMR, PM-EC-116W, 15-0442
P3164 D2O {C:\-\js} nmrsu 8

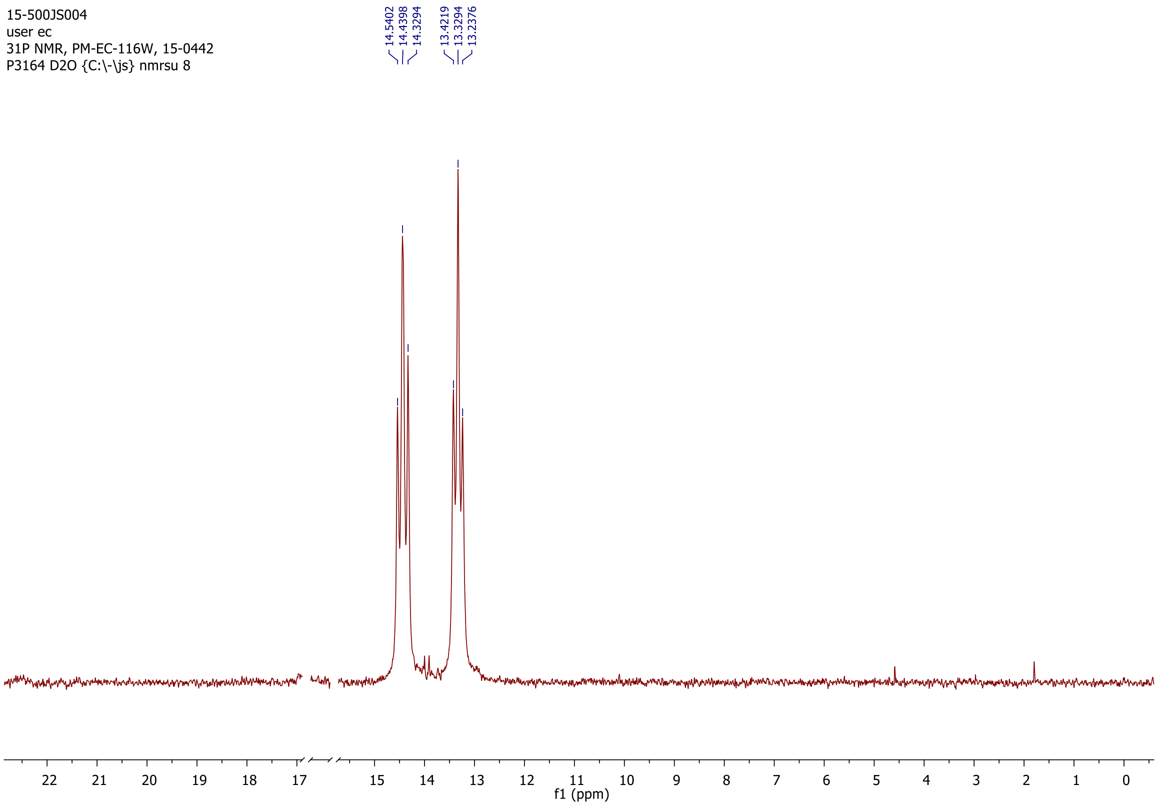

15-500JS004.1.fid
user ec
1H NMR, PM-EC-116W, 15-0442
PROTON1m D2O {C:\-\js} nmrsu 8

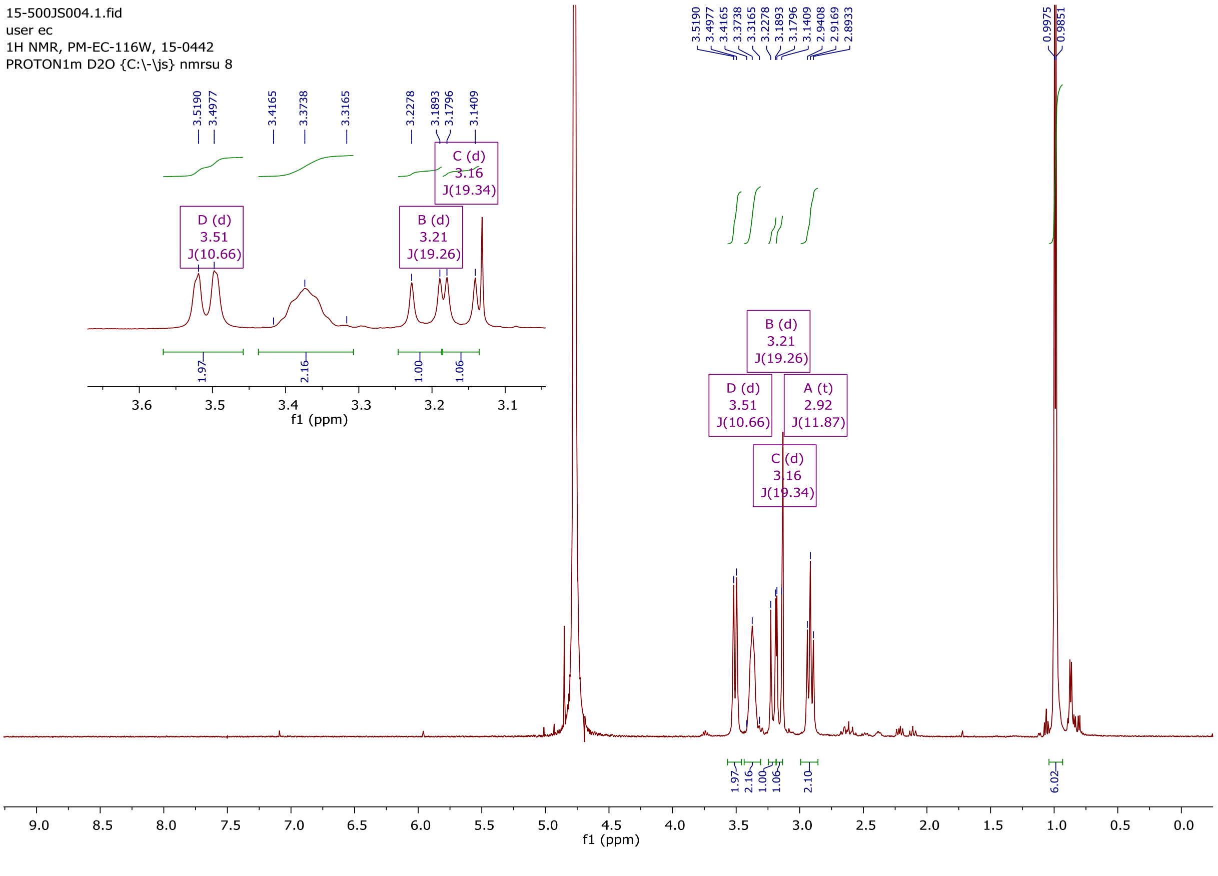

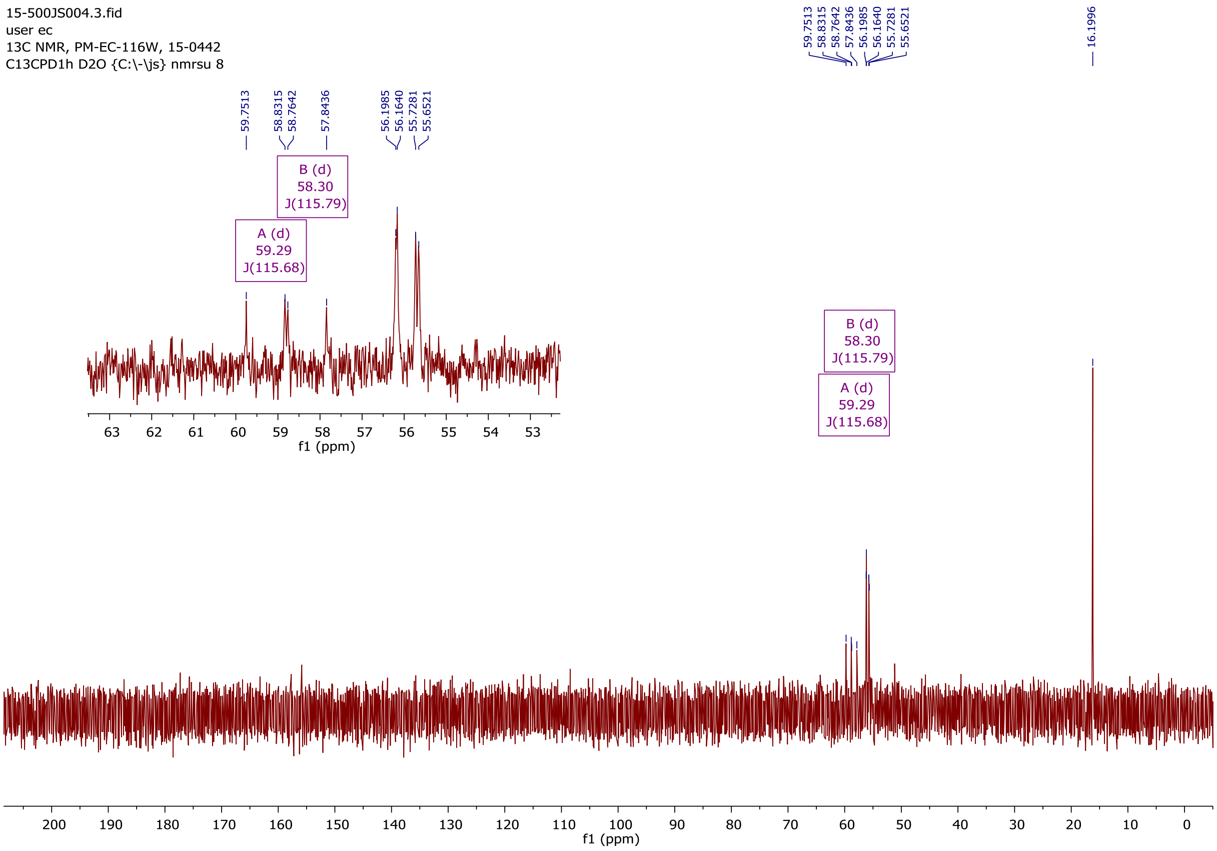
15-500JS004.3.fid
user ec
13C NMR, PM-EC-116W, 15-0442
C13CPD1h D2O {C:\-\js} nmrsu 8
59.7513
58.8315
58.7642
57.8436
56.1985
56.1640
55.7281
55.6521
16.1996
B (d)
58.30
J(115.79)
A (d)
59.29
J(115.68)
f1 (ppm)

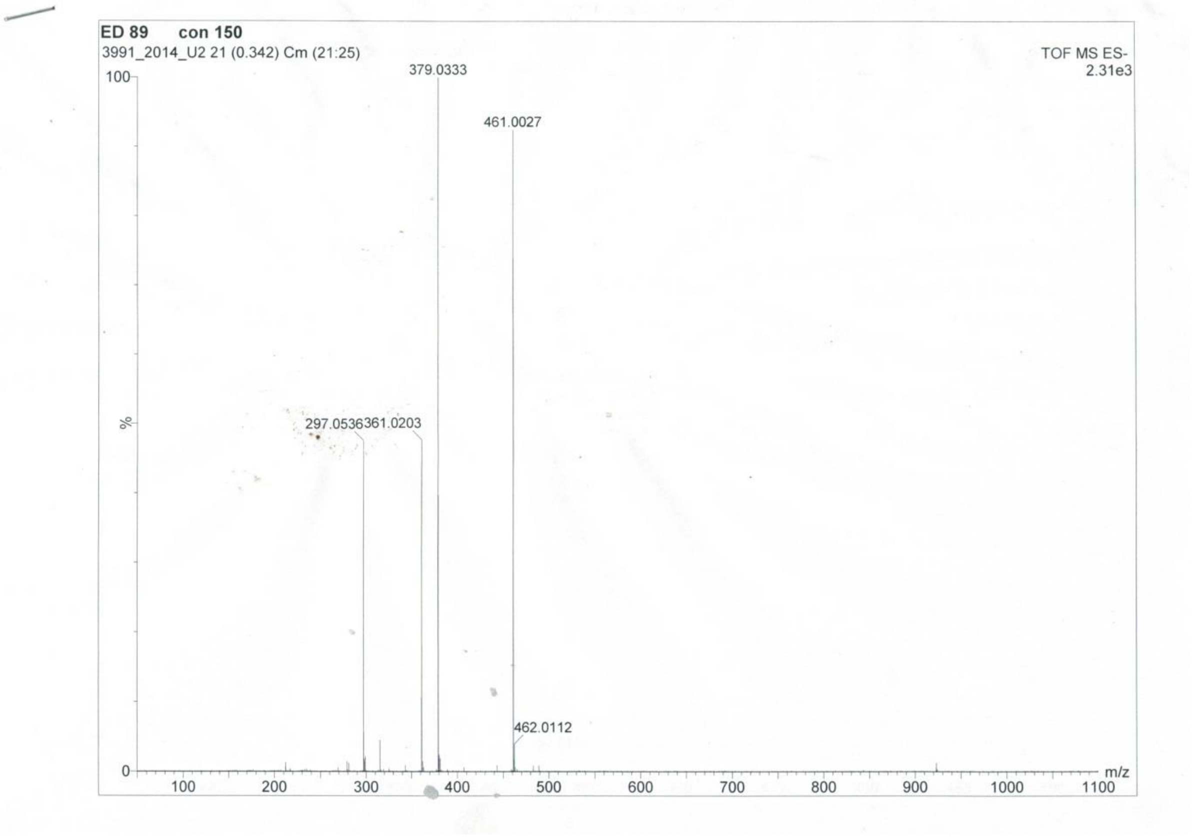
ED 89 con 150
3991_2014_U2 21 (0.342) Cm (21:25)
TOF MS ES-
2.31e3
379.0333
461.0027
297.0536
361.0203
462.0112
%
m/z

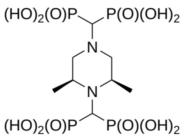

2,6-cis-Dimethylpiperaz-1,4-diylmethylenebisphosphonic acid [(*cis*)-**14**]

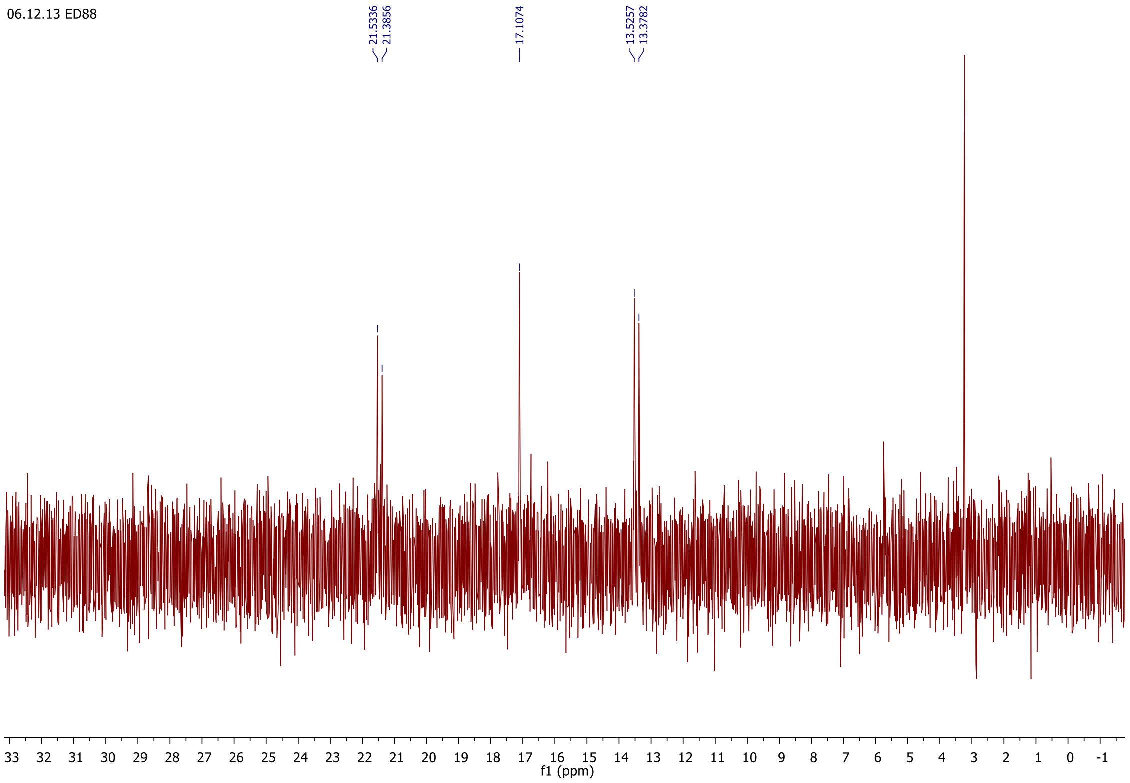

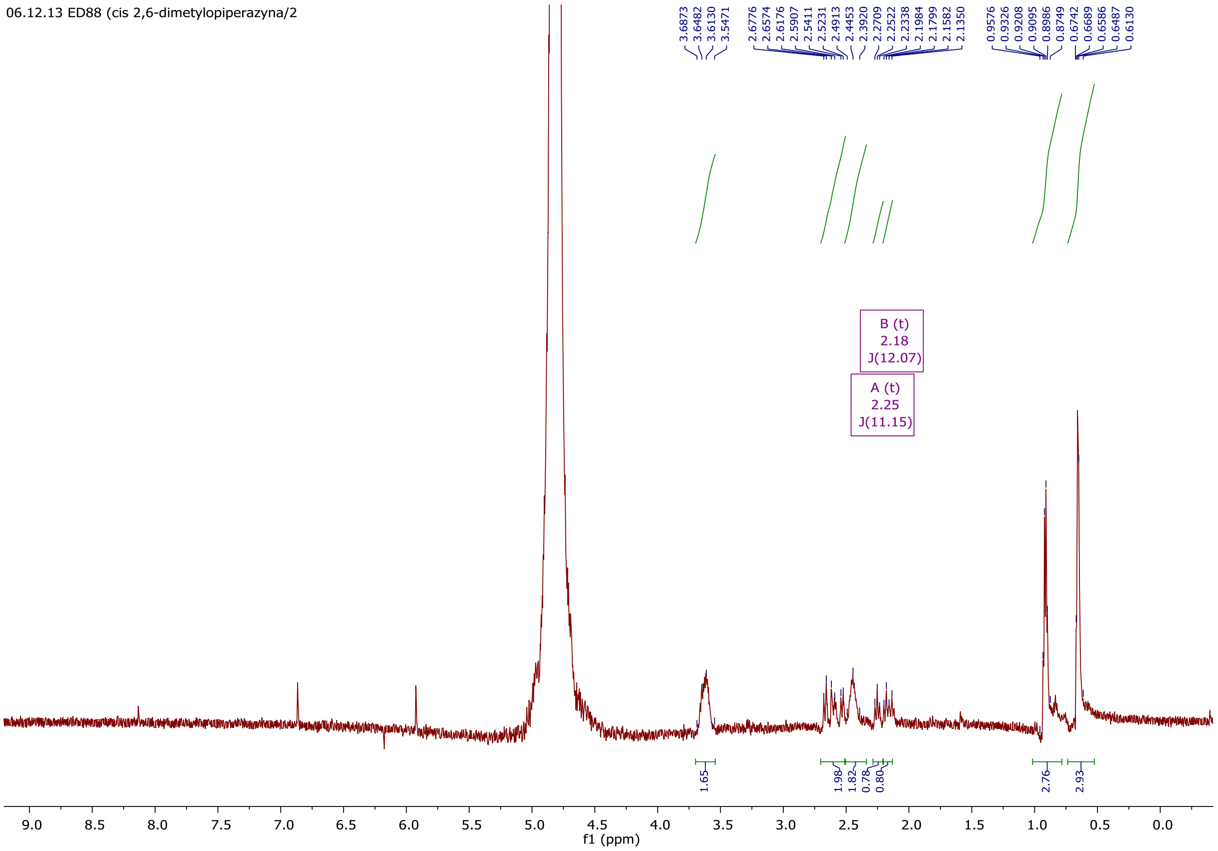

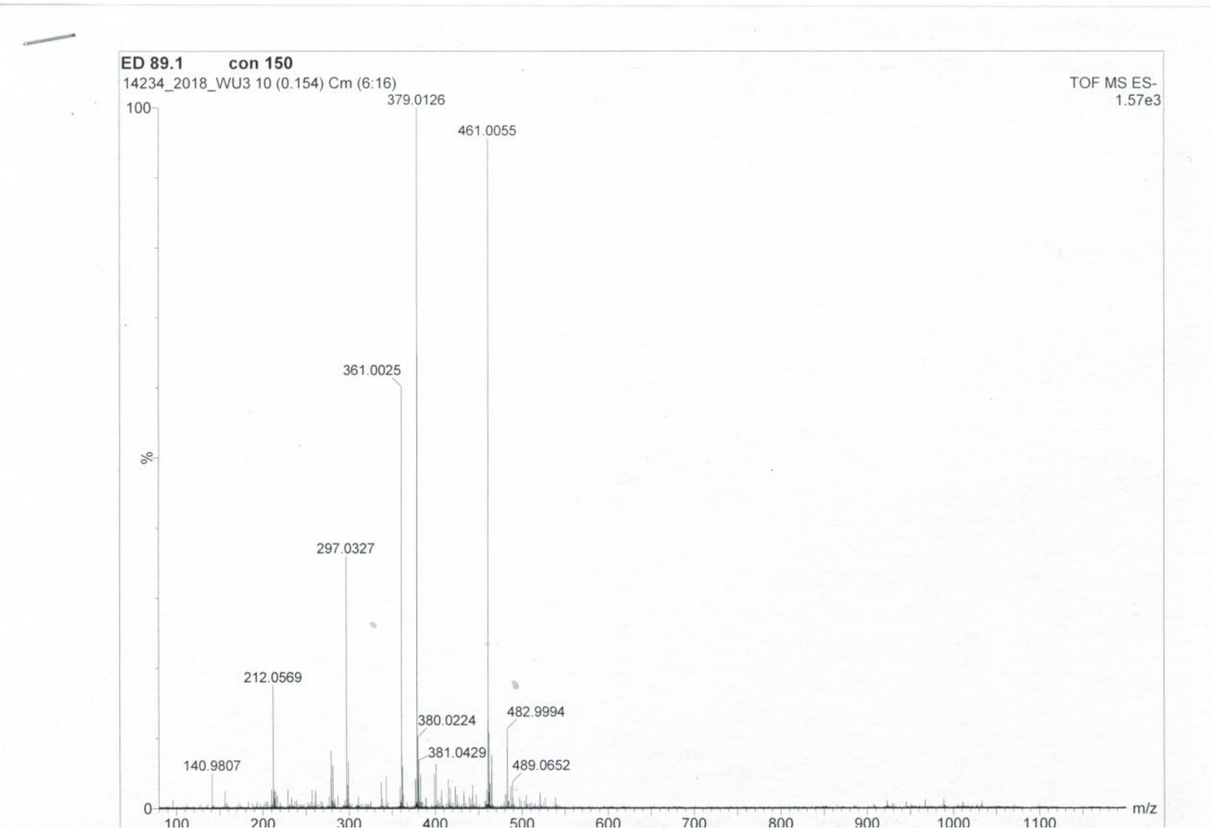

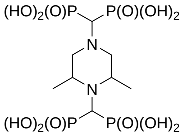

2,6-Dimethylpiperaz-1,4-diylmethylenebisphosphonic acid [(*rac*)-**14]**

ED-87-II
single pulse decoupled gated NOE

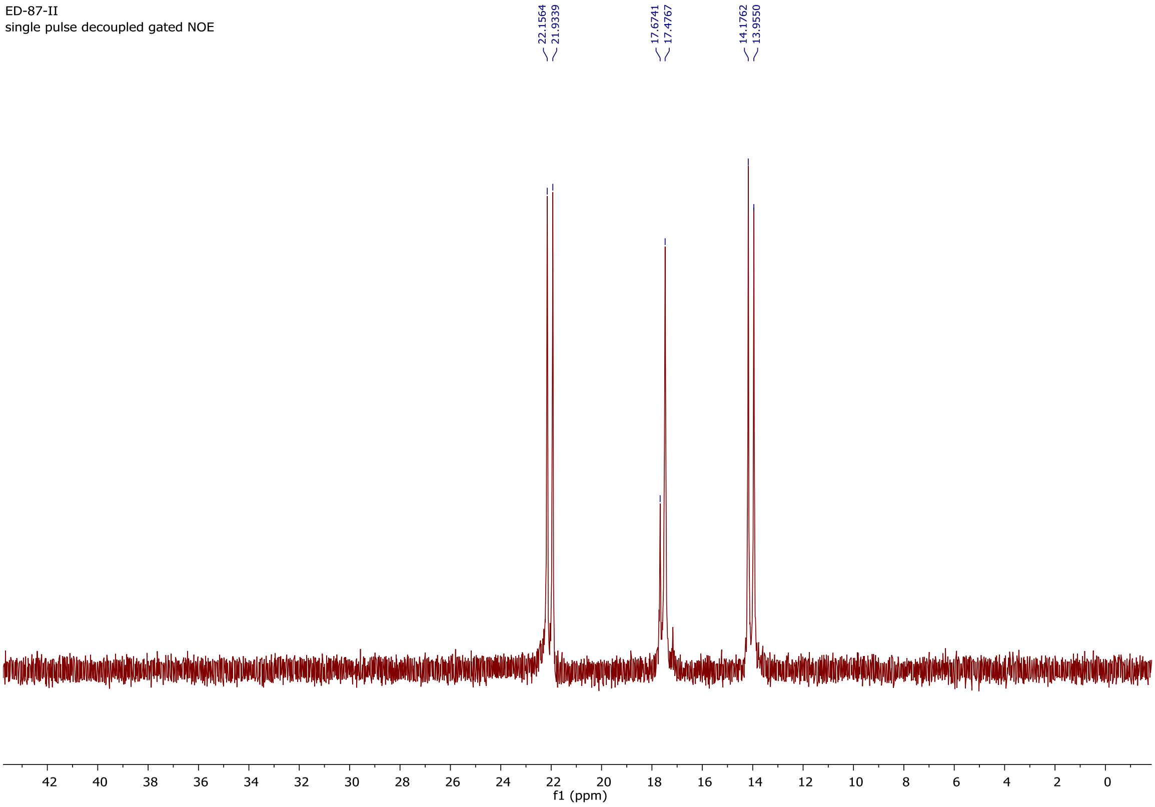

ED-87-II
single_pulse

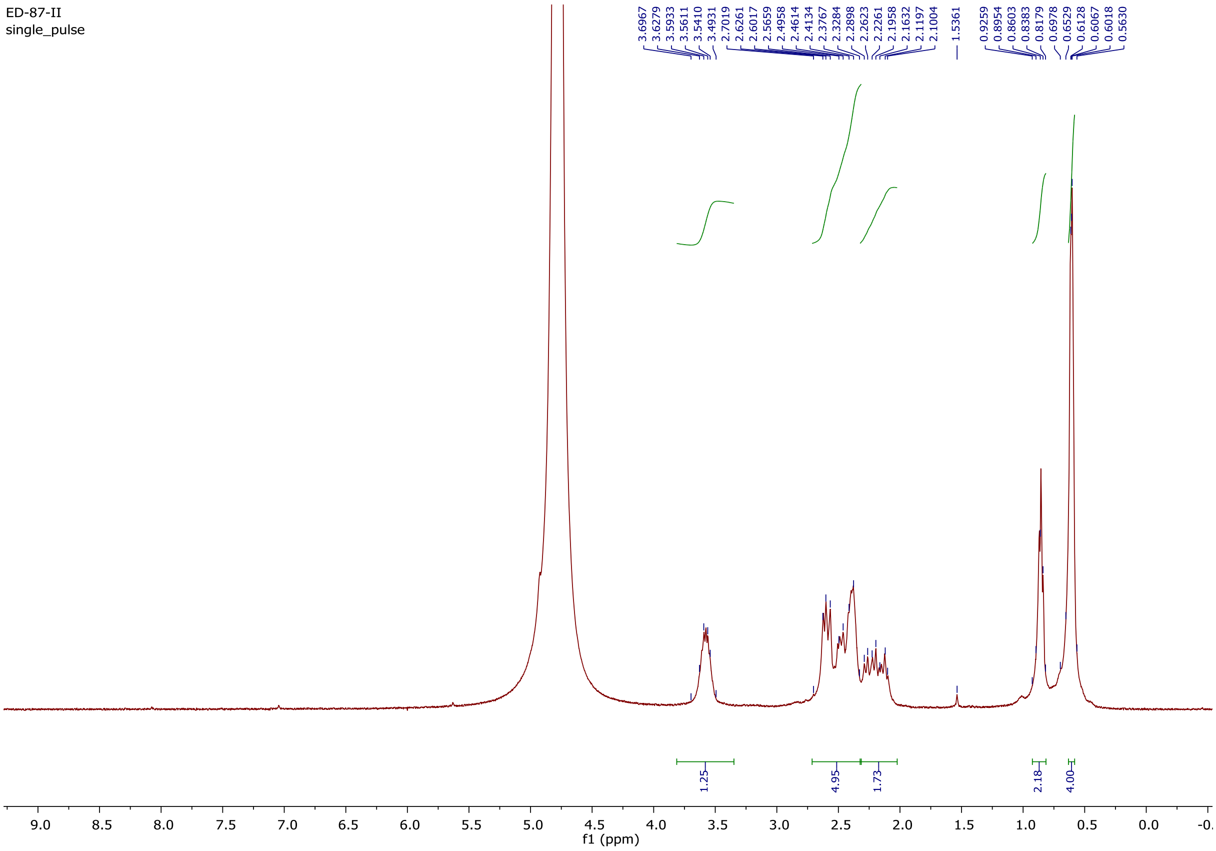

ED-87-II
single pulse decoupled gated NOE

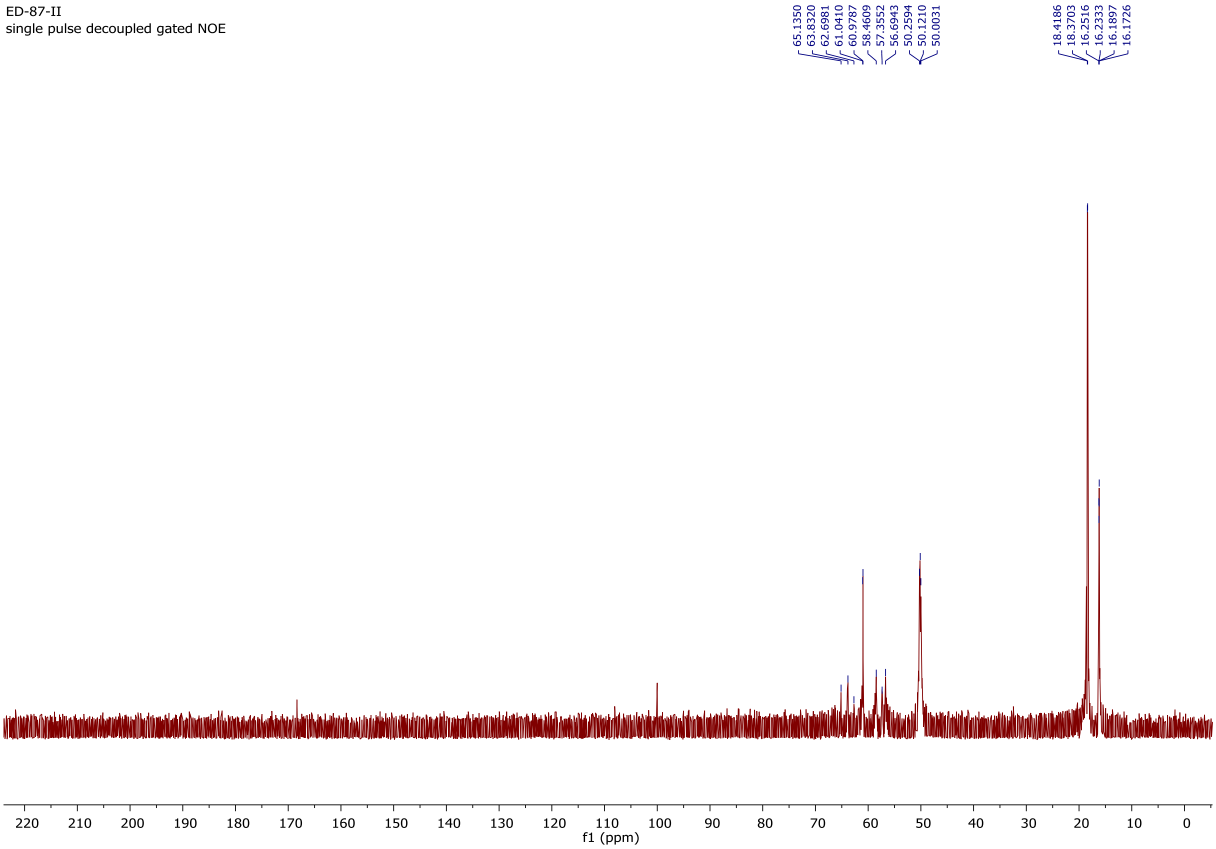

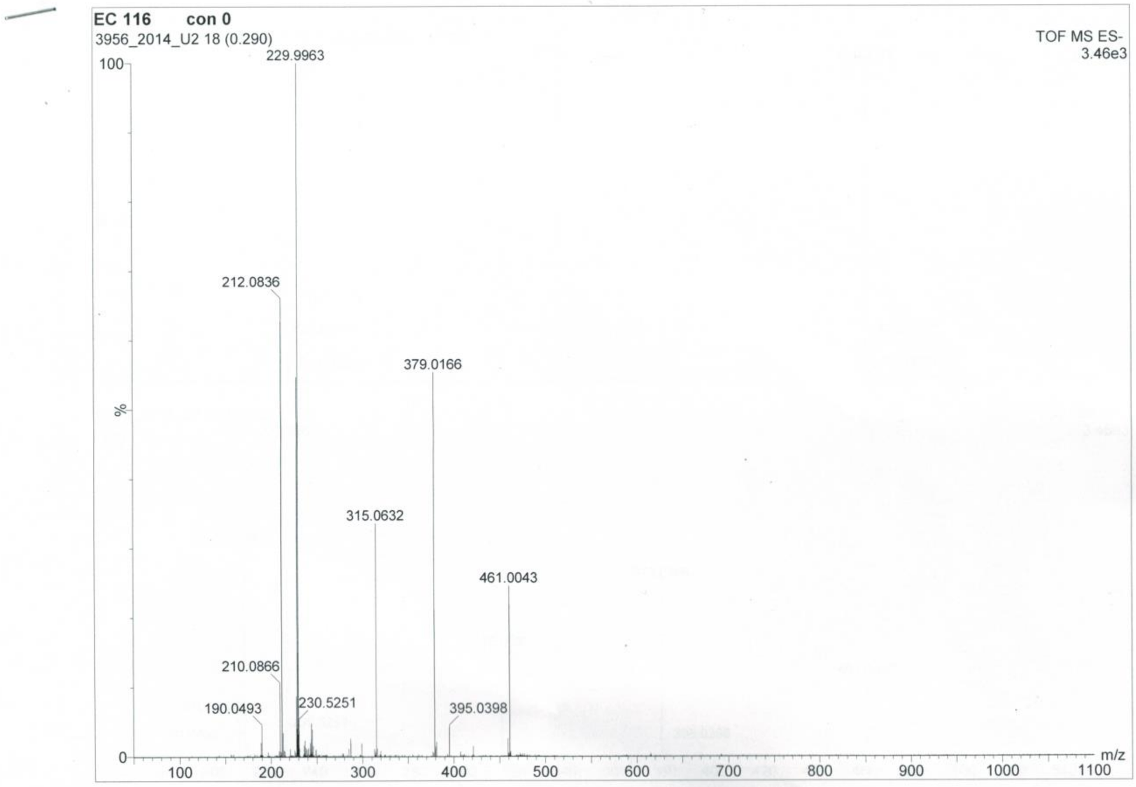

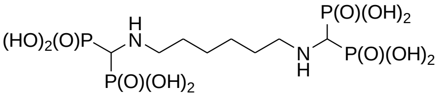

Heksylene-di(aminomethylenebisphosphonic acid) (**16)**

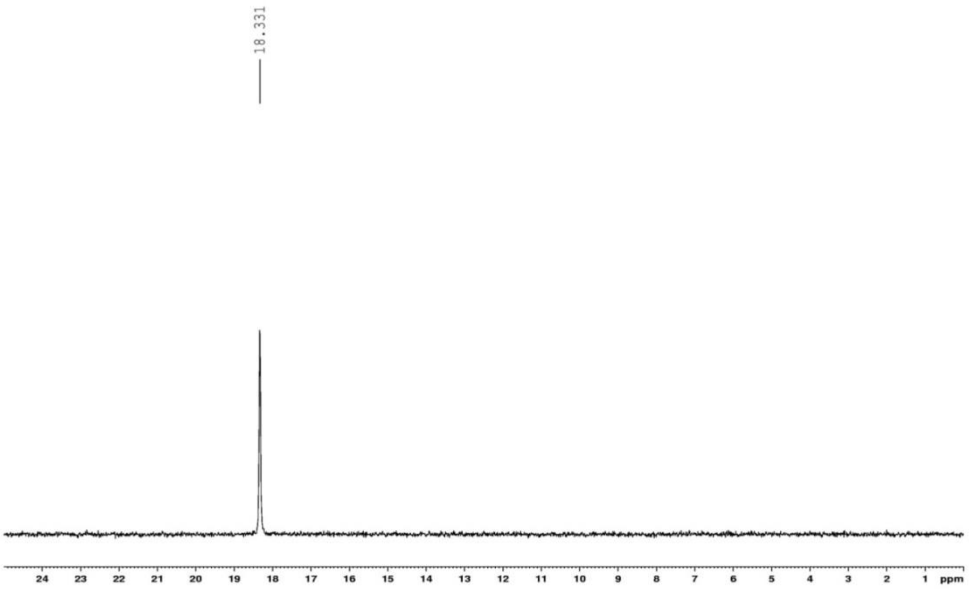

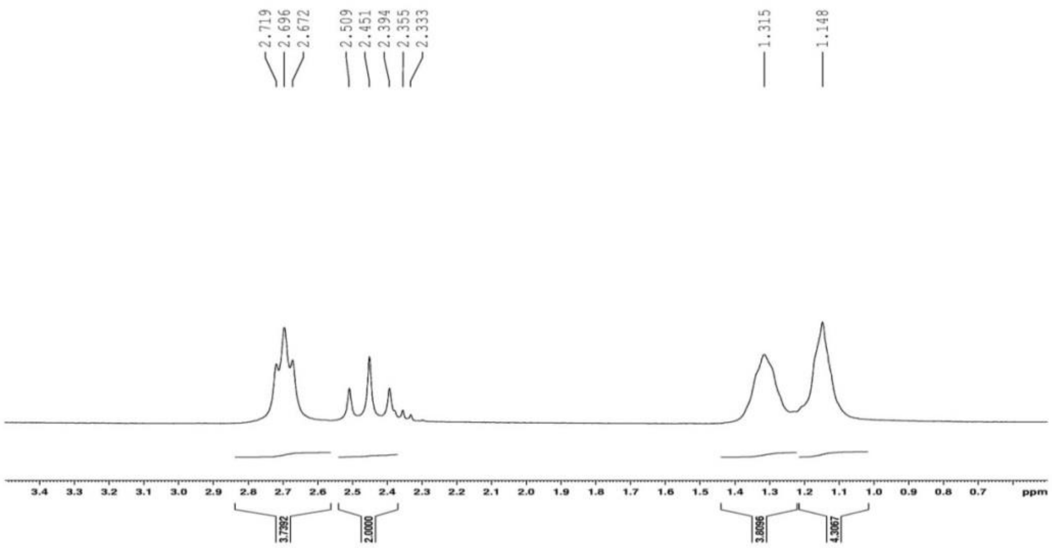

13.12.13 EC_AS_17/3

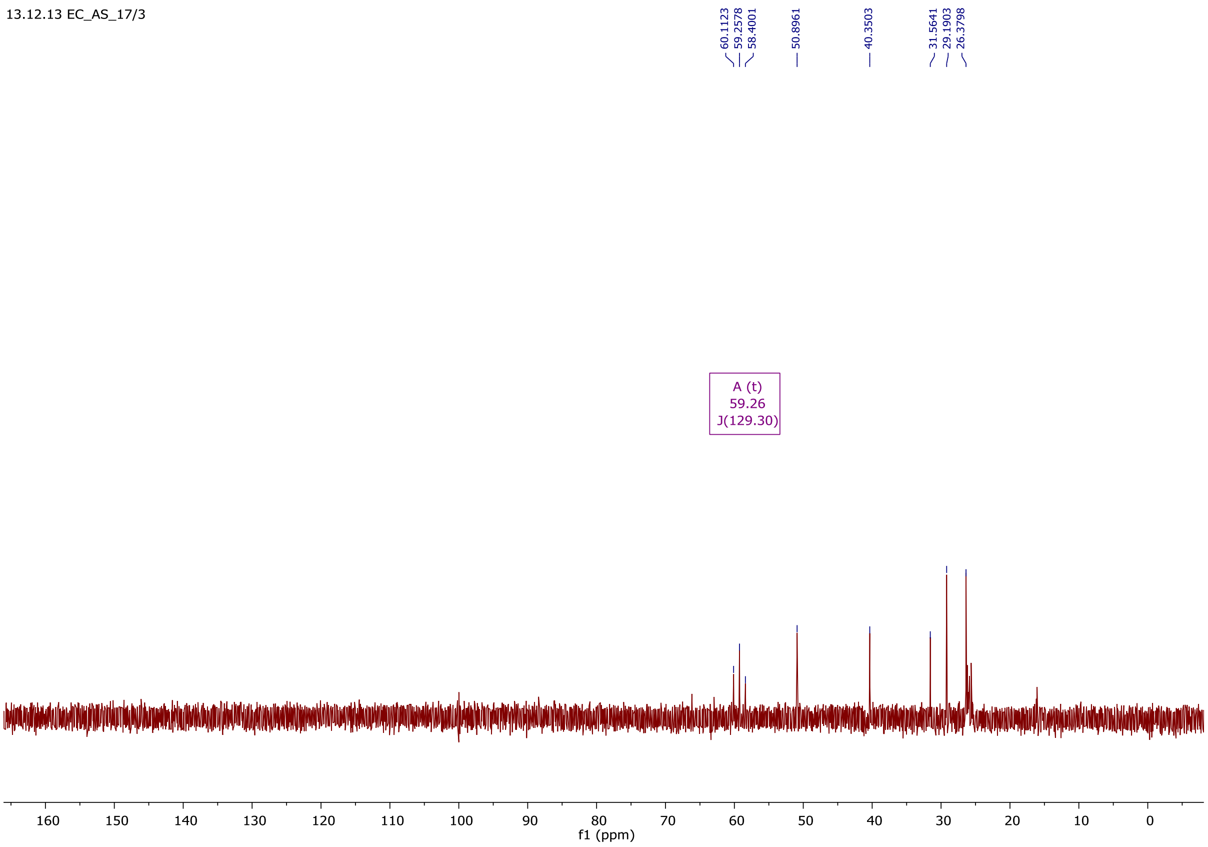

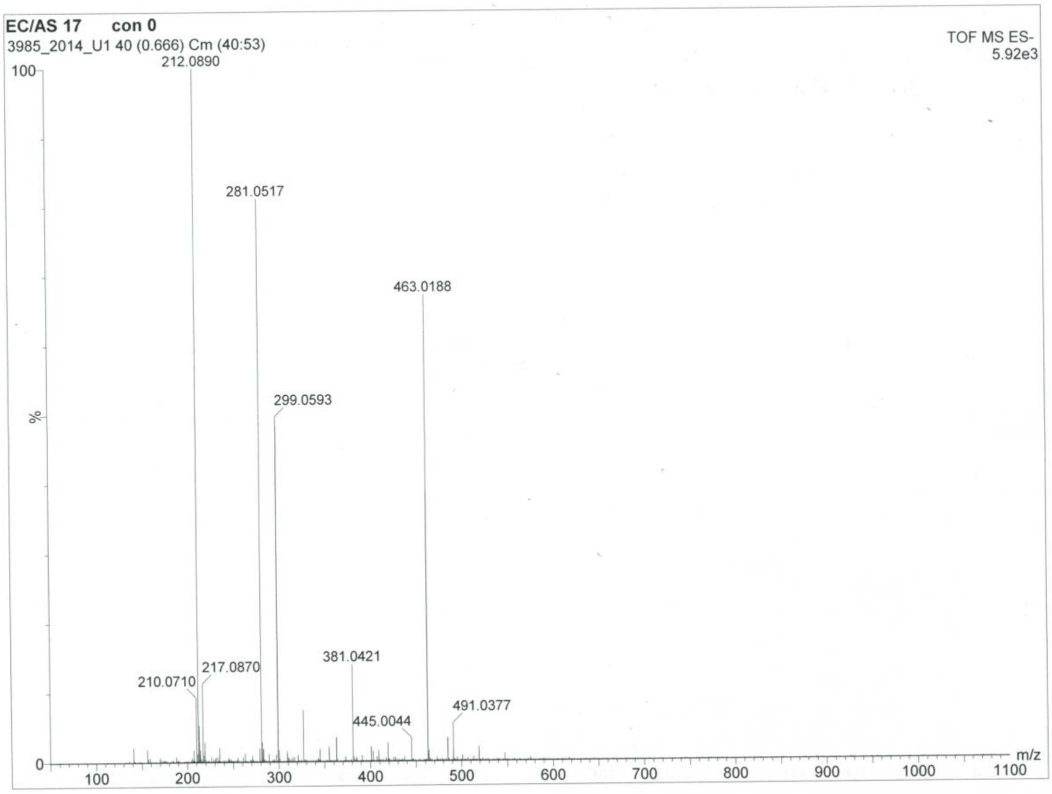
EC/AS 17 con 0
3985_2014_U1 40 (0.666) Cm (40:53)
TOF MS ES-
5.92e3
100
%
0
212.0890
281.0517
463.0188
299.0593
381.0421
210.0710
217.0870
445.0044
491.0377
m/z
100
200
300
400
500
600
700
800
900
1000
1100

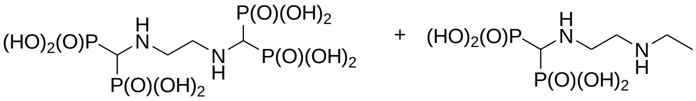

micture of 15 and**17**

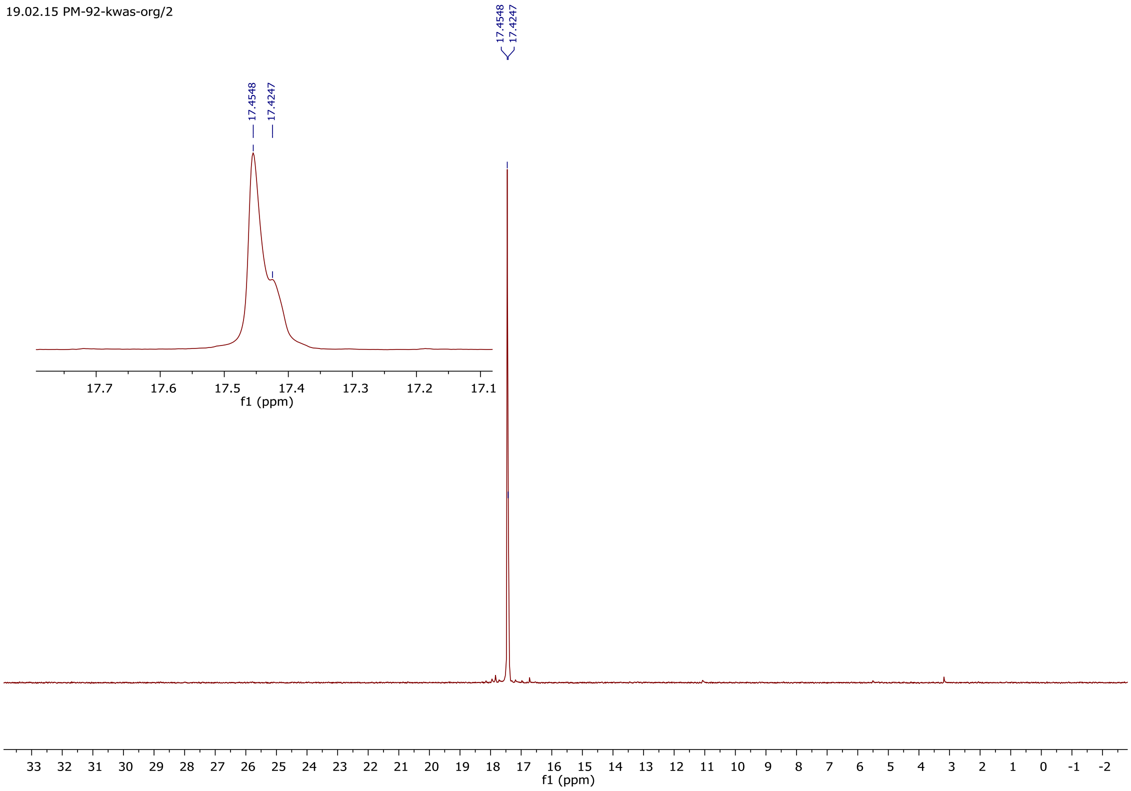

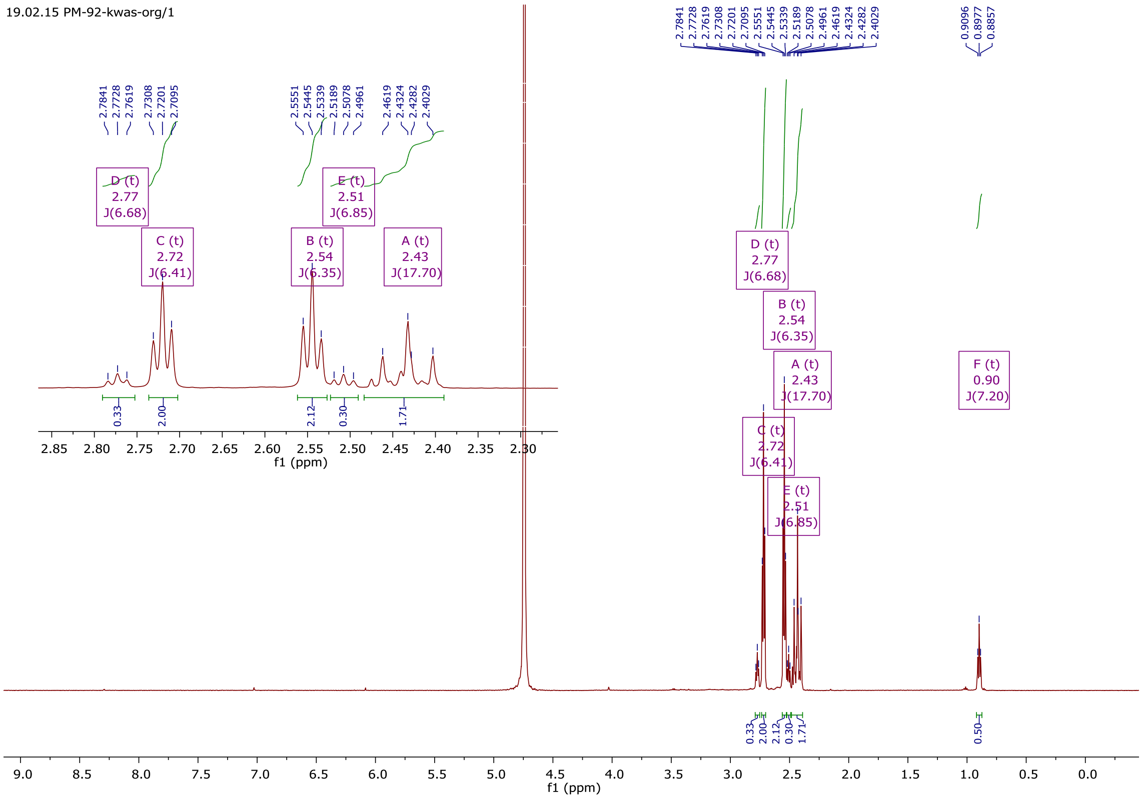

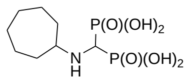

Incadronate

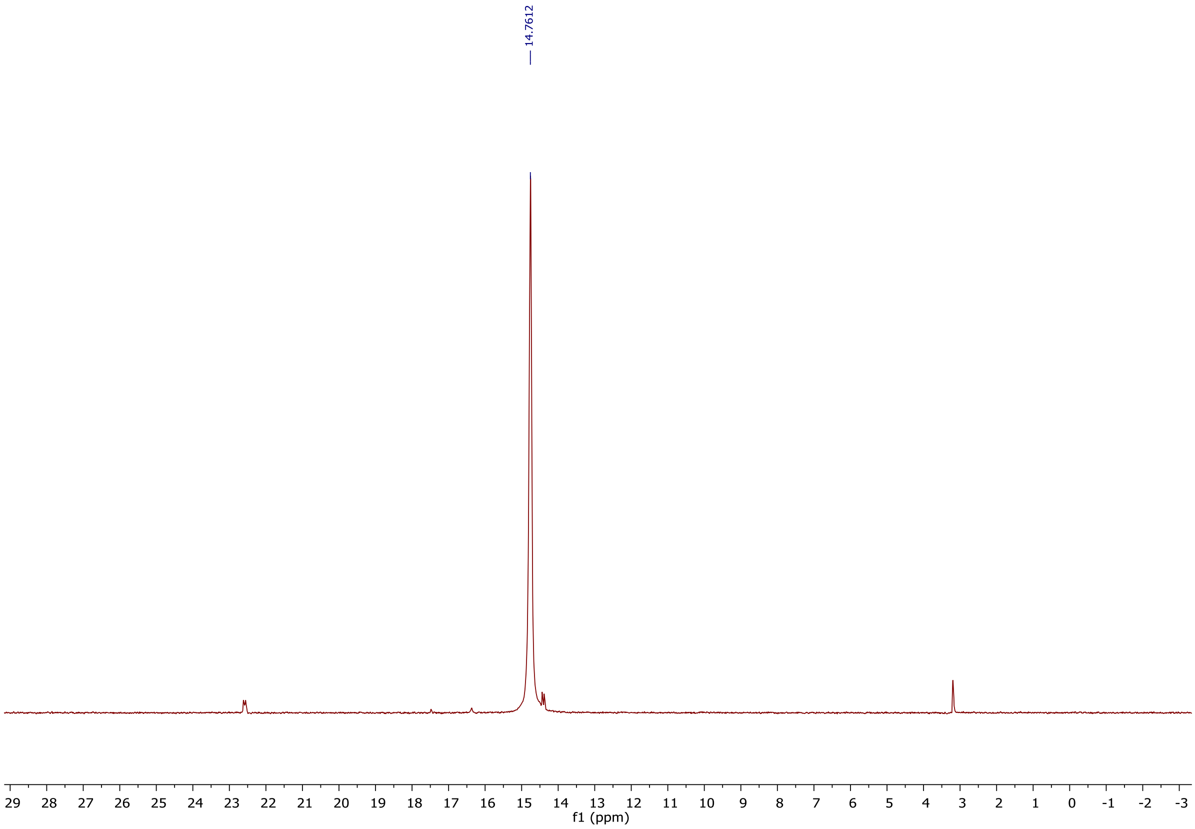

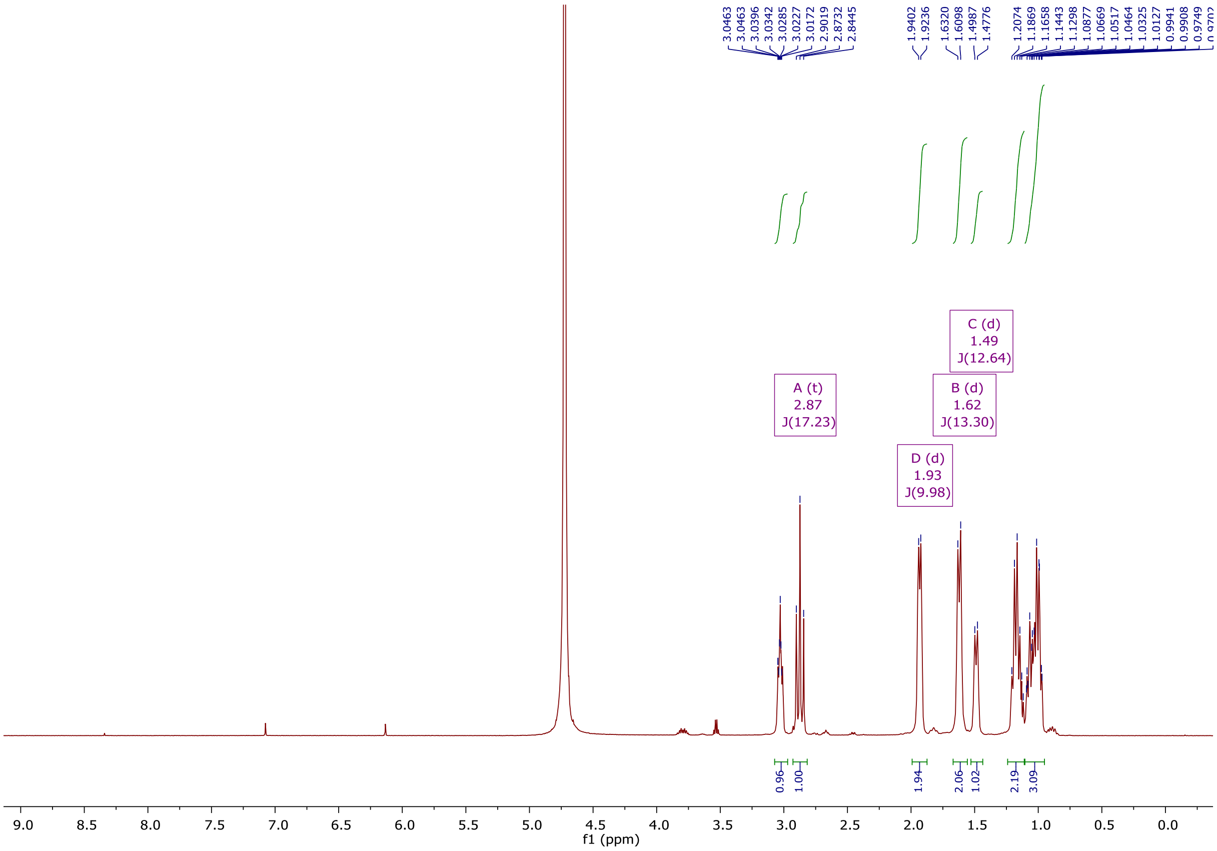

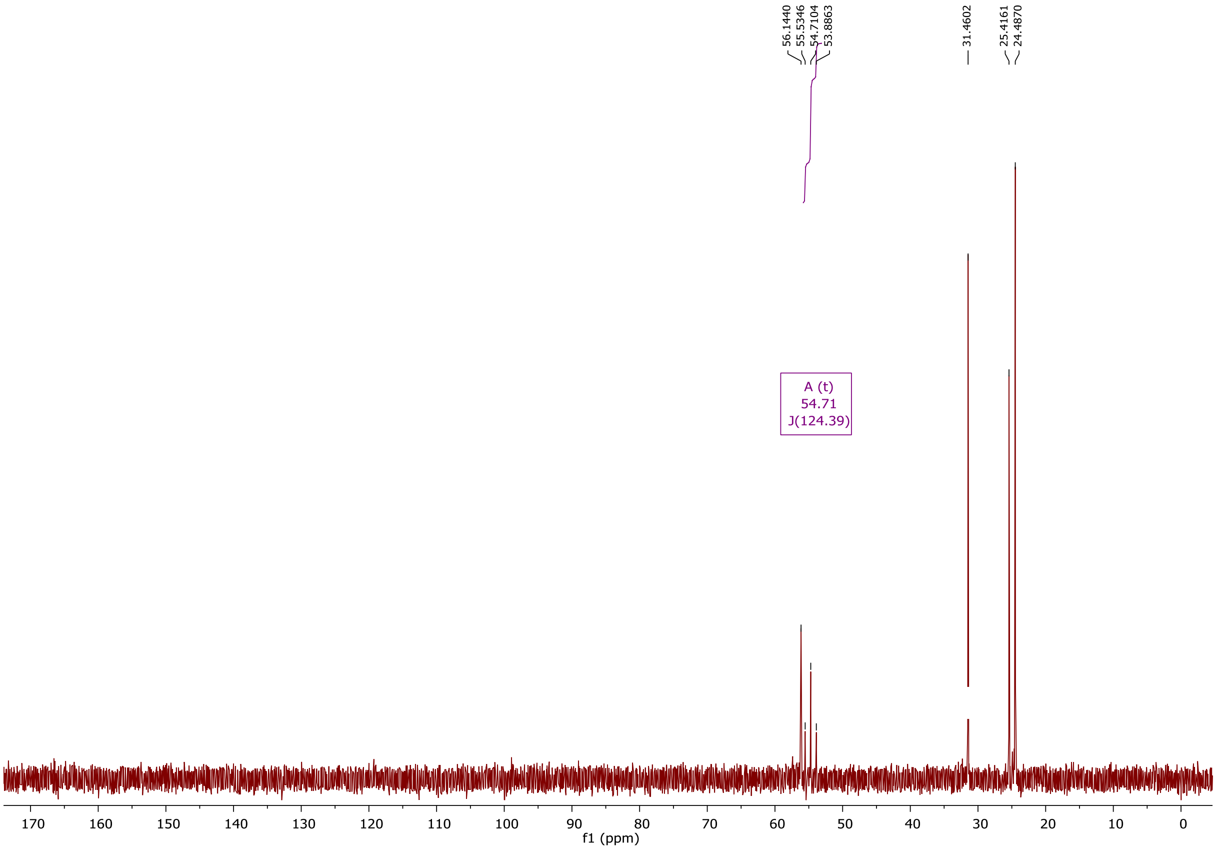

Figure S1. Histology (Delafield staining)

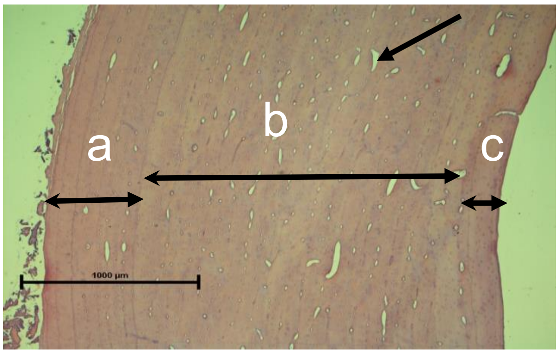

Fragment of femur shaft. From left to right: (a) area of osteogenesis at periosteum side, (b) central area, (c) bone lamella at endosteum site. Arrow shows micropore resulting from osteoresorption.

Figure S2. Examples of erosion bays

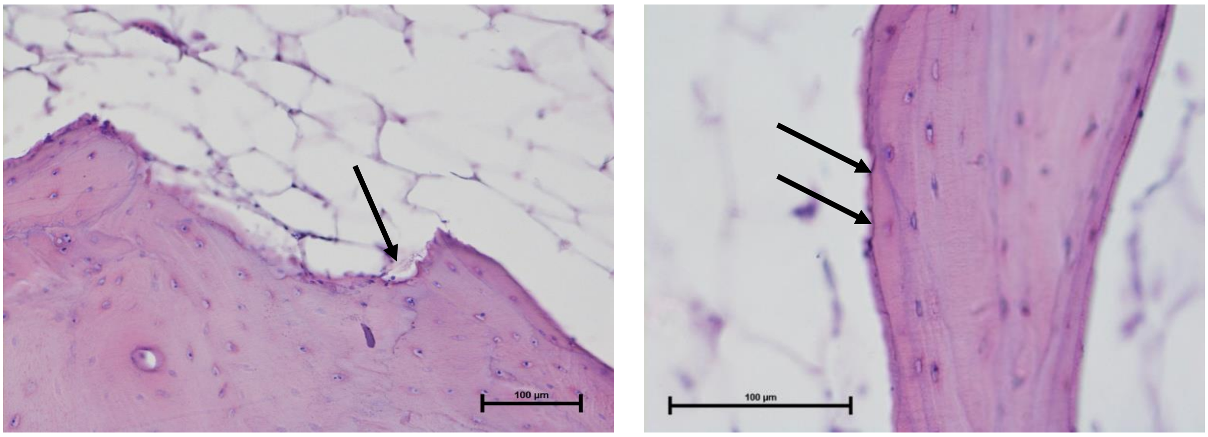

Total hip of femour (magnification 200x)

Proximal tibial epiphysis (magnification 400x)

Figure S3. Dysfunctional course of collagen fibres of tibia (magnification 600x)

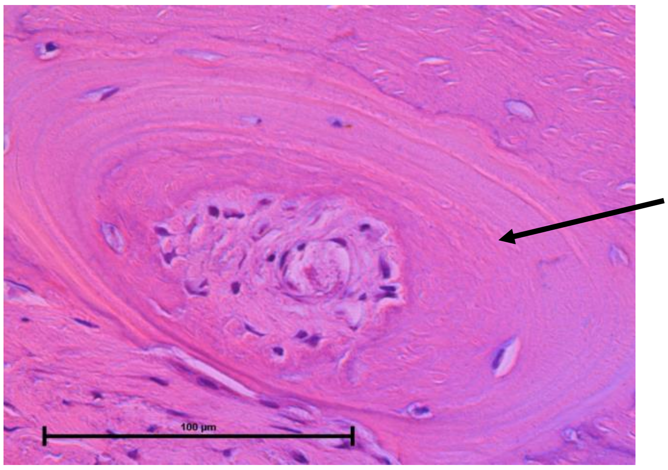

Figure S4. Bone repair (magnification 400x)

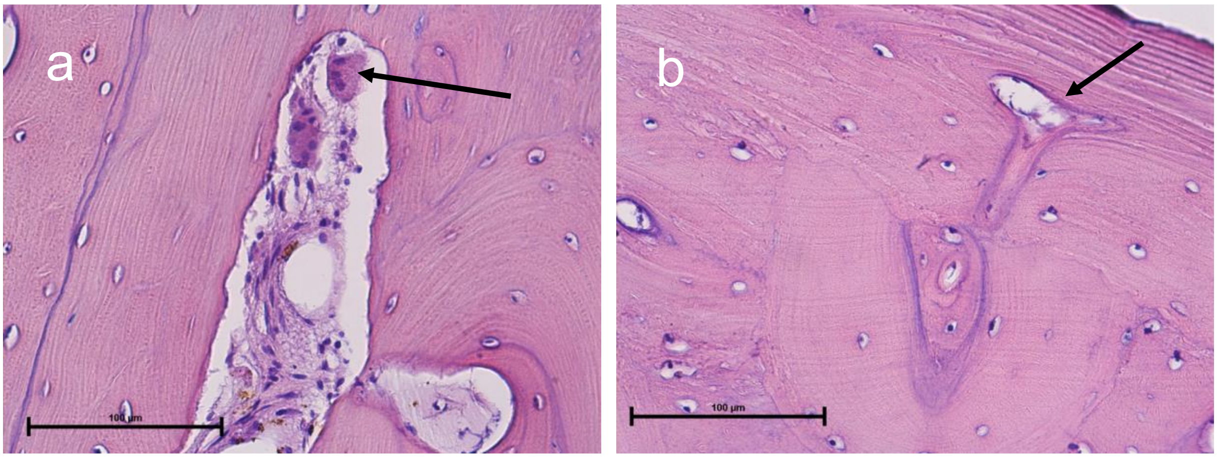

(a) Femur, fragment of intensive reconstruction of bone issue. Arrows indicates multinuclar osteoclast. Osteoclasts are accompanied by angiogenic and ostogenic cells. (b) Tibial bone at endosteum site. Arrow indicates intensive formation of bone lamella.
